# MAD—microbial (origin of) Alzheimer’s disease hypothesis: from infection and the antimicrobial response to disruption of key copper-based systems

**DOI:** 10.3389/fnins.2024.1467333

**Published:** 2024-10-02

**Authors:** Jin-Hong Min, Heela Sarlus, Robert A. Harris

**Affiliations:** Department of Clinical Neuroscience, Center for Molecular Medicine, Karolinska Institutet, Karolinska University Hospital at Solna, Stockholm, Sweden

**Keywords:** Alzheimer’s, microbe, copper, amyloid, Tau, ApoE, aggregation, MAD

## Abstract

Microbes have been suspected to cause Alzheimer’s disease since at least 1908, but this has generally remained unpopular in comparison to the amyloid hypothesis and the dominance of Aβ and Tau. However, evidence has been accumulating to suggest that these earlier theories are but a manifestation of a common cause that can trigger and interact with all the major molecular players recognized in AD. Aβ, Tau and ApoE, in particular appear to be molecules with normal homeostatic functions but also with alternative antimicrobial functions. Their alternative functions confer the non-immune specialized neuron with some innate intracellular defenses that appear to be re-appropriated from their normal functions in times of need. Indeed, signs of infection of the neurons by biofilm-forming microbial colonies, in synergy with herpes viruses, are evident from the clinical and preclinical studies we discuss. Furthermore, we attempt to provide a mechanistic understanding of the AD landscape by discussing the antimicrobial effect of Aβ, Tau and ApoE and Lactoferrin in AD, and a possible mechanistic link with deficiency of vital copper-based systems. In particular, we focus on mitochondrial oxidative respiration via complex 4 and ceruloplasmin for iron homeostasis, and how this is similar and possibly central to neurodegenerative diseases in general. In the case of AD, we provide evidence for the microbial Alzheimer’s disease (MAD) theory, namely that AD could in fact be caused by a long-term microbial exposure or even long-term infection of the neurons themselves that results in a costly prolonged antimicrobial response that disrupts copper-based systems that govern neurotransmission, iron homeostasis and respiration. Finally, we discuss potential treatment modalities based on this holistic understanding of AD that incorporates the many separate and seemingly conflicting theories. If the MAD theory is correct, then the reduction of microbial exposure through use of broad antimicrobial and anti-inflammatory treatments could potentially alleviate AD although this requires further clinical investigation.

## Introduction

There are around 57 million cases of Dementia as of 2019 globally, a number that is expected to increase to over 150 million by 2050 (Nichols et al., 2022). Alzheimer’s disease (AD) is the most common form of dementia and involves a progressive neurodegeneration that begins in the entorhinal cortex of the hippocampus and spreads upwards through the cortex. It is characterized by extracellular plaque formation comprising of amyloid beta (Aβ) and intracellular neurofibrillary tangles (NFT) consisting of hyper phosphorylated tau proteins, that collectively leads to neuroinflammation and ultimately neuronal death (Kumar et al., 2022; Moloney et al., 2021). The affected individual progressively loses memory and aspects of cognitive and executive function until death, a process that has been aptly named “the long goodbye” (Dittbrenner, 1994). Despite the existence of symptomatic treatments there is currently no known cure for AD and the damage is considered irreversible (National Institute on Aging, 2024; Conti Filho et al., 2023).

Despite the pressing need for the scientific community to address AD, we still do not have a definitive understanding of the etiology of this disease. However, the last 30 years of AD research has been dominated by the “amyloid hypothesis” whereby the Aβ peptide is putatively the central causative agent of a series of pathogenic events that ultimately leads to neurodegeneration (Frisoni et al., 2022). This theory is supported by evidence such as autosomal dominant familial AD with mutations in presenilins 1, 2 (PSEN1/PSEN2) and amyloid precursor protein (APP) genes, which increase the levels of the Aβ42 load in the brain with high penetrance (Bateman et al., 2010; Van Giau et al., 2019). In such cases, patients tend to develop AD at an earlier age and are classified as early onset AD (EOAD) that occurs before 65 years of age and comprises around 5% of AD cases (Mendez, 2019). Conversely, most AD cases are considered to be late onset AD (LOAD) that mostly debuts after 65 years. There are notable differences in general disease characteristics such as the faster progression rate in EOAD with more frontotemporal atrophy rather than hippocampal atrophy in LOAD and primarily poorer executive, motor and visuospatial functions in comparison to the primarily poorer memory evident in LOAD (Tellechea et al., 2018). However, both have large degrees of Tau and amyloid pathologies which are considered the key players in the pathogenesis of AD (Hampel et al., 2021). Based on this understanding, IgG1 monoclonal antibodies that directly target various forms Aβ plaques have been developed, resulting in drugs such as Aducanumab (marketed as Aduhelm™) that has been approved for use by the FDA (Dhillon, 2021). This drug held promise as a breakthrough in the treatment of AD that offered advancement over the previous generation of conventional AD symptom delaying drugs (Alhazmi and Albratty, 2022). However, the FDA approval of Aducanumab is controversial, with the ultimate basis of approval predicated on apparent reduction of Aβ plaques and not inhibition of clinical disease outcome (Rabinovici, 2021). The use of PET scanning as a primary method of Aβ plaque detection for Aducanumab has also been criticized for its validity in this application (Høilund-Carlsen and Alavi, 2021). Furthermore, other anti-amyloid drugs such as Solanezumab that target monomeric Aβ mirror such findings of apparently lowered Aβ plaque levels, yet also disappointingly do not slow down cognitive decline (Sperling et al., 2023), a finding that is reflected in the many failed Aβ centric AD treatments to date (Espay et al., 2023).

It therefore appears that the reduction of Aβ levels is insufficient to treat AD, and therefore calls into question the centrality of the “amyloid hypothesis.” Thus, we discuss the important biological functions of the Aβ peptide, which could illuminate the less-than-satisfactory results of these anti-amyloid therapies. Beyond that, we examine related peptides and proteins such as Tau and ApoE, what other pathological changes occur in AD, and understand how they are connected, leading us to pathogens as cause of AD. The important role of pathogens in AD is not a new theory and was first proposed by the Czech physician Oskar Fischer in 1907 (Allnutt and Jacobson, 2020), and was also one of the observations made by the Italian physician Francesco Bonfiglio in 1908, who noted the similarities with manifestations of neurosyphilis (*Treponema Pallidum*) and AD after comparison of brain samples sent to him by Alois Alzheimer (Miklossy, 2015). Since then, many other researchers have strongly argued for further study and trial of antimicrobial therapies for AD due to the accumulated evidence in favor of this theory (Itzhaki et al., 2016; Fülöp et al., 2018; MacDonald, 2006).

Currently, the trajectory of clinical trials indicates little interest in treatment modalities that aim to address this microbial aspect of AD. As of December 2023, out of the 250 current active pharmacological and non-pharmacological NIH clinical trials there is no specific mention of trials stated to target microbes (National Institute on Aging, 2024). The aim of this review is therefore to forward an enhanced form of various microbial theories of AD into a collective and integrative umbrella term of microbial AD denoted as “MAD.” The evidence we present focuses on the notable similarity of the key AD proteins and peptides regarding their primary or alternative functions as antimicrobial agents. We then propose how this could lead to dysregulation of key copper-based systems that are also implicated in AD as a mechanistic hypothesis that integrates other metal ion based theories of AD. We also discuss the evidence surrounding clinical MAD and discuss the broader role of microbes in neurodegeneration and provide suggestions for experimentation to further address potential contradictions in this theory as well as enhance our understanding of the AD. Finally, we provide suggestions of potential treatments based upon the MAD hypothesis. We first discuss the roles of A*β* and Tau as they are part of the central hypothesis of the prevailing AD theory. If MAD is correct, then the primary focus should be mainly in the management of microbial infections within and outside of the CNS, and secondarily in the treatment of downstream consequences, approaches that could be performed simultaneously.

## The biological roles of amyloid beta peptides and their significance in AD

### Neuroprotection and synapse modulation

A*β* is the term given to describe a group of 37–49 long peptides derived from the cleavage of the larger amyloid precursor protein (APP) by β and *γ*-secretases, but not by *α*-secretases (Nunan and Small, 2000; Chen et al., 2017). This differential processing of APP is determined by the cellular localization of each secretase, with α-secretases predominantly expressed on the cell surface, whereas β and γ-secretases mostly localize in the acidic environment of the endolysosome system (Zhang and Song, 2013). The isoforms of Aβ that have gained the most interest in AD research are Aβ_1–42_ and Aβ_1–40_ with Aβ_1–42_ being the most amyloidogenic and liable to fibrillation and aggregation. Aβ_1–40 is_ less so, with an increasing ratio of Aβ_1–42_ to Aβ_1–40_ leading to increased oligomerization and toxicity, possibly due to the inhibitory effect of the Aβ_1–40_ isoform (Qiu et al., 2015; Murray et al., 2009). The difference between Aβ_1–42_ and Aβ_1–40_ lies in the subtle addition of an isoleucine and an alanine at the c-terminus of Aβ_1–42_ that greatly affect its propensity to aggregate. This is due to the hydrophobic nature of isoleucine and alanine, which leads to a less soluble peptide that adopts an equilibrium of monomers, trimers and tetramers, whereas Aβ_1–40_ is mostly monomeric (Chang and Chen, 2014). This aggregation is further enhanced by the addition of excess copper, which accelerates the overall aggregation of Aβ_1–42_ in comparison to Aβ_1–40_ (Jin et al., 2011). This point is highly relevant, as one of the central roles of Aβ is the modulation of synaptic plasticity (Parihar and Brewer, 2010), a process itself that is regulated by copper efflux into the synapse that can influence the excitability of NDMA, AMPA, and GABA receptors as well as vesicular trafficking (D’Ambrosi and Rossi, 2015). The role of copper in this regard is argued to be protective, with copper being released into the synaptic cleft at concentrations of up to 100 μM upon depolarization, inhibiting the activity of postsynaptic NMDA, AMPA, and GABA receptors upon binding, thus preventing overstimulation in a negative feedback system (D’Ambrosi and Rossi, 2015). Synaptic stimulation also leads to the concurrent release of Aβ at the synapse (Parihar and Brewer, 2010).

Following this, the likely role of APP and Aβ isoforms are as copper scavengers, since they both contain copper binding domains with high affinity for copper ions (Kong et al., 2008; Yugay et al., 2016). In particular, Aβ_4-x_ species have been suggested to be primary copper binding agents in the brain’s extracellular spaces that form highly redox stable complexes with copper which can sequester copper from A*β*_1-x_ peptides (Stefaniak and Bal, 2019; Stefaniak et al., 2021). Aβ_4–9_ is an example of one such high affinity copper binding agent that is a main product of the further cleavage of Aβ_1-40_ by neprilysin (Stefaniak et al., 2021). Overall, the effects of various concentrations of Aβ species include neurotrophic effects for rat neurons (Yankner et al., 1990), improved long-term potentiation in mouse hippocampal slices (Puzzo et al., 2008), improvement in neuronal cell viability and more (Parihar and Brewer, 2010). One study reported that the depletion of APP using siRNA or antibodies *in vivo* reduced long-term potentiation (LTP) in mouse brains, crucial to memory formation, that could be rescued by the addition of exogenous human Aβ_1–42_ (Puzzo et al., 2011). More studies using genetic models such as APP null mice or β-secretase beta-site APP-cleaving enzyme 1 (BACE1) null mice display a range of dysfunctions such as lowered synaptic plasticity, restricted neurogenesis and cognitive deficits (Cai et al., 2023). This evidence suggests one key physiological role of Aβ species is in the maintenance of synaptic plasticity via modulation of copper ions. However, it is important to note that optimal levels of Aβ lie within a tight range and that either deficiency or excess can cause impairment (Cai et al., 2023). Furthermore, it is the monomeric form that seems to be protective and not the oligomers or larger aggregates which seem to be cognitively detrimental (Walsh et al., 2002; Giuffrida et al., 2009). In order to understand this drastic variability in isoform and oligomerization state, we need to examine the alternative function of Aβ, namely as an antimicrobial peptide (AMP).

### Anti-microbial function of amyloids

Clues to the function of Aβ antimicrobial properties lie in its structural similarities with the human AMP LL-37 (Lee et al., 2020). LL-37 is a key player of the innate immune system and acts to perforate the cell wall and cytoplasm of bacteria leading to their death, a function achieved by its oligomerization into a hexameric channel shape with a charged core that is necessary for the physical attack of the bacterial cell (Sancho-Vaello et al., 2020; Engelberg and Landau, 2020). This is intriguing, as Aβ also undergoes a similar oligmerization process forming a hexameric barrel which also resembles a channel shape in its protofibrilar state, and which has neurotoxic properties, as demonstrated by Aβ_1–42_ (Lendel et al., 2014; Ciudad et al., 2020). *In vitro* comparison of LL-37 and Aβ_1–40_ and Aβ_1–42_ demonstrates their comparable shared capabilities as a broad spectrum AMPs with bacteriostatic and/or bacteriocidal activity against a variety of bacteria and also fungi (Soscia et al., 2010). In this regard Aβ_1–42_ was more effective than Aβ_1–40_ against a number of selected pathogens, which could suggest why these two isoforms share overlapping yet distinct functions. If AD has an infectious etiology, this may explain the decreased serum Aβ_1–42_: Aβ_1–40_ ratios evident in AD patients, as the antimicrobial forms of Aβ would be preferentially aggregated in the brain (and therefore not enter the serum) as part of an immune response to the detriment of synaptic regulation and neuronal survival (Soscia et al., 2010; West et al., 2021). As previously discussed, the addition of the C-terminal hydrophobic residues isoleucine and alanine in Aβ_1–42_ favors oligomerization into the hexameric barrel shape and thus would increase both its antimicrobial and cytotoxic potential (Lendel et al., 2014; Snyder et al., 1994). This oligomerization processes seems to be evident in other AMPs such as lactoferrin (LTF) (that has been found to aggregate in corneal amyloidosis; Ando et al., 2002). This oligomerization mechanism has been proposed to be a host counteractive measure to bacterial proteases that can degrade monomeric AMPs by facilitating the AMPs to oligomerize into highly protease resistant beta sheet structures (Soscia et al., 2010). In the case of AD, this eventually becomes a problem for even the host. Interestingly, evidence suggests that LL-37 and Aβ_1–42_ may in fact be natural binding partners that would be significant to the spatiotemporal ratios of the two peptides under disease conditions (De Lorenzi et al., 2017).

Aside from direct membrane attack, other mechanisms for amyloid antimicrobial activities include the induction of protein aggregation within the bacteria itself leading to death, conformational changes to microbial proteins by amyloid binding, thereby inhibiting their function or agglutination of microbes into large aggregates (Chen et al., 2022). Furthermore, recent evidence suggests that Aβ has the ability to prevent bacterial biofilm formation in *E. coli* that has been suggested to be potentially important in maintaining the bacteria in a planktonic form that is much more vulnerable to other antimicrobials (Prosswimmer et al., 2024). As biofilms can increase the antibiotic resistance of bacteria up to 1,000-fold (Høiby et al., 2010), therefore the dissolution or prevention of these biofilms is an important part of the host antimicrobial defense and presents a challenge to both the host immune system and also the therapeutic development. The interaction between Aβ and bacteria may be related to the importance of bacterial amyloids in colony and biofilm formation (Zhou et al., 2012), for which it is possible for human Aβ isoforms to interfere with. Evidence suggests that it is possible for the cross-seeding of bacterial and human amyloids akin to how infectious prions propagate, in which one amyloid will alter the conformation of others to adopt a similar structure and aggregate (Friedland and Chapman, 2017). Bacterial curli proteins derived from *E. coli* and other bacteria are integral to their biofilms and have been shown to show to accelerate amyloid-A amyloidosis and induce *α*-Syn aggregation *in vivo* (Lundmark et al., 2005; Chen et al., 2016; Fernández-Calvet et al., 2024). It would therefore be prudent to map the effects of human amyloids vs. microbial amyloids (many of which are biofilm structural components (Fernández-Calvet et al., 2024).

The protective nature of Aβ is also evident *in vivo* as 5XFAD mice [genetic AD mouse model harboring the 5 most prominent familial AD mutations (Oblak et al., 2021)] have significantly better survival profiles than wild type mice in a model of *Salmonella typhirium*-induced meningitis, and as APP knockout mice fare worse than do wildtypes (Kumar et al., 2016). This interesting protective effect is mirrored not only in response to bacteria and fungi, but also to viruses. 5XFAD mice also have better survival compared to wildtype mice with herpes simplex virus 1 (HSV-1)-induced encephalitis, and also young (5–6 week-old) 5XFAD that had received non-lethal intracranial injections of HSV-1 developed amyloid plaque-like senile AD plaques reminiscent of those in human brains after 3 weeks (Eimer et al., 2018). Similar protective effects are evident using a modified HSV-1 (Zhao et al., 2024). Aβ_1–42_ also has been shown to inhibit the replication of H3N2 and H1N1 influenza A viruses that resulted in the aggregation of the virus, reduced epithelial cell infection rates, reduced viral protein synthesis in infected monocytes, and increased the neutrophil response to the virus. This demonstrated direct mechanisms against the virus and also indirect immune-mediated enhancement toward the virus, and in this regard we see again that Aβ_1–42_ was a more effective anti-viral than was Aβ_1–40_ (White et al., 2014). The likely mechanism by which Aβ_1–42_ induces HSV-1 aggregation is by the shared sequence homology of an aggregation-prone region of the HSV-1 glycoprotein B to which Aβ_1–42_ can bind and induce aggregation (Cribbs et al., 2000). This has formed the basis for the successful engineering of synthetic virus-specific antiviral amyloid peptides that target aggregation-prone regions unique to each virus (Michiels et al., 2020). Importantly, although Aβ is generally localized toward the synapses, evidence from AD and Downs syndrome (DS) with early AD symptoms exhibit intraneuronal Aβ_1–42_ accumulation that precedes Tau pathology and extracellular Aβ plaque formation (Takahashi et al., 2017; Welikovitch et al., 2018; Capetillo-Zarate et al., 2012), which would support the hypothesis of Aβ_1–42_ as an intraneuronal infection defense mechanism. Furthermore, it has been established that some of the extraneuronal Aβ is derived from intraneuronal sources (Oddo et al., 2006). The establishment of the chronology of Aβ pathology in AD suggests that an “inside out” hypothesis is likely (Gouras et al., 2014), and we suggest that this supports the interpretation that Aβ pathology is from intraneuronal infection via neurophilic pathogens. In this regard, Aβ is protective against acute brain infection induced by a variety of pathogens, and the long-term effects on the brain of mounting such a chronic defense response to combat persistent pathogens or their related toxins is one of the core themes of MAD.

Overall, the functions of the various isoforms of Aβ are in both the regulation of neurotransmission and in antimicrobial defense. While at first glance the propensity for Aβ aggregation appears to drive AD pathogenesis, this aggregation is one key function of the peptide and other similar amyloids in mounting an antimicrobial response, an effect most evident in the Aβ_1–42_ isoform. Similarly, this sheds light on the nature of other amyloidogenic proteins such as prion proteins that display antiviral properties against influenza A and Japanese viral encephalitis *in vivo* (Chida et al., 2018; Hong et al., 2023). Similarly, alpha-synuclein (*α*-Syn) of Parkinson’s disease is protective against West-Nile virus in the brain (Beatman et al., 2016), and is a key mediator in the inflammatory immune response (Alam et al., 2022), similar to Aβ regarding H1N1 infection mentioned earlier. We will later discuss the evidence of microbes in the pathogenesis of AD in patients, but first it is important to understand the role of tau protein, another prominent player in the pathogenesis of AD as this protein also bears similarities with Aβ, despite different functional roles.

## The biological roles of Tau and its significance in AD

### Tau and neuronal microtubule and chromatin stability and stress granule formation

Along with Aβ, the tubulin associated unit (Tau) protein is considered a key player in AD pathogenesis as it forms the basis of intra-neuronal NFTs evident in AD, and displays a higher correlation with clinical AD symptom severity than does Aβ (Congdon et al., 2023). Unlike Aβ localization in the synapse, Tau is mainly located in the axonal compartment and also in the nucleolus, but is also found in lower concentrations in the dendrites and mitochondria (Arendt et al., 2016; Guo et al., 2017). This localization is directly related to the key role of Tau in the maintenance of the complex architecture of the neuron by stabilization of microtubule (MT) bundles through interactions with tubulin subunits (Avila et al., 2004). The structure of this family of 352–441 amino acid long proteins generally consists of 4 subunits (Goedert et al., 1989), an N-terminal region that is suggested to be involved in MT stabilization and that also contains a copper binding domain (Derisbourg et al., 2015; Lukács et al., 2019), a proline rich region, a MT recognizing and binding domain, and finally the C-terminal region (Brandt et al., 2020). In general, upon binding Tau protects MTs from depolymerization and is highly likely to be a factor in the induction of MT polymerization (Barbier et al., 2019), thus playing a key role in MT dynamics and resulting in increased MT growth rates and reduced dissociation rates (Murphy et al., 1977; Trinczek et al., 1995). In addition to this, Tau acts via bridges between MTs and actin via its tubulin binding domains that facilitate formation of actin-tubulin cytoskeletal structures (Elie et al., 2015).

Another function of Tau is revealed by its presence in the nucleus, where it has been reported to play roles in heterochromatin organization and chromatin stability by binding and interaction with heterochromatin, as well as modifying the liquid–liquid phase separation (LLPS) characteristic of chromatin components (Abasi et al., 2024). Low concentrations of Tau condense chromatin, thus protecting the DNA from digestion, although the full mechanisms for this are currently unknown (Abasi et al., 2024). One aspect of this function, however, is the inhibition of deacetylases such as tubulin deacetylase in the case of MTs and histone deacetylase 6 (HDAC6) in the case of heterochromatin (Perez et al., 2009).

When cells become acutely stressed, one of the main responses is the formation of stress granules. These are comprised of temporarily untranslated mRNAs that are polymerized by RNA-binding proteins to form membrane-less phase separated messenger ribonuclear protein complexes which transiently halt mRNA translation under unfavorable conditions such as cell stress, but this may be resumed later (Wang et al., 2020). In this regard, Tau promotes stress granule formation that is likely related to its accumulation in the somatodendritic compartment (Vanderweyde et al., 2016), a process that is due to its interaction with the RNA binding protein T cell intracellular antigen 1 (TIA1) and other RNA binding proteins (Vanderweyde et al., 2016; Kavanagh et al., 2022).

In general, it appears that Tau plays a multifunctional protective role in the integrity of vital cellular components such as the MTs, DNA and also mRNA. However, Tau can also undergo a variety of post-translation modifications such as phosphorylation and acetylation (Barbier et al., 2019; Caballero et al., 2021). This phosphorylation of Tau greatly changes its functions, and its hyperphosphorylated state in NFTs is considered one of the hallmarks of AD (Gong and Iqbal, 2008). During AD, the phosphorylation of Tau can occur on the numerous serine, threonine and tyrosine residues of the protein, the progressive phosphorylation of which correlates with the Braak Stages of AD pathology and can take place in over 40 sites (Neddens et al., 2018; Gong et al., 2005). The phosphorylation can be initiated by a variety of mechanisms that include kinases such as glycogen synthase kinase-3β (GSK-3β) and cAMP-dependent protein kinase (PKA) (Gong and Iqbal, 2008). Furthermore, synaptic activity can also induce Tau hyperphosohorlyation in a zinc-dependent process that inhibits the activity of protein phosphatase 2 (PP2A) (Sun et al., 2012). Furthermore, Aβ is able to promote the hyperphosphorlyation of Tau in both the cytosolic and synaptic compartments, which has been suggested as a potential explanation of the prion-like “seeding” hypothesis of Tau transmission inter-neuronally, accounting for the phosphorylated Tau detected in the CSF of AD patients (Wu et al., 2018; Ferreira et al., 2014; Bennett et al., 2017). The consequences of phosphorylation and eventual hyperphosphorylation of Tau can lead to the loss of its normal homeostatic functions and is increasingly cytotoxicity (Liu et al., 2020). This is attributed to the loss of MT stabilization and even destabilization of MTs, and also destabilizing interactions with actin filaments, as the phosphorylation of Tau at specific sites reduces the capacity of Tau to bind MTs that contributes to neuronal structural instability and neurodegeneration (Johnson and Stoothoff, 2004; Canudas et al., 2005). Furthermore, under cell stress conditions such as heat shock stress, non-phosphorlated Tau is localized in the nucleus to protect DNA. However, Tau hyperphosphorylation results in a loss of nuclear function and translocation out of the nucleus in a manner reminiscent of ALS-related TDP-43 translocation (Abasi et al., 2024; Ulrich et al., 2018). Direct evidence for this exists in the decline of nuclear Tau in the CA1 region and dentate gyrus of AD brain samples, where nuclear Tau is almost absent in neurons with NFTs and some without (Hernández-Ortega et al., 2016). Furthermore, hyperphosphorylated Tau aggregates can stimulate inflammatory signaling in macrophages via toll-like receptor 4 (TLR4) (Meng et al., 2022). Bearing these processes in mind, we will now discuss what the potential cause of this hyperphosphorylation of Tau is in AD.

### Antimicrobial activity of Tau hyperphosphorylation

The connection between infection and AD and Tau phosphorylation is supported by evidence such as HSV-1 infection inducing Tau phosphorylation in an AD-like manner that is due to the viral stimulation of GSK-3β and PKA (Wozniak et al., 2009). Furthermore, phosphorylated Tau plays a role in the antiviral response by potentially acting as a danger signal (Powell-Doherty et al., 2020), and binding sites on Tau proteins have been identified as components for the design of AMPs that when combined with a nuclear localization signal or laminin-receptor derived peptides, display strong activity against *E. coli* and *S. aureus*, and have been speculated to be an important part of the innate immune response (Kobayashi et al., 2008). Furthermore, bacterial DNA has been shown to promote Tau aggregation (Tetz et al., 2020). However, the hyperphosphorylation of Tau and associated antimicrobial effects are far less studied than with Aβ and thus presents a gap in research knowledge.

Tau phosphorylation and Aβ aggregation are possibly two interlinked mechanisms of antimicrobial protection which could be expected as the interactions between Aβ and Tau are also connected at a molecular level via GSK-3 due to its role in the generation of the Aβ peptide and phosphorylated Tau (Sayas and Ávila, 2021), a process that can be triggered by the viral stimulation of GSK-3β (Wozniak et al., 2009). In this regard, HSV-1 infection of SHSY-5Y Neuroblastoma induce a 3-fold increase in levels of phosphorylated Tau, with GSK-3β responsible for the phosphorylation of Threonine-212 and Serine(s)-202, 396, and 404 in conjunction to PKA phosphorylation of Serine 214 (Wozniak et al., 2009). This could be related to the established role of GSK-3β in innate immunity as it is necessary for I_k_B phosphorylation leading to downstream NF-κB induction that is key for pro-inflammatory cytokine signaling, and also the induction of a type 1 IFN antiviral responses, forming immunocomplexes with factors such as with TANK-binding kinase 1 (TBK1), that is responsible for IFN regulatory factor 3 phosphorylation and subsequent signaling and other factors (Marineau et al., 2020; Qin et al., 2016). This would suggest that Tau phosphorylation is part of an additional element of a GSK-3β mediated immune response, especially considering the antimicrobial properties of Tau fragments (Kanagasingam et al., 2022). It is important to note that the stimulation of GSK3β leads to phosphorylation of insulin receptor substrate protein 1 (IRS1) affecting insulin like growth factor 1 signaling (IGF-1) and resulting in downstream insulin resistance (Leng et al., 2010; de la Monte and Wands, 2008).

It therefore appears likely that Tau and Aβ can be induced into states that promote a generalized antimicrobial response, supported by the ability of a variety of bacterial DNAs to promote Tau aggregation *in vitro* (Tetz et al., 2020). This could explain why infection of mice with oral *Trepomnema denticola* bacteria induces Tau hyperphosphorylation and Aβ accumulation in mice in the hippocampus with neuronal loss (Wu et al., 2022), and why filtrates of *Helicobacter pylori* bacteria induce Tau hyperphosphorylation in mouse neuroblastoma cells and rat brains via GSK-3β activation (Wang et al., 2014). It is important however, to establish direct evidence for the antimicrobial effect of Tau in these cases. Another recent study has demonstrated the enhanced antimicrobial activity of a phosphorylated Tau fragment against *P. gingivalis* in contrast with its non-phosphorylated state (Kanagasingam et al., 2022). This combined evidence suggests that phosphorylation of Tau is part of an immune response toward a broad range of pathogens or their toxins, similar to Aβ, both of which are diverted from their normal roles in neuronal homeostasis. Aβ and likely Tau are not the only antimicrobial defenses available to neurons, as Apolipoprotein E (ApoE) is also diverted from its main function in lipid homeostasis for use as an antimicrobial agent.

## The biological roles of Apolipoprotein E and its significance in AD

### Apolipoprotein E, cholesterol, and neuronal support

Another key glycoprotein relevant to AD pathogenesis is Apolipoprotein E (ApoE). This protein plays a central role in lipid metabolism whereby it associates with lipoproteins of different densities in an isoform-dependent manner and assists in their binding to different cell surface receptors, thus facilitating the transport of cholesterol and lipoproteins. There are 3 main isoforms: ApoE2, ApoE3, and ApoE4 in humans which represent 8, 77, and 15% allelic frequency, respectively (Huang and Mahley, 2014). In AD, ApoE4 is considered the disease-enhancing variant and a major risk factor in AD; ApoE3 is the normal variant; and ApoE2 is the protective variant for which humans can either be homozygous for each, or a heterozygous mix. ApoE4 homozygotes are generally at the highest risk for AD, with ApoE2 homozygotes being the most protected (Kim et al., 2009). These phenotypic differences lie in single amino acid changes that greatly affect the structure and therefore binding affinity of ApoE, with ApoE3 containing Cys-112 and Arg-158, ApoE2 having Cys-112 and Cys-158, and finally ApoE4 with Arg-112 and Arg-158 (Huang and Mahley, 2014). The replacement of Cys-112 with Arg-112 in ApoE4 increases its binding affinity to low and very low density lipoproteins (LDL, VLDL), whereas ApoE3 and ApoE2 prefers high density lipoproteins (HDL) that are richer in cholesterol (Weisgraber, 1990). The exact reasons for this have not been fully established but been suggested to be the effect of Arg-112 making ApoE4 in a tighter locked conformation than ApoE3 or ApoE2, thus hindering the lipid insertion site (Chen et al., 2021; Frieden et al., 2017).

When lipid bound, ApoE generally facilitates internalization of the complex via ApoE receptors such as apolipoprotein E receptor 2 (ApoER2), low-density lipoprotein receptor-related protein 1 (LRP1), Low-density lipoprotein receptor (LDLR), very low-density lipoprotein receptor (VLDLR) and other members of the LDL receptor-related protein (LRP) family of receptors (Herz, 2009; Fernández-Calle et al., 2022). Crucially, ApoER2 signaling in the brain is responsible for both early stage neuronal development and also adult synaptogenesis and learning in the “Reelin pathway” that is primarily activated through ApoE binding to ApoER2, for which ApoER2 is heavily expressed in the hippocampus and neocortex, in particular in granule cells and pyramidal cells (Fernández-Calle et al., 2022).

The majority of the brain ApoE (75–80%) is derived from astrocytes, with 15–20% in neurons and lesser contributions from oligodendrocytes, endothelial cells and microglia (Blumenfeld et al., 2024). In particular, the astrocytic ApoE (complexed with cholesterol) promotes synaptogenesis (Mauch et al., 2001), with ApoE accumulating near the synapses (Konings et al., 2021). It is important to note the necessity for cholesterol in maintaining basic neuronal functioning such as myelination, synaptic development and growth, for which the bulk is supplied by astrocytic ApoE (Karahan et al., 2021). In addition to this, astrocytic ApoE-HDL particles contain a diverse range of neuronal regulatory miRNAs that ultimately promote neuronal functioning (Li et al., 2021). It would be of interest to investigate whether the miRNA contents of ApoE, aside from being cell specific, are also isoform-dependent. Furthermore, healthy & neuromeyelitis opitica spectrum disorder patient derived astrocytic ApoE rich vesicles both decrease neuroinflammation *in vivo* (Jiang et al., 2024). ApoE also regulates copper via ATP7A copper transporter trafficking. In this regard ApoE4 is considered detrimental, resulting in impaired copper export and elevated copper accumulation in astrocytes and neurons *in vitro.* This has been suggested to promote copper deficiency in AD, as astrocyte ApoE4-mediated copper retention could contribute to reduced neuronal copper availability since astrocytes are key to regulating brain copper levels (Scheiber and Dringen, 2013; Blades et al., 2024). ApoE thus plays an important homeostatic role in the regulation of lipid contents in the CNS and the distribution of cholesterol and copper homeostasis in the CNS.

However, some key changes in cellular ApoE expression occur during AD. For instance, single nucleus sequencing of the entorhinal cortex of the hippocampus from AD brains reveals a repression of ApoE expression in astrocytic subpopulations and also oligodendrocyte precursor cells, but an increase in microglial expression (Grubman et al., 2019). This shift in ApoE origin in the CNS raises questions as to the differential effect of ApoE from these sources. For instance, under inflammatory conditions microglia increase their secretion of ApoE whereas astrocytes do not, and microglia release a single ApoE species whereas astrocytes release two that are under different states of glycosylation (Lanfranco et al., 2021). In the case of microglial inflammation-induced ApoE, it is not understood what types of lipoproteins they are vectoring or if they are unbound, nor their contents or miRNA load if bound. Further clarification and comparison between the different cellular ApoE sources under homeostatic and stress conditions would be interesting. Overall, it would appear that upon inflammatory bacterial LPS challenge, microglia take up a more active role with regards to ApoE, presumably as part of an immune response.

### Antimicrobial activity of ApoE

As with Aβ and Tau, ApoE also has an alternative function as a potent antimicrobial. *In vitro* studies confirm the antibacterial effect of ApoE against *Pseudomonas aeruginosa* and *E. coli*, with ApoE being able to kill bacteria and agglutinate them and also to bind and neutralize lipopolysaccharide (LPS) (Petruk et al., 2021). This activity was predicted *in silico* to be in part due to the existence of multiple AMP domains on the ApoE molecule, likewise sharing many similarities in mechanism of action such as bacterial membrane disruption similar to other AMPs described earlier (Petruk et al., 2021). Furthermore, these results have been extended *in vivo* as anti-endotoxin effects with lowered inflammatory NF-κβ responses in mice injected with heat-killed *P. aeruginosa* (Puthia et al., 2022). Conversely, ApoE-deficient mice have increased mortality due to fungal infection (Vonk et al., 2004).

Both AMPs and aggregating proteins are richer in hydrophobic residues (Zhang et al., 2021; Navarro and Ventura, 2022) (e.g., Aβ_1–42_’s additional hydrophobic domains for increased aggregation and AMP activity discussed earlier), that has been suggested to be the mechanism underlying the ability of ApoE to reduce NF-κβ activation of LPS by blocking LPS binding to the hydrophobic pockets on TLRs via aggregation (Puthia et al., 2022). Furthermore, the receptor binding domain of ApoE interferes with viral attachment and infection by *P. aeruginosa*, *S. aureus*, HSV-1 and HSV-2 (Dobson et al., 2006), with synthetic ApoE peptides having been developed with various antibacterial and antiviral activities (Azuma et al., 2000). ApoE also induces an anti-inflammatory phenotype in macrophages (Baitsch et al., 2011), that in conjunction with LPS blocking ability reveals an important immunological motif, namely to be simultaneously antimicrobial yet also anti-inflammatory. This is a feature shared by other AMPS such as lactoferrin and LL-37 (Luo and Song, 2021; Berthon et al., 2022; Kahlenberg and Kaplan, 2013). This could be important in the resolution of inflammation by eliminating the causal factor (i.e., pathogens) while simultaneously promoting healing. There appears to be mechanistic and functional similarities between ApoE and other AMPs that raises interesting insights into the mechanisms of sterilizing healing in our immune system.

Concerning the ApoE4 variant, regardless of AD diagnosis, older adults that are ApoE4^+^ display a greater level of Aβ accumulation that is not evident in ApoE3^+^ or ApoE2^+^ individuals (Lim et al., 2017), and AD patients with the ApoE4 genotype (^+/+^ or ^+/−^) display higher levels of intraneuronal Aβ accumulation compared to controls, indicating a baseline detrimental cognitive effect (Yamazaki et al., 2019). To date, there have been many studies that have covered the negative impact of the ApoE4^+^ variant, and they are generally linked with dysplidiemia, glial dysfunction with lipid accumulating in microglia and astrocytes (Haney et al., 2024; Litvinchuk et al., 2024), oligodendrocyte myelination impairment (Blanchard et al., 2022), lowered vascular integrity (Montagne et al., 2021), mitochondrial dysfunction (Schmukler et al., 2020) and pathological interaction with Tau and Aβ, many of which have been covered in numerous reviews (Blumenfeld et al., 2024; Yamazaki et al., 2019; Safieh et al., 2019; Sun et al., 2023). The significance of ApoE4 is such that ApoE4^+/+^ carriers could represent a distinct genetic AD variant based on very high penetrance (Fortea et al., 2024). However, to gain a more complete picture we should consider in what situations the ApoE4^+^ allele carries some evolutionary advantages, better protection against hepatitis C infections and enhanced protection against liver cirrhosis in hepatitis patients with lowered viral loads (Nascimento et al., 2020). Furthermore, ApoE4^+^ carriers display higher fertility rates in areas of high pathogen exposure (van Exel et al., 2017), suggesting an evolutionary adaptation against a variety of pathogens that would be selected for in our evolutionary past as ApoE4 is considered the original ancestral phenotype (Fullerton et al., 2000).

Curiously, despite ApoE4 being protective against hepatitis C infection, it is not against HSV-1. In this regard it facilitates HSV infection and entry into the CNS and is a known major risk factor in the development of AD (Linard et al., 2020; Burgos et al., 2003). *In vivo* evidence corroborates this finding, such that mice with an ApoE4 background display an over 10x higher HSV-1 load in the brain compared to mice with an ApoE3 background, strikingly with viral DNA also being detected in the ovaries, spinal cord and trigeminal ganglia in infected ApoE4 mice (Burgos et al., 2006). This could in part be due to the lower levels of ApoE in the CNS of ApoE4 carriers (Flowers and Rebeck, 2020), thus offering less overall antimicrobial protection, but also mechanistically as ApoE4 prevents HSV-1 cellular attachment but crucially not viral entry or replication, and can also be incorporated into the HSV-1 virion that can enhance release from the cell (Feng et al., 2023), which would be an issue for viral AD.

Since ApoE, Aβ and Tau each play their own homeostatic role yet also alternatively function as antimicrobials, it is no surprise that they all have a high degree of mutual interaction. For instance, both Aβ and Tau can both be bound by ApoE (Chen et al., 2017; Huang et al., 1995), and ApoE can regulate their clearance and metabolism by low-density lipoprotein receptor-related protein 1 (LRP1) (Cooper et al., 2021; Van Gool et al., 2019), for which ApoE4 appears to be deficient (Narasimhan et al., 2024). Aβ and Tau can also trigger upregulation of the type-1 interferon response genes (Sanford and McEwan, 2022) (similar to ApoE as discussed earlier) that would suggest antiviral synergy. Aβ and Tau can directly interact to form complexes via shared epitopes that facilitate cross seeding and mutual enhancement of their toxicities (Tripathi and Khan, 2020; Zhang et al., 2021). While details of these interactions between amyloidogenic peptides and proteins (such as Aβ and Tau) with ApoE is complex, and have been covered in numerous reviews (Loch et al., 2023; Wisniewski and Drummond, 2020; Raulin et al., 2022). It is therefore important to point out that their mutual connections should be taken into account when considering their overlapping functions as antimicrobials.

It seems that having multiple types of AMPs would confer a broader spectrum of pathogen resistance, as these AMPs display varying degrees of pathogen specificity. For instance, Aβ displays superior inhibition of *Candida albicans* and *Staphylococcus epidermis* compared to LL-37. While LL-37 is superior against *P. aeruginosa*, Aβ struggles to neutralize it, even at high concentrations (Soscia et al., 2010). It is therefore likely that these partially redundant AMPs in the CNS could compensate for each other’s weaknesses, yet still co-operate when encountering a mutually specific pathogen. It is still unknown to what range of pathogens ApoE confers protection against, and whether this is in an isotype-specific manner or is dependent on fragmentation state or post-translational modification.

## Lactoferrin: antimicrobial and anti-inflammatory agent

Although we briefly mentioned lactoferrin (LTF), we will elaborate on it as LTF is a multifunctional polypeptide implicated in AD pathogenesis. LTF is a protein present in high quantities in breastmilk that exerts iron binding capabilities due to its structural similarity to transferrin, a key iron transporting protein (Wally and Buchanan, 2007). It displays broad antimicrobial abilities through various mechanisms including the sequestration of iron required for bacterial growth and direct membrane permeation ability, which are similar to the other AMPs mentioned earlier such as Aβ and LL-37 (Cao et al., 2023). Other inhibitory mechanisms include the ability of LTF to directly inhibit the protease activity of secreted bacterial gingipain enzymes of *P. gingivalis*, thus leading to growth inhibition and biofilm formation (Dashper et al., 2012). LTF can also be hydrolyzed to form a number of smaller AMPs such as lactoferricin and lactoferrampin that display similar or even higher antibacterial activity than the full polypeptide (Gruden and Poklar, 2021).

In AD, LTF has been identified as a key predictor of amyloid pathology (Tsatsanis et al., 2021) and also directly affects the amyloidogenic Aβ secretory pathway. For instance, when LTF is bound with iron it stimulates the amyloidogenic processing of APP to form Aβ (Tsatsanis et al., 2021). Conversely, astrocytic LTF overexpression (that would mostly lack iron) has recently been demonstrated to lower both Aβ and Tau hyperphosphorylation in APP/PS1 mice via targeting the LRP1 receptor, leading to downstream PP2A enzyme activity and ultimately reducing Aβ burden (Fan et al., 2024). These opposing effects that are dependent on iron loading status is suggestive of a damage sensing mechanism of LTF that triggers activation of other downstream pathways. LTF can also be synthesized by pro-inflammatory activated microglia (Fillebeen et al., 2001), and may also be related to the iron disturbances in the AD brain with senescent iron-accumulating microglia and lipid-rich microglia (Zeineh et al., 2015; Angelova and Brown, 2019; Marschallinger et al., 2020). Interestingly, LTF is also capable of binding directly to LRP1, TGFβR2, and IGF-R1, triggering downstream metabolic alterations in osteoblasts (Tian et al., 2023). Curiously, this links LTF with lipid handling, anti-inflammatory signaling and insulin signaling. This is relevant as aberrant signaling of all three of these receptor pathways is evident in AD (von Bernhardi et al., 2015; Shinohara et al., 2017; Sędzikowska and Szablewski, 2021). Furthermore, LTF levels are increased in AD patient brains, with large amounts being associated with senile plaques and neurofibrillary tangles. This has been proposed to be an endogenous protection mechanism by microglia or infiltrating monocytes (Li and Guo, 2021). Furthermore, astrocytes also secrete LTF and loss of function in astrocytes leads to cognitive deficiency in young mice due to interference with cholesterol transport (Xu et al., 2022). In this way it would synergize with ApoE due to the shared LRP1 receptor affinity (Fan et al., 2024; Martiskainen et al., 2013). Furthermore, increased astrocytic LTF appears to reduce Aβ burden in APP/PS1 AD mice (Fan et al., 2024).

Evidence from patients indicates reduced salivary LTF specific to AD and no other forms of dementia, a finding that supports the theory that reduced oral antimicrobial defenses and subsequent increased oral pathogen burden are responsible in part for AD pathogenesis (González-Sánchez et al., 2020). It is worth speculating that the microbial burden of AD is a distinguishing factor compared to other types of dementia. Interestingly, ApoE4 carriers have lower LTF levels in their saliva, which would serve as another mechanistic connection between ApoE4 and AD pathogenesis (Carro et al., 2017). It would be beneficial to experimentally determine whether ApoE4 carriers also have lower levels of LTF in their brains as a baseline and during AD.

**Figure 1:**
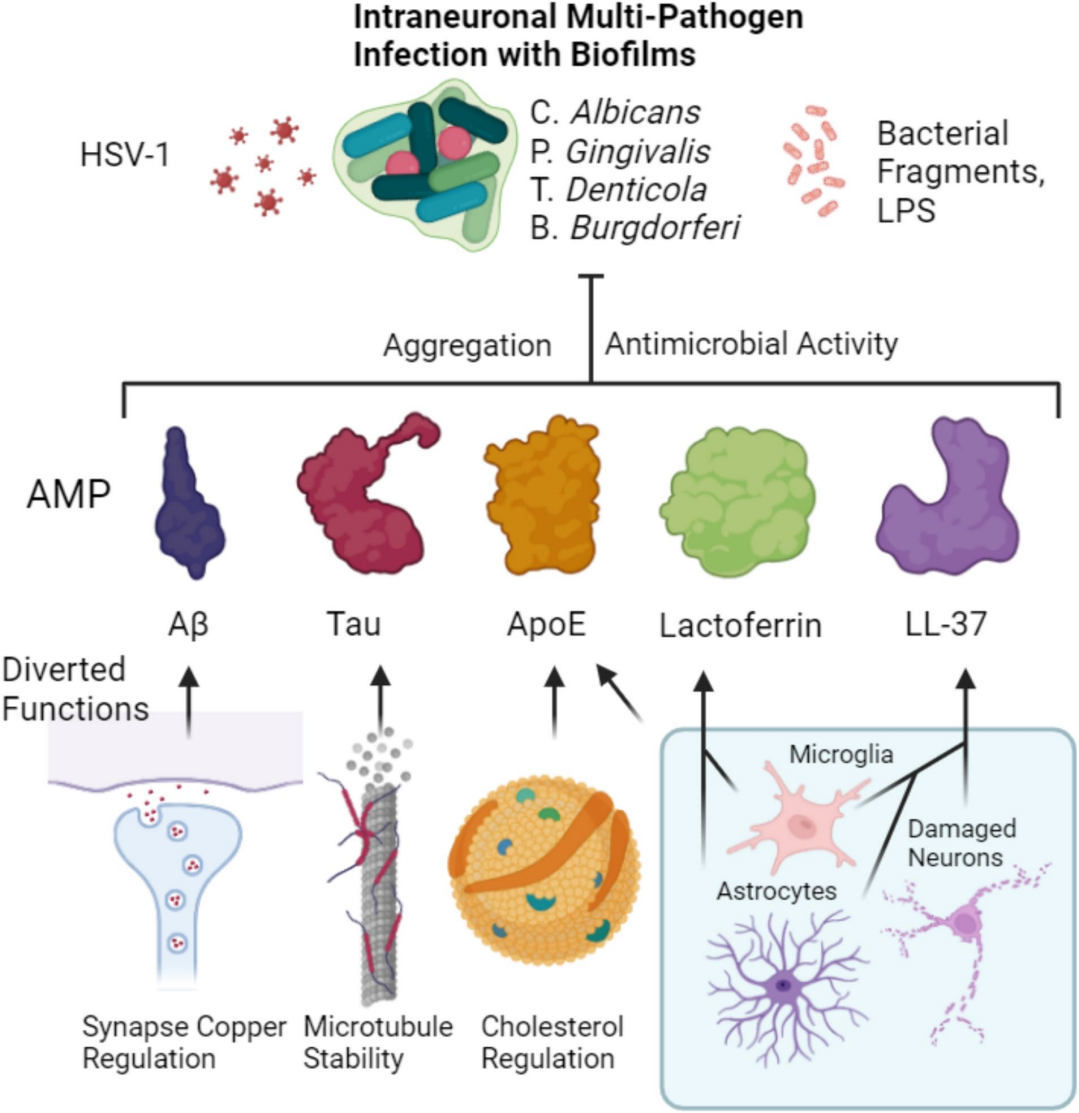
Putative combined antimicrobial effect of major AD related peptides and proteins. Homeostatic functions of Aβ, Tau and ApoE are diverted from their respective roles of synaptic copper regulation, microtubule stability and cholesterol regulation. Instead, they are diverted to an aggregation-based antimicrobial effect that is aimed at intraneuronal biofilm residing microbes as well as viruses or their toxins such as LPS. Microglia and astrocytes both produce lactoferrin which acts similarly as an antimicrobial. Likewise, both microglia and astrocytes can also produce LL-37 (Lee et al., 2015), that can contribute to the defense alongside its release by damaged neurons (Stuart et al., 2022). ApoE can be contributed by microglia increasingly upon inflammation to the downregulation of astocytic ApoE.

Although the clinical implementation of LTF in AD is scarce, one pilot clinical trial of LTF in AD patients resulted in decreased inflammatory and oxidative stress markers with enhanced cognitive function in the LTF treated group after a 3-month period (Mohamed et al., 2019). This could be potentially due to antimicrobial effects but also due to inherent neuroprotective effects of LTF as demonstrated in preclinical models of AD and Parkinson’s disease or both (Yong et al., 2023). LTF therefore appears to be a multifaceted molecule with many positive effects on metabolism, immunity and neurological function that would be of benefit in AD. Its desirable traits of antimicrobial activity and induction of anti-inflammatory pathways which likely synergize with the secondary antimicrobial properties of ApoE, A*β* and Tau would further add to a broad antimicrobial coverage. Given the upstream regulatory effects of LTF on Aβ and Tau, it is tempting to speculate that LTF acts as a signaling molecule with differential effects dependent on iron loading status, that once there is free iron released into the system would increase the antimicrobial response by upregulating the response of other AMPs. This leads to the tempting speculation that there is also tiered system of AMP activity in the CNS, namely LTF and LL-37 acting as primary defense mechanisms, and once they become insufficient in their AMP activity, ApoE, Aβ and Tau could then compensate for this insufficiency. For instance the opposing effects of iron loaded vs. non-loaded LTF upstream of Tau phosphorylation and Aβ stimulation would suggest that a pathogen induced shift to a more iron rich LTF could act as a microbial burden sensor, thus enhancing Aβ secretion and Tau phosphorylation to compensate. A putative scenario of how AMPs act in unison toward an antimicrobial response is illustrated in Figure 1.

## The consequence of prolonged AMP response: copper enzyme disruption and neurodegeneration

So far we have provided evidence and discussion on the role of antimicrobial response of AMPs in AD, but have not discussed how this antimicrobial response may actually lead to the neuronal dysfunction and or death. If AD is caused by microbial infection, then do the microbes kill the neurons or do the neurons die from the consequences of mounting a prolonged antimicrobial response? The exact cause of neuronal cell death in AD has not been fully established but could involve a number of apoptotic, necrotic, pyroptotic, ferropoptotitic and perhaps cuproptotic cell death (Mangalmurti and Lukens, 2022; Goel et al., 2022). It is becoming increasingly clear that there is a massive energetic deficiency in AD brains that is paired with mitochondrial dysfunction, it is possible that such pathologies and loss of function precede the actual neuronal death itself.

It has been well established that Aβ causes mitochondrial impairment, an effect that happens through a variety of mechanisms including oxidative phosphorylation impairment, reactive oxygen species (ROS) and alteration of mitochondrial proteins (Pagani and Eckert, 2011; Mossmann et al., 2014). Early evidence of mitochondrial dysfunction in found in AD patient brain samples indicated the most severe decrease in activity was in cytochrome c oxidase (COX) (Parker et al., 1994), a finding that is also evident in platelet mitochondria (Cardoso et al., 2004). This observation was mechanistically determined to be in part due to Aβ_1–42_ binding directly to COX1 and inducing loss of enzymatic activity of the greater mitochondrial electron transport chain complex 4 (C4), of which COX1 is an integral part. Furthermore, when aggregated, Aβ_1–42_ has an almost doubled binding affinity to copper (and zinc) over the monomeric form that is attributed to conformational changes of Aβ_1–42_ in the greater aggregate structure. This allows for more histidine residues to be exposed for binding than normal, leading to the ability to strip away copper from other copper binding proteins such as albumin (Jiang et al., 2013). This could explain the abnormally accumulation of high levels of zinc, iron and copper found within AD senile plaques (Feng et al., 2023). The increased iron binding could also be working through a similar principle as histidine has a strong affinity for copper, it can also bind zinc and iron to a lesser degree (Vera-Aviles et al., 2018). This metal bound Aβ_1–42_ has been investigated as a cause of ROS as copper and iron bound to these plaques are redox active and can participate in the Fenton reaction to generate ROS (Sasanian et al., 2020). Furthermore, high levels of copper in acidic and or hypoxic environments accelerate Aβ_1–42_ aggregation via primary nucleation and aggregation of other proteins, respectively (Sasanian et al., 2020; Zuily et al., 2022).

Copper’s precise role in the aggregation of Aβ isoforms is highly dependent on experimental parameters (Sasanian et al., 2020; Posadas et al., 2023). However aggregation is likely to be in part due to either primary nucleation in conditions that favor the Cu^1+^ charge state such as hypoxia or acidity, or amorphous non-βsheet aggregates induced by Cu^2+^ (Jin et al., 2011). Structural insights into meningeal amyloid fibrils derived from AD brains reveals polymorphous copper loaded aggregates with the N-terminal histidines 13–14 (responsible for canonical Aβ-copper coordination) resting on the outer structure that surprisingly do not participate in the β-sheet structure, indicating these metal binding regions likely do not participate in the copper-induced aggregation of Aβ. In contrast, *in vitro* metal-free Aβ aggregates form highly ordered fibrils where these histidines 13–14 are embedded within the β-sheet structure. Comparison between these two structures suggests the influence of copper on the dynamics in AD patients (Posadas et al., 2023). We can speculate that the mechanisms of copper induced aggregation of Aβ in patients could be due to Cu^1+^’s proclivity to aggregate cysteine and histidine-rich proteins (Zuily et al., 2022), as it can catalyze oxidation of cysteine containing peptides forming inter-molecular disulfide bridges via inner sphere electron transfer (Prudent and Girault, 2009). There may also be interactions with other amino acids such as tyrosine, methionine, tryptophan and histidine, the only other amino acids that can be oxidized, or in the case of arginine, act as an anchorage site for Cu^1+^ (Prudent and Girault, 2009). These mechanisms could potentially explain Aβ aggregation but they require further investigation, although difficult due to the high sensitivity of Aβ aggregation to experimental conditions such as pH and oxygenation, and the influence of other factors *in vivo* such as Aβ binding to lipids and cell surface gangliosides (Morgado and Garvey, 2015), ApoE (Xia et al., 2024), and potentially bacterial amyloids (Elkins et al., 2024).

In this regard there is a notable interaction of Aβ with ApoE and copper, as copper-Aβ aggregation is enhanced with the addition of all ApoE isoforms, ApoE4 further enhancing copper accelerated Aβ aggregation (Moir et al., 1999). This enhanced aggregation activity of ApoE4 could be due to ApoE4 promoting more locally available copper and more accelerated copper-Aβ complexes (Zhang et al., 2022). ApoE also appears to be a metal ionophore that can sequester copper and reduce oxidative stress, with ApoE4’s antioxidant ability being significantly poorer compared to ApoE3 or ApoE2, presumably due to diminished metal ion binding capacity (Zhang et al., 2022; Miyata and Smith, 1996). In an antimicrobial context, this could tentatively be interpreted as ApoE playing a role in coordinating metal ions to alter Aβ aggregation dynamics toward increased cytotoxic aggregate formation to increase Aβ’s antimicrobial efficacy (either alone or in synergy with other AMPs and copper), for which ApoE4 might exert the strongest synergistic AMP effect due to its copper-Aβ interaction and its ability stabilize cytotoxic Aβ oligomers/fibrils (Garai et al., 2014).

Copper can also oxidize Tau, forming intramolecular disulfide bridges via binding to the C291 and C322 on the R2 and R3 sequences, promoting both Tau phosphorylation and aggregation and also being detected in NFTs (Zubčić et al., 2020). Furthermore, clinical evidence implicates ApoE4’s interaction with Aβ is related to increased Tau deposition in AD vulnerable regions in the brain (Therriault et al., 2021), which mirrors the importance of Aβ in the acceleration of AD Tau dynamics (Lee et al., 2022). ApoE also inhibits the interaction of Tau with LRP1 with ApoE4 being the strongest inhibitor that is suggested to be due enhanced binding affinity to LRP1 (Cooper et al., 2021; Zhang et al., 2022). This impaired clearance would also be compounded by exogenous copper downregulating LRP1 expression (Singh et al., 2013), that could further impede CNS clearance of LRP1 ligands such as Tau or Aβ. The cumulative evidence of the mutual interaction of copper with Aβ, Tau and ApoE, warrants further investigation, especially in terms of antimicrobial potential.

Although speculative, it is possible that Aβ_1–42_ aggregation is enhanced in the acidic lysosomes due to the high concentration of copper required for antimicrobial activity via copper induced ROS formation from lysosomal H_2_O_2_ (Schützmann et al., 2021; White et al., 2009). Evidence to support this theory lies in the demonstration that the endosomal/lysosomal compartment is the key site of Aβ oligomerization in neurons where oligomerization is enhanced 8,000 times in these conditions of low pH compared to the extracellular compartment where they may be released (Schützmann et al., 2021). Interestingly, it appears to be the role of microglia to take up such secreted Aβ_1–42_ oligomers and to assist in the formation of dense plaques that are less toxic than the oligomers themselves (Huang et al., 2021). This could possibly be a trapping mechanism for final agglutination of microbes into a metal ion rich, AMP-encased microbial aggregate. The ability of copper to enhance Aβ_1–42_ oligomerization under low pH conditions raises an interesting possibility that other AMPs may likewise be using copper as a catalyst to facilitate this oligomerization step critical to AMP activity. It is highly interesting to point out the copper binding ability of LL-37 (Makowska et al., 2019), lactoferrin (Harrington et al., 1987), Tau (Di Natale et al., 2018) (for which copper accelerates its phosphorylation; Voss et al., 2014), ApoE (Xu et al., 2014) and other copper binding AMPs not discussed in detail here such as prion protein (Pasupuleti et al., 2009; Salzano et al., 2019), and alpha synuclein (Park et al., 2016; Li et al., 2020). It would therefore be of benefit to establish the role of copper in the oligomerization and or activity of AMPs and to determine if this is an additional factor that contributes to the important role of copper in the immune system (Focarelli et al., 2022; Cheng et al., 2022).

It is interesting therefore to consider the synapse which we have previously discussed, as it is extremely rich in copper and Aβ_1–42_. Here we can envisage a scenario where these elements are diverted from their basic functions to form a unified antimicrobial response resulting in loss of function and a build-up of metal-laden aggregated microbes. In spite of these copper-rich senile plaques found in AD (Bagheri et al., 2018), we see evidence of a more generalized brain-wide copper deficiency that is central to this disease (Xu et al., 2017), that has been postulated to be due to the copper binding ability of Aβ leading to neuronal copper deficiency, a process that is exacerbated by the increased copper requirement in microglia (Bagheri et al., 2022). More investigation is required to see if neuroinflammation facilitates diversion of brain copper from normal neurotransmission and cuproenzyme function toward immune system usage for instance by microglia and by Aβ which will then be later excreted. It is possible that the increase in copper intake by microglia during inflammation similar to in macrophages (Solier et al., 2023).

This also leads to a potential answer to the unsettled discussion around elevated serum levels of copper in AD patients (Bagheri et al., 2018). Aβ may be chelating copper ions away from the CNS via possible combined export across LRP1 (Wang et al., 2021), and or concurrently through direct sequestration of copper in the serum that would exit through urinary excretion, as abnormal Aβ levels and ratios are a common feature AD patients urine (Yang et al., 2021). Aβ could thus be acting as a copper chelating drug. Although there is evidence indicating that AD patients have a higher baseline copper excretion in the urine compared to controls (Squitti et al., 2018), we are unaware of any studies to date that have directly examined Aβ bound together with copper in the blood or urine, a question that would benefit from being answered. Although speculative, it is tempting to consider that the detrimental interaction of Aβ_1–42_ with mitochondria may be related to its evolutionary origin as an enslaved proteobacteria and therefore may experience some off-target effects of AMPs (Roger et al., 2017; Cavalier-Smith, 2006). Evidence to support this theory lies in the recognized negative effects of bactericidal antibiotics on mitochondria (Wang et al., 2015; Kalghatgi et al., 2013), and that like Aβ_1–42,_ LL-37 has been shown to directly bind and damage mitochondria (Bankell et al., 2022), a feature that could be shared with similar AMPs.

Although we have focused on the mitochondria as we believe that this core respiratory deficiency is the most important example of a copper-dependent system as it governs metabolism (Ruiz et al., 2021), it is necessary to point out the key role of other copper-dependent systems that would be compromised in the brain-wide copper deficiency we see in AD. Firstly, there is ceruloplasmin (CP), which is a multi-copper ferroxidase enzyme found around the body including the CNS, whose role is to regulate intracellular iron levels (Liu et al., 2022). CP activity is compromised in AD patient cerebrospinal fluid (CSF) such that despite having similar copper levels, the CP oxidase activity was much lower in AD patients with higher levels of non-functioning, copper-depleted CP (Capo et al., 2008). To date there is little information on the functioning and copper loading of the membrane-bound CP found in cells of the CNS in AD, which would be useful to determine (Ward et al., 2014). Any reduction of copper loaded functional CP would lead to a pathological iron retention due to the necessity of CP to oxidize ferrous iron to ferric iron for export and binding to transferrin (Lopez et al., 2023). One is reminded of the 80% functional loss of CP ferroxidase activity in PD cases and iron accumulation in their substantia nigra (Ayton et al., 2013).

Furthermore, vascularization would be compromised by the dearth of copper available due in part to hypoxia inducible factor 1 (HIF-1) which is dependent on copper to function (Feng et al., 2009). HIF-1 lies upstream of vascular endothelial growth factor (Hu et al., 2016), which itself is directly (or indirectly via HIF-1) induced by copper (Sen et al., 2002). Together this may also explain the vascular defects evident in AD (Mazza et al., 2011). Furthermore, activity of critical neurotransmitter synthesis enzymes such as dopamine beta hydroxylase have been long known to be reduced in AD (Cross et al., 1981), which are also dependent on copper for its catalytic activity and would likewise be compromised by copper deficiency (Vendelboe et al., 2016). Superoxide dismutase 1 (SOD1) is also dependent on copper and likewise would see a decline in activity, supported by a study observing Aβ directly interacting with SOD1 and decreasing enzymatic activity (Yoon et al., 2009). Interestingly hypoxia induced copper import appears conserved in biological systems (Zuily et al., 2022; Zimnicka et al., 2014; Pourvali et al., 2012; White et al., 2009). In bacteria this has been implicated as one reason why copper induced protein aggregation is significantly intensified during hypoxia (Zuily et al., 2022), a possibility that could also apply for mammalian cells. If true, then hypoxia could induce excessive copper influx that pressures cellular copper buffering leading to protein aggregation. Aβ aggregation could thus be enhanced, depriving copper from other systems. This hypoxia induced shunt of copper could end up depleting the surrounding extracellular environment of copper in the brain, forcing it into cells in a form of co-existent copper toxicity and functional deficiency. Although congruent with clinical observations, this requires experimental determination.

**Figure 2:**
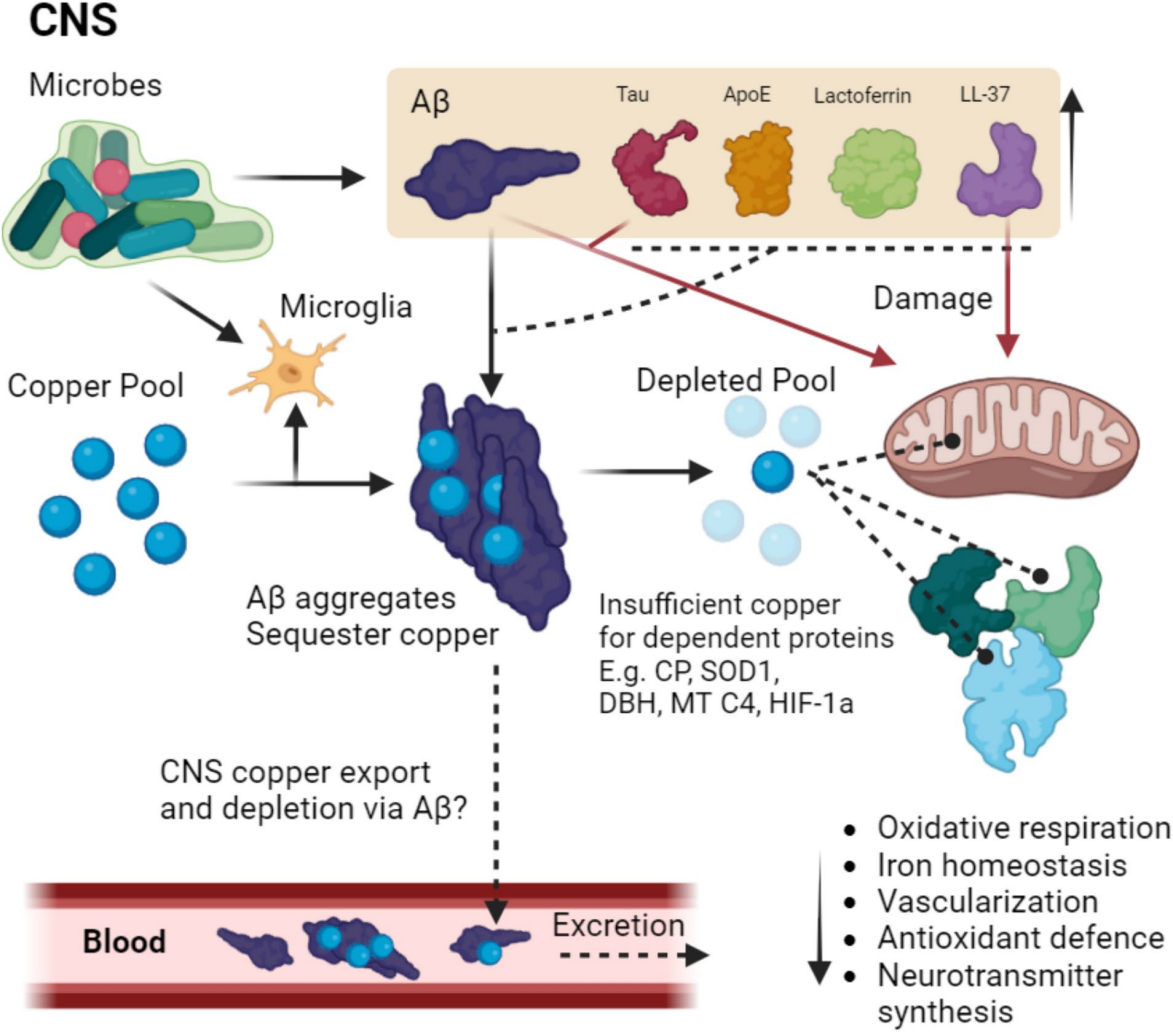
Putative pathway of microbial-induced copper depletion in MAD. Microbes in the CNS trigger the upregulation and aggregation of Aβ which is aggregated thus binding copper, a process that may be enhanced by other copper binding AMPs such as Tau ApoE, LTF, and LL-37. Microbes also trigger microglia to upregulate copper stores for the immune response, further depleting CNS stores. The depleted pool means the inadequate copper loading of copper-dependent proteins such as CP, SOD1, dopamine beta hydroxylase (DBH) mitochondrial complex 4 (MT C4) and HIF-1a, thus leading to disruption in oxidative respiration, iron homeostasis, vascularization, antioxidant defense and neurotransmitter synthesis. Additionally, Aβ Tau and LL-37 damage mitochondria, which is potentially due to the proteobacterial origin of the organelle. Copper could in theory also be further depleted via binding to Aβ as a large portion of Aβ is cleared via the periphery. Dashed lines terminating in a circle indicate improper loading of copper into the target protein, dashed lines terminating in an arrow indicate speculations that require experimental confirmation.

Copper deficiency may in fact be a core feature of neurodegeneration alongside protein aggregation and be the cause of functional deficits in AD. This idea is supported by other neurodegenerative diseases experiencing severe localized copper deficiency, such as the motor neurons of ALS patients (Hilton et al., 2024), PD patients with up to 50% reduction of copper seen in the substantia nigra (Bisaglia and Bubacco, 2020), brain copper reductions in prion diseases (Sorenson, 2001), and notably Menkes disease which is characterized by an X-linked defect in copper transporting ATP7-A gene that leads to severe systemic copper deficiency and progressive neurodegeneration (Tümer and Møller, 2010). Therefore, microbial- induced brain copper deficiency could be a fundamental scenario occurring in AD patients, which if true would greatly influence how we develop future therapies, a topic which we will discuss later. A summary of the hypothesis is illustrated in Figure 2.

## Do microbes cause AD?

It is unlikely to be a coincidence that each of these AMPs, ApoE, Aβ, Tau and LTF are implicated in AD. This combined evidence suggests that they might synergize during AD to protect the CNS against infection. In recent years there has been accumulating evidence that microbes are involved in AD pathogenesis that has been covered in a large number of reviews, many of which call for investigation of antimicrobial therapies (Vigasova et al., 2021; Panza et al., 2019; Song et al., 2021; Catumbela et al., 2023; Bulgart et al., 2020; Piekut et al., 2022). Importantly, the authors point out that the species of microbes found within the CNS of AD patients is not uniform across patients, which suggests common pathologies might be induced by a variety of different microbes often working in synergy. Several recent reviews have provided good coverage of the clinical evidence of pathogens in AD that compile evidence from patient brain, serum and gut samples into a large and comprehensive multi-taxon overview of AD pathogens (Vigasova et al., 2021; Catumbela et al., 2023). Notably, they draw from a large number of studies that together highlight the involvement of a many different species of oral pathogens and lifelong latent viruses that can be detected in the AD brain, that include not only bacteria and viruses, but also bacterial components such as lipopolysaccharide (LPS) and also fungi. Chief among them appears to be the previously mentioned *P. gingivalis*, with one study demonstrating DNA in the CSF and brains of AD patients and then successfully inducing brain infection with *P. gingivalis* following repeated oral infection and concomitant Aβ deposition in the brains of wild type mice (Dominy et al., 2019). Importantly, *P. gingivalis* is known to downregulate host antibacterial responses such as reducing IL-1, IL-6 and IL-8 levels and is capable of infecting and living within cells such as gingival fibroblasts, microglia, astrocytes and neurons (Dominy et al., 2019; How et al., 2016; Liu et al., 2017). In addition, *P. gingivalis* has also been shown to increase BBB permeability (Lei et al., 2023) and is able to form particularly problematic biofilms similar to other gram negative spirochete bacteria that can also provide shelter for yet other microbes (Bao et al., 2014; Allen, 2021), and is been considered a keystone pathogen that can reshape the surrounding microbiota (Hajishengallis et al., 2012).

HSV-1 infection can also promote neuroinflammation by triggering the type 1 interferon response. This type of neuroinflammation is a natural protective response against viral infection, for which the key player in the CNS is microglia (Feng et al., 2023). For innate immune responses to viruses it is important to note that the most common mutation (R47H) in the microglial phagocytic triggering receptor expressed on myeloid cell 2 (TREM2) is a strong risk factor for AD and is characterized by an enhanced type 1 IFN response (Korvatska et al., 2020; Guerreiro et al., 2013). This is perplexing as TREM2 (generally known to be key to Aβ clearance; Zhao et al., 2018), has been implicated as an essential factor in the protective microglial response to herpesvirus infection, with increased phagocytosis of infected neurons (Fruhwürth et al., 2023). One could expect an enhancement of the type 1 IFN response seen in the R47H to be a beneficial mutation if it means a more aggressive containment of the virus, but this could also be interpreted as an overactive immune response that leads to enhanced collateral damage. Repeated activation could ensure a population of inflammation-predisposed microglia vigilant for recurring infection. GWAS studies have indicated the importance of the microglial phagocytic receptors TREM2 and CD33 in the pathogenesis of AD (Griciuc and Tanzi, 2021), and this has been core to the concept of neuroinflammation as a driver of AD. However, while these mutations are not sufficient to cause AD it has been suggested that they exacerbate environmental factors such as viruses and other infections (Blackhurst and Funk, 2023). Given such evidence, the neuroinflammatory theory of AD could be incorporated into MAD.

We also see intracellular biofilms inside hippocampal neurons of AD patients that have been suggested to be from spirochete bacteria of the treponema family (Miklossy, 2015; Allen, 2021). Biofilms have been shown to protect HSV-1 from antivirals and UV laser treatment (Ascione et al., 2017), and biofilms can act as a reservoir of HSV-1 (Mazaheritehrani et al., 2014). Evidence suggests HSV-1 and periodontal pathogens also synergize in part due to the trouble the immune system faces when mounting a contradicting antiviral and antibacterial response against *P. gingivalis* & HSV-1 in the same tissue (De Rodrigues et al., 2015; Arduino et al., 2022), an effect which might be evident in the AD brain. Overall, there are a wide range of microbes that have been implicated in AD pathogenesis with different routes of origin including the oral, gut and lung microbiomes. Table 1 provides a non-exhaustive overview of the major microbes implicated in human AD with supporting evidence from both clinical and preclinical settings.

**Table 1:** Major microbes implicated in human AD.

Microbe	Type	Origin	Clinically detected in	Biofilm involvement	Other notes
*P. gingivalis* (Costa et al., 2021)	Gram^-ve^ bacteria	Oral	AD brains and CSF (Dominy et al., 2019; Ryder, 2020), *P. gingivalis* LPS detected in AD brains (Poole et al., 2013).	Yes, primary colonizer (Bao et al., 2014; Yamada et al., 2005)	Can infect neurons (Haditsch et al., 2020), microglia (Liu et al., 2017), Cause BBB permeability (Lei et al., 2023) Persistent pathogen, cell–cell transmission (Li et al., 2008) Aggravates gut colitis (Jia et al., 2024).
*T. denticiola*	Gram^-ve^ Spirochete bacteria	Oral	AD brains and Trigeminal ganglia (Riviere et al., 2002)	Yes, co-aggregator (Yamada et al., 2005)	
*B. burgdorferi*	Gram^-ve^ Spirochete bacteria	Zoonotic (tick) (Kurokawa et al., 2020)	AD brain and CSF (Riviere et al., 2002; Miklossy, 2011)	Yes (Sapi et al., 2012),	Found in AD brain colocalizing with biofilm and amyloid plaques and p-tau (Senejani et al., 2022).
*C. pnemoniae*	Gram^-ve^ obligate intracellular bacteria	Lung (Balin et al., 2018) Olfactory system (Chacko et al., 2022)	AD brains (Balin et al., 2018)	Can form mixed biofilms with *B. burgdorferi* (Sapi et al., 2019).	Can infect neurons, astrocytes and microglia seen in AD brains (Balin et al., 1998), attachment to microglia and astrocytes enhanced by ApoE4 (Gérard et al., 2008) and higher bacterial loads in AD ApoE4 carrier brains (Gérard et al., 2005). Isolated and cultured from AD brain (Dreses-Werringloer et al., 2009). Persistent infection (Chacko et al., 2022). Induces Amyloid deposits in mice via intranasal infection (Little et al., 2004).
*H. pylori*	Gram^-ve^ bacteria	Gut	Evidence of gut infection and AD risk (Douros et al., 2024; Malaguarnera et al., 2004). Increased anti-*H. pylori*-specific IgG found in CSF (Kountouras et al., 2009).	Can form biofilm (Hathroubi et al., 2018), but lack of evidence of whole bacteria found in brain.	Gut dysbiosis, promotes inflammation and disrupt BBB (Franceschi et al., 2019), predisposes to AD (Contaldi et al., 2017).
*E. coli*	Gram^-ve^ bacteria	Gut	LPS detected AD brain (Zhan et al., 2016)	Yes (Sharma et al., 2016), but lack of evidence of whole bacteria found in brain	
Mixed fungal species including *C. albicans*	Fungi	Oral, Gut, other	AD CSF (Alonso et al., 2015), AD brain (Alonso et al., 2014; Alonso et al., 2018)	Can form mixed biofilms with *P. gingivalis* (Satala et al., 2022)	
HSV-1	Alphaherpesviridae, dsDNA capsid virus (Saleh et al., 2023)	Primarily Oral	AD brain plaques (Wozniak et al., 2009; Readhead et al., 2018)	Can be found trapped and protected in *C. albicans* biofilms (Ascione et al., 2017).	Found in 90% of AD plaques, 72% of the total HSV-1 brain load is found in the plaques compared to only 24% in aged normal brain plaques (Wozniak et al., 2009). Linked to ApoE4 carriers (Linard et al., 2020), Lifelong persistent infection (AlMukdad et al., 2023)

Periodic HSV-1 reactivation might contribute to AD pathogenesis through re-triggering of an inflammatory CNS state (Itzhaki, 2021). This process of recurrent infection has been further explored using model murine cytomegalovirus infection in mice, indicating that repeated infection can induce long term neuroinflammation and cognitive deficits (Harrison et al., 2024). It may be that during periods of host weakness, microbes can opportunistically resurge, especially if protected by biofilms and other persistent mechanisms.

This range of microbes from multiple sources generally limits a monogenic view of the origin of MAD. For instance, despite the evidence for Borrelia species found in AD brain plaques, other studies have failed to find such species in the brain (Gutacker et al., 1998; Wormser et al., 2022), and have thus argued against Borrelia involvement in AD. These types of disagreements arise naturally due to the conflicting evidence of monogenic theories of complex diseases such as AD that may be better situated into an encompassing theory such as in MAD without contradiction as various other microbes could achieve a similar outcome as Borrelia. With regards to postmortem diagnostics in AD, more generalized approaches could be more routinely taken such as metagenomic sequencing to identify all microbes present in the plaques, or else alginate staining to detect biofilms that could be from a variety of sources.

It is therefore interesting to note the positive effects of antimicrobials clinically in AD patients, most notably the broad spectrum antibiotics cycloserine, a combination of doxycycline and rifampin, and *Helicobacter pylori* elimination protocols consisting of omeprazole, clarithromycin and amoxicillin, all of which led to cognitive improvements (Catumbela et al., 2023). Furthermore, the use of antiherpetic drugs has been associated with a reduction in AD incidence with an almost 10-fold reduction in the later incidence of senile dementia (of which AD is a major form), compared to those with no treatment (Itzhaki, 2018; Itzhaki, 2022), a finding that has since been complemented with additional studies across multiple countries (Itzhaki, 2022). Shingles vaccine has also recently been associated with reduced dementia risk (Taquet et al., 2024). Additionally, a Phase 2 pilot trial that involved the use of the anti-herpetic drug acyclovir was established to be safe, feasible and tolerable for early AD, with a slight improvement in the mini mental state examination (MMSE) scores (Weidung et al., 2022). However, care must be taken with antibiotics as taking antibiotics over time can instead increase in AD risk and other adverse effects (Kim et al., 2022).

Although much recent evidence has been put forward to favor the infectious theory of AD, it is still not definitively known whether pathogen exposure is either necessary or sufficient for the pathogenesis of AD and determining causation remains difficult (Bulgart et al., 2020). Koch’s postulates are considered the gold standard in determining the microbial etiology of a disease (Segre, 2013), but we believe this is difficult to fulfill for a disease that may have multiple etiologies. Although the gold standard, it appears that the Koch’s postulates are poorly suited in the determination of a variable multitaxon infection in the modern era for diseases such as AD. Other criteria such as the Bradford Hill criteria should also be considered (Shimonovich et al., 2021), but this would also suffer for determining a single cause of AD due to the heterogeneity of the AD etiology.

**Figure 3:**
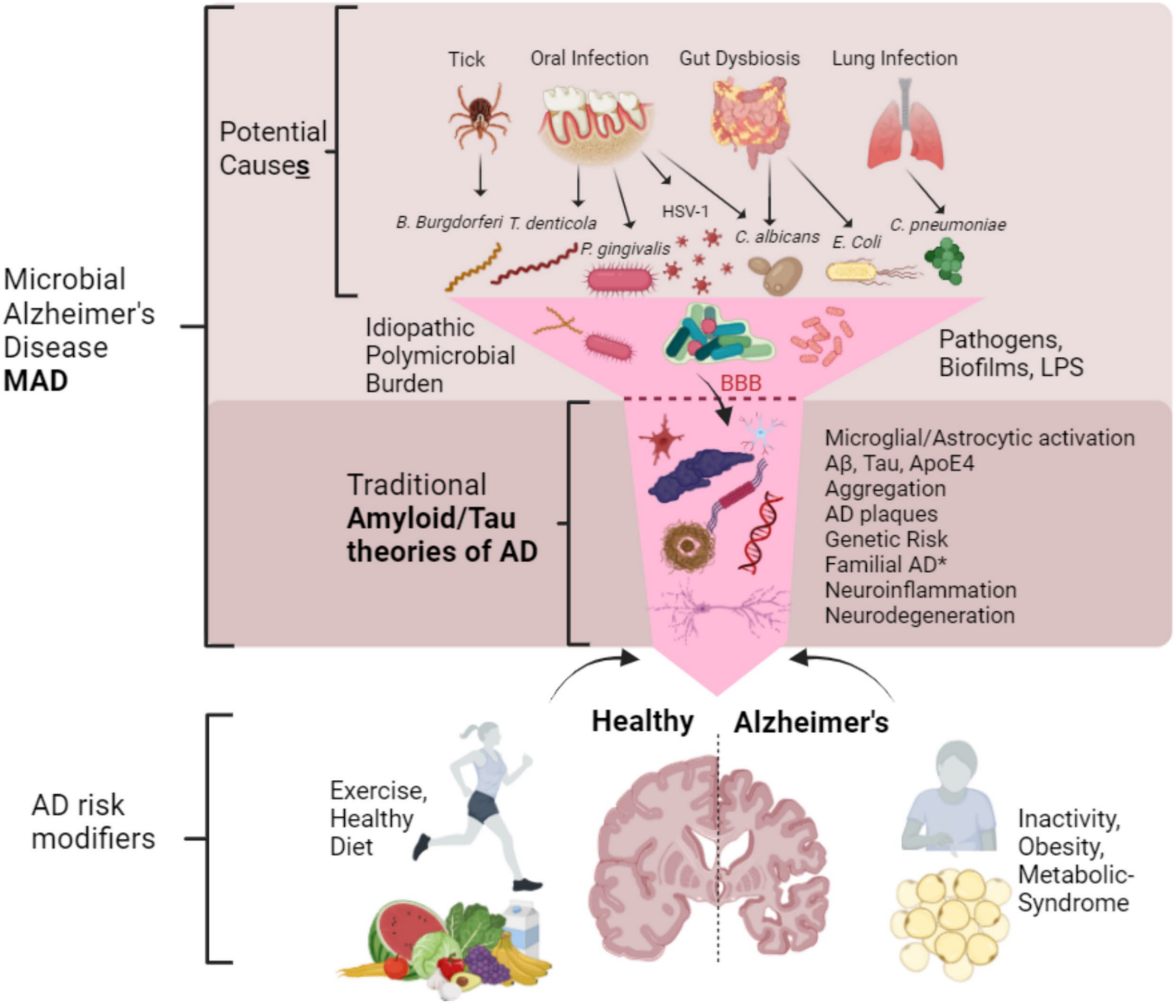
Proposed framework of microbial Alzheimer’s disease (MAD) in relation to other theories of Alzheimer’s disease. MAD is not a different type of AD but arguably a more complete and descriptive name of LOAD. MAD can be caused by many different microbes or their components that originate from the oral microbiota, gut and lung. Together they can act in synergy forming biofilms once they are past the blood brain barrier (BBB) and arrive in the CNS, and their proportions may be idiopathic and heterogeneous. This microbial input funnels down into the traditional Tau and Amyloid theories of AD with the normal set of described pathologies such as protein aggregation and plaque formation. It is of note* that familial AD cases may not necessarily require microbes (although they certainly can contribute) and thus may possibly fit alone into this narrowed portion of common pathologies. AD risk modifiers (not exhaustive) such as good diet and exercise as well as inactivity, obesity and metabolic syndrome may contribute to AD in a synergistic way. Together MAD advances upon the Amyloid/Tau and neuroinflammatory theories by way of integration.

Another issue in explaining MAD is to describe how the bacteria and viruses enter the brain? This has been hypothesized as either due to blood bacteremia from oral pathogens entering the blood via the bleeding gums and thus crossing of the blood brain barrier and/or entry to the brain via the trigeminal nerves (Teixeira et al., 2017; Seaks and Wilcock, 2020), or else through the compromised blood brain barrier (Kivimäki and Walker, 2024), through leaky gut (Köhler et al., 2016) and also the lung (Chen et al., 2023). Demonstrating perfect causality is impossible to fulfill (Vega, 2021), but providing a framework in which the question of AD etiology including MAD and where other theories can be incorporated is proposed in Figure 3.

Despite the clinical evidence for microbial infection in AD, other neurodegenerative diseases such as Parkinson’s disease and ALS also are influenced by microbial infections and induction of inflammatory processes with microglial and astrocytic activation (Lotz et al., 2021), which would question the primacy of microbes in AD. For instance, *H. pylori* infections appear to be associated with chronic inflammation found in AD, PD, multiple sclerosis (MS) and Guillian-Barre syndrome (Álvarez-Arellano, 2014). LPS too appears to be involved in multiple neurodegenerative diseases (Batista et al., 2019), as is HSV-1 (Duarte et al., 2019). Therefore microbial infections are not unique to AD, however the type of microbe associated as a risk factor varies between diseases (Lotz et al., 2021; Kettunen et al., 2024). These diseases appear to be interlinked as they are likely a complex interplay of pathogenic factors. *P. gingivalis* in particular has been suggested to be linked to dementia related aspect of the PD-dementia (Li et al., 2022). Although diseases like ALS have microbial components (Xue et al., 2018), there is strong evidence for the contribution of environmental toxins that are associated the sporadic form of the disease (Newell et al., 2022). Similarly PD is suggested to be strongly related to pesticide exposure as well as trichloroethylene (TCE) exposure (Kieburtz and Dorsey, 2021), with a twin cohort demonstrating an over 500% increase in PD risk when in occupations with TCE exposure (Goldman et al., 2012). In the case of AD, to the best of our knowledge, there appears to be a lack of such evidence that other environmental contaminants are associated with such a strong risk of developing AD, although they are certainly contributory (Vasefi et al., 2020; Elonheimo et al., 2021). On the other hand, there are a dearth of studies that find microbes directly in the affected regions of the brain and spinal cord in PD and ALS respectively, in contrast to the numerous studies mentioned earlier in the case of AD, which is further supported by the direct ability to culture these microbes in some cases. In order to test MAD, we should strive to establish the necessity of a long-term CNS infection of specific areas of the brain with different microbes and observe the outcomes. Furthermore, studies could be conducted to ascertain the rates of infection of CNS in other neurodegenerative diseases such as ALS or PD in a concentration and spatial manner in order to establish the relative pressure of microbes in the CNS for that could both explain the shared microbial burden in these diseases yet adequately distinguish them from each other.

Other related diseases such as fontal temporal dementia (FTD) appears to have overlap both with AD and ALS, and is commonly related with TDP-43 and C9ORF72 mutations of ALS (Warren et al., 2013), although there is evidence for TDP-43 involvement in AD, it appears to take a different progression pattern compared to ALS and FTD (Meneses et al., 2021), which suggests distinct disease mechanisms at play. There also appears to be a lack of evidence of microbial involvement in FTD only cases, such as microbe rich plaques which could further distinguish MAD from FTD. Furthermore these diseases are often comorbid with up to 25% of AD patients with FTD and FTD patients that do not have a comorbid diagnosis with AD have a significantly lower fraction of Aβ than those who do (Tan et al., 2017). If studies looking at FTD could investigate the occurrence of microbes om FTD only patients’ (without comorbid AD) brain regions and determine the existence or lack thereof of key AD related microbes, it could help better define the difference between these related dementias.

Aside from questions regarding the uniqueness of microbes of AD, the observation that AD patients have a poorer oral hygiene that is in part considered due to their declining functional capacity (Gao et al., 2020), suggest that the microbes found in their brains areas as a result of deteriorating mental health and not the cause. This is a legitimate observation, however multiple animal studies in wild type mice show initial infection can lead to many of the hallmarks of AD (Wu et al., 2022; Ding et al., 2018; Ilievski et al., 2018), which suggests primacy of microbial infection that could lead to a feedback cycle of poorer hygiene and increased burden.

If clinical cases of LOAD can be determined to be absent of microbes or their products in the affected brain regions despite extensive testing, this would be good evidence against the MAD theory. However such evidence is not forthcoming. On the other hand, evidence for the MAD theory would be supported if the vast majority AD brain sample are found to contain microbes or their products in the affected brain regions. However, this alone would support correlation and not causation and is a shortcoming of solely examining AD patient brain samples as evidence. Additionally, since AD patient brain samples are collected post-mortem, then contamination and tissue degradation could confound results (Clement et al., 2016). Furthermore, patient information and sample handling procedures should be as detailed as possible as concomitant disease, systemic inflammation, infections and medication intake as well as post-mortem tissue preparation/fixation and storage protocols could affect results and subsequent interpretation of findings (Gomez-Nicola and Boche, 2015). Development of co-infectious models of AD in non-human primates would help clarify findings from clinical samples, although such investigations would only demonstrate the causality of microbes in MAD to the extent of the microbes tested. The variety of microbes in AD patients could be highly idiopathic and from the evidence so far are broad ranging. Attempting to recapitulate this *in vivo* to determine microbial causality would be a challenging but worthwhile investigation.

Further related studies examining EOAD patients to determine if they are protected from acute brain infections and sepsis (similar to 5xFAD mice that are better protected from brain salmonella infection; Kumar et al., 2016), could help in understanding if such mutations are purely deleterious as commonly thought, or if they confer acute survival benefits despite long-term negative consequences, thus supporting MAD from a different angle. Studies that further investigate the suppression of CNS entry of microbes or microbial products in humans would be expected to reduce Tau and Aβ pathologies in accordance with MAD. Furthermore, antimicrobial therapies or therapies aimed at balancing biota and thus reducing the pathogen exposure would be expected to prevent or halt AD, if MAD theory is correct. Otherwise if such studies consistently provide negative results then a falsification threshold of MAD could be argued, similar to the Tau and amyloid casual theories (Espay et al., 2023). The amyloid cascade hypothesis posits Aβ to be the central driver of AD either by abnormal production or hindered clearance, resulting in aggregate stress and neuronal dysfunction/death in a sequential manner (Karran et al., 2011; Doig, 2018), but offers little on the actual origin of LOAD or the driver of abnormal Aβ production and is thus incomplete. In this regard, MAD fundamentally contrasts with the amyloid cascade hypothesis in the notion that Aβ is the central driver of AD, but incorporates it in a more holistic perspective with microbes instead as the central driver.

Still, we should not disregard other AD models and their underlying theories. For instance, beside the genetic models there are 21 additional models of AD tested *in vivo* that range from metal ion toxicity models such as aluminum-based and copper sulfate/cholesterol-based methods, immunological methods such as the IgG-saporin model, and neurotoxin models using colchicine or scopolamine. While each of these models has been useful in recapitulating certain facets of AD, just as with the genetic models they have generally fallen short of accurately recapitulating human disease (Rapaka et al., 2022). Certain environmental factors such as aluminum exposure (that can aggregate Aβ) (Kawahara and Kato-Negishi, 2011), and raw copper exposure in drinking water (that can exacerbate AD by suppression of LRP1) (Brewer, 2014; Hsu et al., 2019), may not necessarily compete with MAD, but instead could be also integrated through common pathways.

It is worth briefly mentioning Down’s syndrome (DS) and AD due to the very high incidence of AD among these patients (Gomez et al., 2020). Recently, a hypothesis has been proposed that may also implicate pathogens in the development of Down’s syndrome (DS) and related Down’s syndrome Alzheimer’s disease (DSAD) (Norins, 2022). This theory appears to be a more modern advancement that is founded on an earlier theory from 1968 based on clinical evidence that demonstrates a correlation of viral infection near conception and development of Down’s syndrome (Kogon et al., 1968). If true, then antiviral or antimicrobial therapies implemented at different stages of disease would provide promise in the prevention of DS and also attenuation of DSAD. We believe that this concept would benefit from being experimentally examined as it raises the possibility that many diseases thought to be well understood may in fact have an insidious microbial etiology.

## Conclusion

Throughout this review we have discussed the major biological functions of the key AD-related peptides and proteins and highlighted their important secondary (or primary) functions as AMPs. In particular we have outlined the importance of synaptic copper regulation by Aβ species, the importance of Tau in neuronal MT and nucleus stability and the lipid modulation of ApoE under physiological conditions, and how all of these can perform additional functions as AMPs which can be best described as protein or peptide moonlighting (Espinosa-Cantú et al., 2020). Together these molecules can synergize with other multifunctional immunological proteins such as LTF in a form of a redundant antimicrobial response that is akin to cytokine redundancy (Ozaki and Leonard, 2002). Since Aβ, Tau, ApoE, and LL-37 can be synthesized by neurons (Lee et al., 2015; Dekroon and Armati, 2001), they represent an array of oligmerization-based antimicrobial tools that can be (re-)appropriated to confer a broad defense against multitaxonomic infections in non-immune specialized cells. The examination of clinical evidence both in the form of post-mortem tissues of AD patients, as well as the partial efficacy of clinical trials that investigate antimicrobial drugs, indicate that microbial infection likely plays a role in the pathogenesis of LOAD. We examine this evidence together that strongly suggests microbes as a driving force in LOAD, but short of self-induction of AD to prove causation (as done so for *Helicobacter pylori*; Marshall and Adams, 2008), we cannot say definitively that microbes cause LOAD. In addition, we recognize the confused nature of causation for complex multifactorial diseases as outlined in Figure 3. MAD appears to be more encompassing and explanatory than the stand-alone Aβ or Tau hypotheses as it bypasses the debate between them entirely (Kametani and Hasegawa, 2018), incorporating them as legitimate arms of a unified response. In the meantime the MAD theory also connects the various clinical observations we have discussed to the molecular mechanisms in a coherent manner and that without much difficulty or contradiction metabolic and metal ion-based theories for LOAD (de la Monte and Wands, 2008; Chen et al., 2023). In addition since there is evidence that even the FAD patients who carry the PSEN1 mutation have signs of viral infection that affect the hippocampus through the olfactory bulb (Bubak et al., 2023), which suggests treatments for MAD would have relevance also with EOAD.

Preclinical genetic models such as those that rely on the overexpression of Aβ (Sasaguri et al., 2017) and their incomplete cognitive manifestations should be better understood to be synthetically modeling the downstream effects of one or more arms of an antimicrobial response. Given the complex moonlighting nature of Aβ, passive immunotherapies aimed at diminishing Aβ in the brain would appear to be at best a partial response to treating AD, that in light of a new meta-analysis appears to actually be associated with accelerated loss of brain volume (Alves et al., 2023), a disturbing finding that confirms previously voiced concerns over this approach (Høilund-Carlsen and Alavi, 2021; Høilund-Carlsen et al., 2023). Furthermore, if MAD is correct then AD could potentially be reconsidered in part communicable which would challenge the existing notion (Bano et al., 2023).

Future investigations based on the MAD theory should take care when taking approaches to target only single microbes such as HSV-1 or else using a broad-spectrum antibiotic without an anti-biofilm measure, as such strategies would likely be only partially productive. However, taking multiple antibiotics and antiviral and anti-biofilm agents such as Diclofenac together could run the risk of compounded harm to the patient, as these drugs often have a wide range of side-effects and could be contraindicated (Mohsen et al., 2020; Eljaaly et al., 2019; Brandariz-Nuñez et al., 2021; Hunter et al., 2011; Ferrer-Luque et al., 2023). There is a case for use of non-steroidal anti-inflammatory drugs for AD as many of these have anti-biofilm and antimicrobial effects, although the clinical data is conflicting (Rivers-Auty et al., 2020). Particularly appealing are antiviral, antibacterial, and anti-inflammatory treatments that can penetrate the BBB and that have low toxicity profiles and selectivity against pathogenic microbes. As discussed, LTF is one such endogenously produced molecule (Mohamed et al., 2019; Kowalczyk et al., 2022; Naidu et al., 2023), and due to its selective antimicrobial effects, favors the predominance of beneficial microbes (Pino et al., 2017). This could be supplemented with attempts to improve restore external pathogen burden in the oral, digestive and respiratory systems. Other AMPs could also potentially be investigated, for instance LL-37 due to its anti-biofilm and antimicrobial properties (Ridyard and Overhage, 2021), histatins for their high biocompatibility and antifungal capabilities (Khurshid et al., 2017), ApoE (Yamazaki et al., 2016), enteric AMPs (Sivieri et al., 2017) or antimicrobial neuropeptides (El Karim et al., 2008). These AMPs could be combined to complement their natural operation within our organism as a layered broad spectrum antimicrobial system.

The main issue with AMPs appears to be a risk to critical copper-based systems, as they impact on many key functions such as aerobic respiration, iron homeostasis, neurotransmitter synthesis and receptor modulation and vascular function that are key to AD. Depletion of copper by Aβ aggregate sequestration could reduce the function of cuproenzyme systems for which we see widespread dysfunction in AD. We argue that it is the prolonged AMP response that diverts many proteins and peptides along with copper to mounting an immune response rather than a homeostatic function. Given the arguable similarities between functional copper deficiencies evident in other neurodegenerative diseases, it is plausible that copper depletion is central node of neurodegeneration in general, only with different chemical/biological induction specific to each disease.

General dysfunction would precede death, and it is loss of vital cuproprotein-based function in still living neurons that we argue is also key to AD. This would be reminiscent of what is occurring in ALS patient spinal cord motor neurons with loss of cuproprotein function and thus overall function that precedes the eventual death of the neurons themselves (Hilton et al., 2024; Iwai et al., 2016), followed by a large decline in brain volume (Traini et al., 2020), associated with long-term progressive decline (Goel et al., 2022). Evidence suggests that higher brain copper levels are associated with slower cognitive decline in AD patients (Agarwal et al., 2022). Historically the use of copper ionophores such as PBT2 have failed to produce efficacious results in clinical trials (Summers et al., 2022), but crucially they have been investigated without the addition of copper, probably due to the theory that it was primarily to disaggregate copper loaded plaques, in which case PBT2 would act as a chelator (Drew, 2017). Delivery of copper using BBB permeable delivery agents such as diacetyl-bis (N4-methylthiosemicarbazone) (CuATSM) used to restore copper deficiency in ALS (Hilton et al., 2024), or administration of the endogenously produced neurotrophic copper binding tripeptide Glycyl-L-Histidyl-L-Lysine -copper (GHK-Cu) (Pickart et al., 2017), or else adding PBT2-Cu would be of interest for investigation.

Another potential candidate would be the use of oxygenation/oxidizing agents such as chlorine dioxide (ClO_2_), a gas that is readily dissolved in water and derived from its prodrug sodium chlorite (NaClO_2_) (Andrés et al., 2022), that has been examined for their ability to penetrate biofilms and to target pathogenic microbes (Behnke and Camper, 2012). For instance, chlorine dioxide has been used to disinfect HIV-infected blood without harming leukocytes (Carmen and Chong, 1993; Kross and Scheer, 1991), and the ClO_2_ prodrug NaClO_2_ has also been approved as an ALS orphan drug by the European medicines agency (EMA) (EU/3/13/1139, 2024), and has demonstrated efficacy in a subset of ALS patients administered NaClO_2_ intravenously in a Phase 2B clinical trial, notably reducing LPS levels in the blood which would suggest antimicrobial efficacy (Zhang et al., 2022). Furthermore, it putatively reacts with the amino acid taurine via mimicry of the macrophage respiratory burst that produces hypochlorous acid to kill microbes and by extension produces the strong anti-inflammatory molecule taurine chloramine (Zhang et al., 2022; Marcinkiewicz et al., 2006). The combined effect would suggest a simultaneous antimicrobial and anti-inflammatory response that can penetrate biofilms and thus could also be a potential candidate for MAD. Although NaClO_2_ has also been patented for use in neurodegenerative diseases (Norviel and McGrath, 2024) and for HIV infections (PubChem, 2024), ClO_2_ formulations could potentially be used instead. However to avoid safety issues, dosage should be taken within safety thresholds (Lubbers et al., 1982). Such a candidate treatment may constitute part of an LPS reduction therapy that could provide evidence to support related theories such as the endotoxin theory of AD (Brown and Heneka, 2024).

Ultimately, these approaches could be employed together. For instance, one could imagine the possible efficacy of a combination therapy of LTF with GHK-Cu or CuATSM combined with methods of reducing oral pathogen load. These suggested approaches could be further combined with changes in diet and lifestyle associated with better AD outcome (Arab and Sabbagh, 2010), in a type of modern combinatorial medicine reminiscent of traditional medicine formulae (Yuan et al., 2016). Other AD risk factors such as type 2 diabetes, obesity and dietary deficiencies could be further integrated into MAD (Armstrong and Risk, 2019), as while such risk factors alone do not cause AD they could enhance AD. We also acknowledge that MAD itself may be proximal to other risk factors, especially diet, since the diet can greatly affect the oral and gut microbiomes, metabolic state and infection susceptibility (Santonocito et al., 2022; Zhang, 2022; Chávez-Reyes et al., 2021). In this regard, close attention should be paid to better understanding of clinical cases of AD reversal achieved in complex targeted interventions that include a mix of sleep, exercise and diet optimization, copper/zinc balancing, heavy metal chelation, hormone balancing, GI tract repair and oral hygiene improvement (Bredesen, 2014; Bredesen et al., 2016; Rao et al., 2023). Such interventions could also potentially be supplemented with antimicrobials, opening up a wide range of potential therapeutic options. It is important to carefully determine the types of antimicrobials to be used as many antibiotics and antivirals can have negative adverse reactions (Mohsen et al., 2020; Poole and James, 2018), and are thus not an option for certain patients (Blumenthal et al., 2019), be functionally biounavailable and in the case of AMPs, risk of increasing aggregation. Furthermore, Drug-resistance could also become a problem and should be carefully monitored.

The MAD theory integrates the Tau and Amyloid theories with the pathogen-based theories such as the endotoxin theory of AD (Brown and Heneka, 2024) and viral AD (Itzhaki, 2021; Naughton et al., 2020), and also takes into account the possible downstream mechanisms such as cuproenzyme dysfunction which we believe is highly important to include for a more complete picture of AD. MAD could best be considered as a theory that encompasses the common triggered response of these pathogens (including their products) with the consequence of a costly unified antimicrobial response that cannot be sustained indefinitely in the CNS without detrimental consequences. The resultant chronic situation could account for the majority of LOAD, ironically returning almost full circle to the early days of Fischer and Bonfiglio’s AD research (Allnutt and Jacobson, 2020; Miklossy, 2015).

## References

[ref1] Abasi L. S. Elathram N. Movva M. Deep A. Corbett K. D. Debelouchina G. T. (2024). Phosphorylation regulates tau’s phase separation behavior and interactions with chromatin. Commun. Biol. 7, 1–17. doi: 10.1038/s42003-024-05920-4 38429335 PMC10907630

[ref2] Agarwal P. Ayton S. Agrawal S. Dhana K. Bennett D. A. Barnes L. L.. (2022). Brain copper may protect from cognitive decline and Alzheimer’s disease pathology: a community-based study. Mol. Psychiatry 27, 4307–4313. doi: 10.1038/s41380-022-01802-5, PMID: 36195639 PMC9764421

[ref3] Alam M. M. Yang D. Li X.-Q. Liu J. Back T. C. Trivett A.. (2022). Alpha synuclein, the culprit in Parkinson disease, is required for normal immune function. Cell Rep. 38:110090. doi: 10.1016/j.celrep.2021.110090, PMID: 35021075 PMC10258816

[ref4] Alhazmi H. A. Albratty M. (2022). An update on the novel and approved drugs for Alzheimer disease. Saudi Pharm J. 30, 1755–1764. doi: 10.1016/j.jsps.2022.10.004, PMID: 36601504 PMC9805975

[ref5] Allen H. B. (2021). A novel approach to the treatment and prevention of Alzheimer’s disease based on the pathology and microbiology. J. Alzheimers Dis. 84, 61–67. doi: 10.3233/JAD-210429, PMID: 34542071 PMC8609710

[ref6] Allnutt M. A. Jacobson S. (2020). Do herpesviruses play a role in Alzheimer’s disease pathogenesis? Drug. Discov. Today Dis. Models 32, 21–26. doi: 10.1016/j.ddmod.2019.10.006

[ref7] AlMukdad S. Harfouche M. Farooqui U. S. Aldos L. Abu-Raddad L. J. (2023). Epidemiology of herpes simplex virus type 1 and genital herpes in Australia and New Zealand: systematic review, meta-analyses and meta-regressions. Epidemiol. Infect. 151:e33. doi: 10.1017/S0950268823000183 36750224 PMC9990408

[ref8] Alonso R. Pisa D. Fernández-Fernández A. M. Carrasco L. (2018). Infection of fungi and bacteria in brain tissue from elderly persons and patients with Alzheimer’s disease. Front. Aging Neurosci. 10:159. doi: 10.3389/fnagi.2018.00159 29881346 PMC5976758

[ref9] Alonso R. Pisa D. Marina A. I. Morato E. Rábano A. Carrasco L. (2014). Fungal infection in patients with Alzheimer’s disease. J. Alzheimers Dis. 41, 301–311. doi: 10.3233/JAD-132681, PMID: 24614898

[ref10] Alonso R. Pisa D. Rábano A. Rodal I. Carrasco L. (2015). Cerebrospinal fluid from Alzheimer’s disease patients contains fungal proteins and DNA. J. Alzheimers Dis. 47, 873–876. doi: 10.3233/JAD-150382, PMID: 26401766

[ref11] Álvarez-Arellano L. (2014). *Helicobacter pylori* and neurological diseases: married by the laws of inflammation. World J. Gastrointest. Pathophysiol. 5, 400–404. doi: 10.4291/wjgp.v5.i4.400, PMID: 25400983 PMC4231504

[ref12] Alves F. Kalinowski P. Ayton S. (2023). Accelerated brain volume loss caused by anti–β-amyloid drugs. Neurol. Int. 100:e2114. doi: 10.1212/wnl.0000000000207156, PMID: 36973044 PMC10186239

[ref13] Ando Y. Nakamura M. Kai H. Katsuragi S. Terazaki H. Nozawa T.. (2002). A novel localized amyloidosis associated with lactoferrin in the cornea. Lab. Investig. 82, 757–765. doi: 10.1097/01.LAB.0000017170.26718.89, PMID: 12065686

[ref14] Andrés C. M. C. De La L. J. M. P. Andrés Juan C. Plou F. J. Pérez-Lebeña E. (2022). Chlorine dioxide: friend or foe for cell biomolecules? A chemical approach. Int. J. Mol. Sci. 23:15660. doi: 10.3390/ijms232415660, PMID: 36555303 PMC9779649

[ref15] Angelova D. M. Brown D. R. (2019). Microglia and the aging brain: are senescent microglia the key to neurodegeneration? J. Neurochem. 151, 676–688. doi: 10.1111/jnc.14860 31478208

[ref16] Arab L. Sabbagh M. N. (2010). Are certain lifestyle habits associated with lower Alzheimer’s disease risk? J. Alzheimers Dis. 20, 785–794. doi: 10.3233/JAD-2010-091573, PMID: 20182018 PMC3207358

[ref17] Arduino P. G. Cabras M. Lodi G. Petti S. (2022). Herpes simplex virus type 1 in subgingival plaque and periodontal diseases. Meta-analysis of observational studies. J. Periodontal Res. 57, 256–268. doi: 10.1111/jre.12968, PMID: 34978079

[ref18] Arendt T. Stieler J. T. Holzer M. (2016). Tau and tauopathies. Brain Res. Bull. 126, 238–292. doi: 10.1016/j.brainresbull.2016.08.018 27615390

[ref19] Armstrong A. Risk R. (2019). Factors for Alzheimer’s disease. Folia Neuropathol. 57, 87–105. doi: 10.5114/fn.2019.85929 31556570

[ref20] Ascione C. Sala A. Mazaheri-Tehrani E. Paulone S. Palmieri B. Blasi E.. (2017). Herpes simplex virus-1 entrapped in *Candida albicans* biofilm displays decreased sensitivity to antivirals and UVA1 laser treatment. Ann. Clin. Microbiol. Antimicrob. 16:72. doi: 10.1186/s12941-017-0246-5 29137671 PMC5686830

[ref21] Avila J. Lucas J. J. Pérez M. Hernández F. (2004). Role of tau protein in both physiological and pathological conditions. Physiol. Rev. 84, 361–384. doi: 10.1152/physrev.00024.2003 15044677

[ref22] Ayton S. Lei P. Duce J. A. Wong B. X. W. Sedjahtera A. Adlard P. A.. (2013). Ceruloplasmin dysfunction and therapeutic potential for Parkinson disease. Ann. Neurol. 73, 554–559. doi: 10.1002/ana.23817, PMID: 23424051

[ref23] Azuma M. Kojimab T. Yokoyama I. Tajiri H. Yoshikawa K. Saga S.. (2000). A synthetic peptide of human apoprotein E with antibacterial activity. Peptides 21, 327–330. doi: 10.1016/S0196-9781(00)00165-0, PMID: 10793212

[ref24] Bagheri S. Saboury A. A. Haertlé T. Rongioletti M. Saso L. (2022). Probable reasons for neuron copper deficiency in the brain of patients with Alzheimer’s disease: the complex role of amyloid. Inorganics 10:6. doi: 10.3390/inorganics10010006

[ref25] Bagheri S. Squitti R. Haertlé T. Siotto M. Saboury A. A. (2018). Role of copper in the onset of Alzheimer’s disease compared to other metals. Front. Aging Neurosci. 9:446. doi: 10.3389/fnagi.2017.00446 29472855 PMC5810277

[ref26] Baitsch D. Bock H. H. Engel T. Telgmann R. Müller-Tidow C. Varga G.. (2011). Apolipoprotein E induces antiinflammatory phenotype in macrophages. Arterioscler. Thromb. Vasc. Biol. 31, 1160–1168. doi: 10.1161/ATVBAHA.111.222745 21350196 PMC3529398

[ref27] Balin B. J. Gérard H. C. Arking E. J. Appelt D. M. Branigan P. J. Abrams J. T.. (1998). Identification and localization of *Chlamydia pneumoniae* in the Alzheimer’s brain. Med. Microbiol. Immunol. 187, 23–42. doi: 10.1007/s004300050071, PMID: 9749980

[ref28] Balin B. J. Hammond C. J. Little C. S. Hingley S. T. Al-Atrache Z. Appelt D. M.. (2018). *Chlamydia pneumoniae*: An etiologic agent for late-onset dementia. Front. Aging Neurosci. 10:302. doi: 10.3389/fnagi.2018.00302 30356749 PMC6189393

[ref29] Bankell E. Liu X. Lundqvist M. Svensson D. Swärd K. Sparr E.. (2022). The antimicrobial peptide LL-37 triggers release of apoptosis-inducing factor and shows direct effects on mitochondria. Biochem. Biophys. Rep. 29:101192. doi: 10.1016/j.bbrep.2021.101192, PMID: 34988298 PMC8695256

[ref30] Bano D. Ehninger D. Bagetta G. (2023). Decoding metabolic signatures in Alzheimer’s disease: a mitochondrial perspective. Cell Death Discov. 9, 1–4. doi: 10.1038/s41420-023-01732-3 38040687 PMC10692234

[ref31] Bao K. Belibasakis G. N. Thurnheer T. Aduse-Opoku J. Curtis M. A. Bostanci N. (2014). Role of *Porphyromonas gingivalis* gingipains in multi-species biofilm formation. BMC Microbiol. 14:258. doi: 10.1186/s12866-014-0258-7, PMID: 25270662 PMC4189655

[ref32] Barbier P. Zejneli O. Martinho M. Lasorsa A. Belle V. Smet-Nocca C.. (2019). Role of tau as a microtubule-associated protein: structural and functional aspects. Front. Aging Neurosci. 11:204. doi: 10.3389/fnagi.2019.00204, PMID: 31447664 PMC6692637

[ref33] Bateman R. J. Aisen P. S. De Strooper B. Fox N. C. Lemere C. A. Ringman J. M.. (2010). Autosomal-dominant Alzheimer’s disease: a review and proposal for the prevention of Alzheimer’s disease. Alzheimers Res. Ther. 3:1. doi: 10.1186/alzrt59, PMID: 21211070 PMC3109410

[ref34] Batista C. R. A. Gomes G. F. Candelario-Jalil E. Fiebich B. L. de Oliveira A. C. P. (2019). Lipopolysaccharide-induced neuroinflammation as a bridge to understand neurodegeneration. Int. J. Mol. Sci. 20:2293. doi: 10.3390/ijms20092293, PMID: 31075861 PMC6539529

[ref35] Beatman E. L. Massey A. Shives K. D. Burrack K. S. Chamanian M. Morrison T. E.. (2016). Alpha-synuclein expression restricts RNA viral infections in the brain. J. Virol. 90, 2767–2782. doi: 10.1128/JVI.02949-15, PMID: 26719256 PMC4810656

[ref36] Behnke S. Camper A. K. (2012). Chlorine dioxide disinfection of single and dual species biofilms, detached biofilm and planktonic cells. Biofouling 28, 635–647. doi: 10.1080/08927014.2012.700705, PMID: 22738417

[ref37] Bennett R. E. DeVos S. L. Dujardin S. Corjuc B. Gor R. Gonzalez J.. (2017). Enhanced tau aggregation in the presence of amyloid β. Am. J. Pathol. 187, 1601–1612. doi: 10.1016/j.ajpath.2017.03.011, PMID: 28500862 PMC5500829

[ref38] Berthon B. S. Williams L. M. Williams E. J. Wood L. G. (2022). Effect of lactoferrin supplementation on inflammation, immune function, and prevention of respiratory tract infections in humans: a systematic review and meta-analysis. Adv. Nutr. 13, 1799–1819. doi: 10.1093/advances/nmac047, PMID: 35481594 PMC9526865

[ref39] Bisaglia M. Bubacco L. (2020). Copper ions and Parkinson’s disease: why is homeostasis so relevant? Biomol. Ther. 10:195. doi: 10.3390/biom10020195, PMID: 32013126 PMC7072482

[ref40] Blackhurst B. M. Funk K. E. (2023). Viral pathogens increase risk of neurodegenerative disease. Nat. Rev. Neurol. 19, 259–260. doi: 10.1038/s41582-023-00790-6, PMID: 36864169 PMC9980857

[ref41] Blades B. Hung Y. H. Belaidi A. A. Volitakis I. Schultz A. G. Cater M. A.. (2024). Impaired cellular copper regulation in the presence of ApoE4. J. Neurochem. doi: 10.1111/jnc.16198, PMID: 39135362

[ref42] Blanchard J. W. Akay L. A. Davila-Velderrain J. von Maydell D. Mathys H. Davidson S. M.. (2022). APOE4 impairs myelination via cholesterol dysregulation in oligodendrocytes. Nature 611, 769–779. doi: 10.1038/s41586-022-05439-w, PMID: 36385529 PMC9870060

[ref43] Blumenfeld J. Yip O. Kim M. J. Huang Y. (2024). Cell type-specific roles of APOE4 in Alzheimer disease. Nat. Rev. Neurosci. 25, 91–110. doi: 10.1038/s41583-023-00776-9, PMID: 38191720 PMC11073858

[ref44] Blumenthal K. G. Peter J. G. Trubiano J. A. Phillips E. J. (2019). Antibiotic allergy. Lancet 393, 183–198. doi: 10.1016/S0140-6736(18)32218-9, PMID: 30558872 PMC6563335

[ref45] Brandariz-Nuñez D. Correas-Sanahuja M. Maya-Gallego S. Martín H. I. (2021). Neurotoxicity associated with acyclovir and valacyclovir: a systematic review of cases. J. Clin. Pharm. Ther. 46, 918–926. doi: 10.1111/jcpt.13464, PMID: 34146428

[ref46] Brandt R. Trushina N. I. Bakota L. (2020). Much more than a cytoskeletal protein: physiological and pathological functions of the non-microtubule binding region of tau. Front. Neurol. 11:590059. doi: 10.3389/fneur.2020.590059, PMID: 33193056 PMC7604284

[ref47] Bredesen D. E. (2014). Reversal of cognitive decline: a novel therapeutic program. Aging 6, 707–717. doi: 10.18632/aging.100690, PMID: 25324467 PMC4221920

[ref48] Bredesen D. E. Amos E. C. Canick J. Ackerley M. Raji C. Fiala M.. (2016). Reversal of cognitive decline in Alzheimer’s disease. Aging 8, 1250–1258. doi: 10.18632/aging.100981, PMID: 27294343 PMC4931830

[ref49] Brewer G. J. (2014). Alzheimer’s disease causation by copper toxicity and treatment with zinc. Front. Aging Neurosci. 6:92. doi: 10.3389/fnagi.2014.00092 24860501 PMC4030141

[ref50] Brown G. C. Heneka M. T. (2024). The endotoxin hypothesis of Alzheimer’s disease. Mol. Neurodegener. 19:30. doi: 10.1186/s13024-024-00722-y 38561809 PMC10983749

[ref51] Bubak A. N. Merle L. Niemeyer C. S. Baxter B. D. Gentile Polese A. Ramakrishnan V.. (2023). Signatures for viral infection and inflammation in the proximal olfactory system in familial Alzheimer’s disease. Neurobiol. Aging 123, 75–82. doi: 10.1016/j.neurobiolaging.2022.12.004, PMID: 36638683 PMC9889108

[ref52] Bulgart H. R. Neczypor E. W. Wold L. E. Mackos A. R. (2020). Microbial involvement in Alzheimer disease development and progression. Mol. Neurodegener. 15:42. doi: 10.1186/s13024-020-00378-4, PMID: 32709243 PMC7382139

[ref53] Burgos J. S. Ramirez C. Sastre I. Bullido M. J. Valdivieso F. (2003). ApoE4 is more efficient than E3 in brain access by herpes simplex virus type 1. Neuroreport 14, 1825–1827. doi: 10.1097/00001756-200310060-00013 14534428

[ref54] Burgos J. S. Ramirez C. Sastre I. Valdivieso F. (2006). Effect of apolipoprotein E on the cerebral load of latent herpes simplex virus type 1 DNA. J. Virol. 80, 5383–5387. doi: 10.1128/JVI.00006-06, PMID: 16699018 PMC1472141

[ref55] Caballero B. Bourdenx M. Luengo E. Diaz A. Sohn P. D. Chen X.. (2021). Acetylated tau inhibits chaperone-mediated autophagy and promotes tau pathology propagation in mice. Nat. Commun. 12, 1–18. doi: 10.1038/s41467-021-22501-9 33854069 PMC8047017

[ref56] Cai W. Li L. Sang S. Pan X. Zhong C. (2023). Physiological roles of β-amyloid in regulating synaptic function: implications for AD pathophysiology. Neurosci. Bull. 39, 1289–1308. doi: 10.1007/s12264-022-00985-9, PMID: 36443453 PMC10387033

[ref57] Canudas A. M. Gutierrez-Cuesta J. Rodríguez M. I. Acuña-Castroviejo D. Sureda F. X. Camins A.. (2005). Hyperphosphorylation of microtubule-associated protein tau in senescence-accelerated mouse (SAM). Mech. Ageing Dev. 126, 1300–1304. doi: 10.1016/j.mad.2005.07.008 16171847

[ref58] Cao X. Ren Y. Lu Q. Wang K. Wu Y. Wang Y.. (2023). Lactoferrin: A glycoprotein that plays an active role in human health. Front. Nutr. 9:1018336. doi: 10.3389/fnut.2022.1018336 36712548 PMC9875800

[ref59] Capetillo-Zarate E. Gracia L. Tampellini D. Gouras G. K. (2012). Intraneuronal Aβ accumulation, amyloid plaques, and synapse pathology in Alzheimer’s disease. Neurodegener. Dis. 10, 56–59. doi: 10.1159/000334762 22269167

[ref60] Capo C. R. Arciello M. Squitti R. Cassetta E. Rossini P. M. Calabrese L.. (2008). Features of ceruloplasmin in the cerebrospinal fluid of Alzheimer’s disease patients. Biometals 21, 367–372. doi: 10.1007/s10534-007-9125-4, PMID: 18060472

[ref61] Cardoso S. M. Proença M. T. Santos S. Santana I. Oliveira C. R. (2004). Cytochrome c oxidase is decreased in Alzheimer’s disease platelets. Neurobiol. Aging 25, 105–110. doi: 10.1016/S0197-4580(03)00033-2, PMID: 14675736

[ref62] Carmen R. Chong C.-Y. Method for inactivating viruses in blood using chlorine dioxide. US Patent. 5240829. (1993). Available at: https://patents.google.com/patent/US5240829A/en

[ref63] Carro E. Bartolomé F. Bermejo-Pareja F. Villarejo-Galende A. Molina J. A. Ortiz P.. (2017). Early diagnosis of mild cognitive impairment and Alzheimer’s disease based on salivary lactoferrin. Alzheimers Dement. 8, 131–138. doi: 10.1016/j.dadm.2017.04.002, PMID: 28649597 PMC5470603

[ref64] Catumbela C. S. G. Giridharan V. V. Barichello T. Morales R. (2023). Clinical evidence of human pathogens implicated in Alzheimer’s disease pathology and the therapeutic efficacy of antimicrobials: an overview. Transl. Neurodegener. 12:37. doi: 10.1186/s40035-023-00369-7, PMID: 37496074 PMC10369764

[ref65] Cavalier-Smith T. (2006). Origin of mitochondria by intracellular enslavement of a photosynthetic purple bacterium. Proc. Biol. Sci. 273, 1943–1952. doi: 10.1098/rspb.2006.3531 16822756 PMC1634775

[ref66] Chacko A. Delbaz A. Walkden H. Basu S. Armitage C. W. Eindorf T.. (2022). *Chlamydia pneumoniae* can infect the central nervous system via the olfactory and trigeminal nerves and contributes to Alzheimer’s disease risk. Sci. Rep. 12, 1–17. doi: 10.1038/s41598-022-06749-9 35177758 PMC8854390

[ref67] Chang Y.-J. Chen Y.-R. (2014). The coexistence of an equal amount of Alzheimer’s amyloid-β 40 and 42 forms structurally stable and toxic oligomers through a distinct pathway. FEBS J. 281, 2674–2687. doi: 10.1111/febs.12813 24720730

[ref68] Chávez-Reyes J. Escárcega-González C. E. Chavira-Suárez E. León-Buitimea A. Vázquez-León P. Morones-Ramírez J. R.. (2021). Susceptibility for some infectious diseases in patients with diabetes: the key role of glycemia. Front. Public Health 9:559595. doi: 10.3389/fpubh.2021.559595, PMID: 33665182 PMC7921169

[ref69] Chen L.-L. Fan Y.-G. Zhao L.-X. Zhang Q. Wang Z.-Y. (2023). The metal ion hypothesis of Alzheimer’s disease and the anti-neuroinflammatory effect of metal chelators. Bioorg. Chem. 131:106301. doi: 10.1016/j.bioorg.2022.106301, PMID: 36455485

[ref70] Chen J. Li T. Ye C. Zhong J. Huang J.-D. Ke Y.. (2023). The lung microbiome: a new frontier for lung and brain disease. Int. J. Mol. Sci. 24:2170. doi: 10.3390/ijms24032170, PMID: 36768494 PMC9916971

[ref71] Chen D. Liu X. Chen Y. Lin H. (2022). Amyloid peptides with antimicrobial and/or microbial agglutination activity. Appl. Microbiol. Biotechnol. 106, 7711–7720. doi: 10.1007/s00253-022-12246-w, PMID: 36322251 PMC9628408

[ref72] Chen S. G. Stribinskis V. Rane M. J. Demuth D. R. Gozal E. Roberts A. M.. (2016). Exposure to the functional bacterial amyloid protein curli enhances alpha-synuclein aggregation in aged Fischer 344 rats and *Caenorhabditis elegans*. Sci. Rep. 6:34477. doi: 10.1038/srep34477 27708338 PMC5052651

[ref73] Chen Y. Strickland M. R. Soranno A. Holtzman D. M. (2021). Apolipoprotein E: structural insights and links to Alzheimer’s disease pathogenesis. Neuron 109, 205–221. doi: 10.1016/j.neuron.2020.10.008, PMID: 33176118 PMC7931158

[ref74] Chen G.-F. Xu T.-H. Yan Y. Zhou Y.-R. Jiang Y. Melcher K.. (2017). Amyloid beta: structure, biology and structure-based therapeutic development. Acta Pharmacol. Sin. 38, 1205–1235. doi: 10.1038/aps.2017.28, PMID: 28713158 PMC5589967

[ref75] Cheng F. Peng G. Lu Y. Wang K. Ju Q. Ju Y.. (2022). Relationship between copper and immunity: the potential role of copper in tumor immunity. Front. Oncol. 12:1019153. doi: 10.3389/fonc.2022.1019153 36419894 PMC9676660

[ref76] Chida J. Hara H. Yano M. Uchiyama K. Das N. R. Takahashi E.. (2018). Prion protein protects mice from lethal infection with influenza A viruses. PLoS Pathog. 14:e1007049. doi: 10.1371/journal.ppat.1007049, PMID: 29723291 PMC5953499

[ref77] Ciudad S. Puig E. Botzanowski T. Meigooni M. Arango A. S. Do J.. (2020). Aβ(1-42) tetramer and octamer structures reveal edge conductivity pores as a mechanism for membrane damage. Nat. Commun. 11:3014. doi: 10.1038/s41467-020-16566-1, PMID: 32541820 PMC7296003

[ref78] Clement C. Hill J. M. Dua P. Culicchia F. Lukiw W. J. (2016). Analysis of RNA from Alzheimer’s disease post-mortem brain tissues. Mol. Neurobiol. 53, 1322–1328. doi: 10.1007/s12035-015-9105-6, PMID: 25631714 PMC5450164

[ref79] Congdon E. E. Ji C. Tetlow A. M. Jiang Y. Sigurdsson E. M. (2023). Tau-targeting therapies for Alzheimer disease: current status and future directions. Nat. Rev. Neurol. 19, 715–736. doi: 10.1038/s41582-023-00883-2, PMID: 37875627 PMC10965012

[ref80] Contaldi F. Capuano F. Fulgione A. Aiese Cigliano R. Sanseverino W. Iannelli D.. (2017). The hypothesis that *Helicobacter pylori* predisposes to Alzheimer’s disease is biologically plausible. Sci. Rep. 7, 1–14. doi: 10.1038/s41598-017-07532-x 28798312 PMC5552707

[ref81] Conti Filho C. E. Loss L. B. Marcolongo-Pereira C. Rossoni Junior J. V. Barcelos R. M. Chiarelli-Neto O.. (2023). Advances in Alzheimer’s disease’s pharmacological treatment. Front. Pharmacol. 14:1101452. doi: 10.3389/fphar.2023.1101452 36817126 PMC9933512

[ref82] Cooper J. M. Lathuiliere A. Migliorini M. Arai A. L. Wani M. M. Dujardin S.. (2021). Regulation of tau internalization, degradation, and seeding by LRP1 reveals multiple pathways for tau catabolism. J. Biol. Chem. 296:100715. doi: 10.1016/j.jbc.2021.100715, PMID: 33930462 PMC8164048

[ref83] Costa M. J. F. de Araújo I. D. T. da Rocha A. L. da Silva R. L. dos Santos C. P. Borges B. C. D.. (2021). Relationship of Porphyromonas gingivalis and Alzheimer’s disease: a systematic review of pre-clinical studies. Clin. Oral Investig. 25, 797–806. doi: 10.1007/s00784-020-03764-w, PMID: 33469718

[ref84] Cribbs D. H. Azizeh B. Y. Cotman C. W. LaFerla F. M. (2000). Fibril formation and neurotoxicity by a herpes simplex virus glycoprotein B fragment with homology to the Alzheimer’s Aβ peptide. Biochemistry 39, 5988–5994. doi: 10.1021/bi000029f, PMID: 10821670

[ref85] Cross A. J. Crow T. J. Perry E. K. Perry R. H. Blessed G. Tomlinson B. E. (1981). Reduced dopamine-beta-hydroxylase activity in Alzheimer’s disease. BMJ 282, 93–94. doi: 10.1136/bmj.282.6258.93, PMID: 6779929 PMC1503892

[ref86] D’Ambrosi N. Rossi L. (2015). Copper at synapse: release, binding and modulation of neurotransmission. Neurochem. Int. 90, 36–45. doi: 10.1016/j.neuint.2015.07.006, PMID: 26187063

[ref87] Dashper S. G. Pan Y. Veith P. D. Chen Y.-Y. Toh E. C. Y. Liu S. W.. (2012). Lactoferrin inhibits *Porphyromonas gingivalis* proteinases and has sustained biofilm inhibitory activity. Antimicrob. Agents Chemother. 56, 1548–1556. doi: 10.1128/AAC.05100-11, PMID: 22214780 PMC3294922

[ref88] de la Monte S. M. Wands J. R. (2008). Alzheimer’s disease is type 3 diabetes—evidence reviewed. J. Diabetes Sci. Technol. 2, 1101–1113. doi: 10.1177/193229680800200619, PMID: 19885299 PMC2769828

[ref89] De Lorenzi E. Chiari M. Colombo R. Cretich M. Sola L. Vanna R.. (2017). Evidence that the human innate immune peptide LL-37 may be a binding partner of amyloid-β and inhibitor of fibril assembly. J. Alzheimers Dis. 59, 1213–1226. doi: 10.3233/JAD-170223, PMID: 28731438 PMC5611894

[ref90] De Rodrigues P. S. Teixeira A. Kustner E. Medeiros R. (2015). Are herpes virus associated to aggressive periodontitis? A review of literature. J. Oral. Maxillofac Pathol. 19:348. doi: 10.4103/0973-029X.174621 26980964 PMC4774289

[ref91] Dekroon R. M. Armati P. J. (2001). Synthesis and processing of apolipoprotein E in human brain cultures. Glia 33, 298–305. doi: 10.1002/1098-1136(20010315)33:4<298::AID-GLIA1028>3.0.CO;2-N 11246228

[ref92] Derisbourg M. Leghay C. Chiappetta G. Fernandez-Gomez F.-J. Laurent C. Demeyer D.. (2015). Role of the tau N-terminal region in microtubule stabilization revealed by newendogenous truncated forms. Sci. Rep. 5:9659. doi: 10.1038/srep09659, PMID: 25974414 PMC4431475

[ref93] Dhillon S. (2021). Aducanumab: first approval. Drugs 81, 1437–1443. doi: 10.1007/s40265-021-01569-z 34324167

[ref94] Di Natale G. Bellia F. Sciacca M. F. M. Campagna T. Pappalardo G. (2018). Tau-peptide fragments and their copper(II) complexes: effects on amyloid-β aggregation. Inorganica Chim. Acta 472, 82–92. doi: 10.1016/j.ica.2017.09.061

[ref95] Ding Y. Ren J. Yu H. Yu W. Zhou Y. (2018). *Porphyromonas gingivalis*, a periodontitis causing bacterium, induces memory impairment and age-dependent neuroinflammation in mice. Immun. Ageing 15:6. doi: 10.1186/s12979-017-0110-7, PMID: 29422938 PMC5791180

[ref96] Dittbrenner H. (1994). Alzheimer's disease--the long goodbye. Caring 13:14–6, 18–20, 22–3, PMID: 10171925

[ref97] Dobson C. B. Sales S. D. Hoggard P. Wozniak M. A. Crutcher K. A. (2006). The receptor-binding region of human apolipoprotein E has direct anti-infective activity. J. Infect. Dis. 193, 442–450. doi: 10.1086/499280, PMID: 16388493

[ref98] Doig A. J. (2018). Positive feedback loops in Alzheimer’s disease: the Alzheimer’s feedback hypothesis. J. Alzheimers Dis. 66, 25–36. doi: 10.3233/JAD-180583, PMID: 30282364 PMC6484277

[ref99] Dominy S. S. Lynch C. Ermini F. Benedyk M. Marczyk A. Konradi A.. (2019). *Porphyromonas gingivalis* in Alzheimer’s disease brains: evidence for disease causation and treatment with small-molecule inhibitors. Sci. Adv. 5:eaau3333. doi: 10.1126/sciadv.aau3333, PMID: 30746447 PMC6357742

[ref100] Douros A. Ante Z. Fallone C. A. Azoulay L. Renoux C. Suissa S.. (2024). Clinically apparent *Helicobacter pylori* infection and the risk of incident Alzheimer’s disease: a population-based nested case-control study. Alzheimers Dement. 20, 1716–1724. doi: 10.1002/alz.13561, PMID: 38088512 PMC10984501

[ref101] Dreses-Werringloer U. Bhuiyan M. Zhao Y. Gérard H. C. Whittum-Hudson J. A. Hudson A. P. (2009). Initial characterization of Chlamydophila (Chlamydia) pneumoniae cultured from the late-onset Alzheimer brain. Int. J. Med. Microbiol. 299, 187–201. doi: 10.1016/j.ijmm.2008.07.002 18829386 PMC2730674

[ref102] Drew S. C. (2017). The case for abandoning therapeutic chelation of copper ions in Alzheimer’s disease. Front. Neurosci. 11:503. doi: 10.3389/fnins.2017.00317, PMID: 28626387 PMC5455140

[ref103] Duarte L. F. Farías M. A. Álvarez D. M. Bueno S. M. Riedel C. A. González P. A. (2019). Herpes simplex virus type 1 infection of the central nervous system: insights into proposed interrelationships with neurodegenerative disorders. Front. Cell. Neurosci. 13:46. doi: 10.3389/fncel.2019.00046 30863282 PMC6399123

[ref104] Eimer W. A. Vijaya Kumar D. K. Navalpur Shanmugam N. K. Rodriguez A. S. Mitchell T. Washicosky K. J.. (2018). Alzheimer’s disease-associated β-amyloid is rapidly seeded by herpesviridae to protect against brain infection. Neuron 99, 56–63.e3. doi: 10.1016/j.neuron.2018.06.030, PMID: 30001512 PMC6075814

[ref105] El Karim I. A. Linden G. J. Orr D. F. Lundy F. T. (2008). Antimicrobial activity of neuropeptides against a range of micro-organisms from skin, oral, respiratory and gastrointestinal tract sites. J. Neuroimmunol. 200, 11–16. doi: 10.1016/j.jneuroim.2008.05.014, PMID: 18603306

[ref106] Elie A. Prezel E. Guérin C. Denarier E. Ramirez-Rios S. Serre L.. (2015). Tau co-organizes dynamic microtubule and actin networks. Sci. Rep. 5:9964. doi: 10.1038/srep09964 25944224 PMC4421749

[ref107] Eljaaly K. Alshehri S. Bhattacharjee S. Al-Tawfiq J. A. Patanwala A. E. (2019). Contraindicated drug–drug interactions associated with oral antimicrobial agents prescribed in the ambulatory care setting in the United States. Clin. Microbiol. Infect. 25, 620–622. doi: 10.1016/j.cmi.2018.08.002, PMID: 30107284

[ref108] Elkins M. Jain N. Tükel Ç. (2024). The menace within: bacterial amyloids as a trigger for autoimmune and neurodegenerative diseases. Curr. Opin. Microbiol. 79:102473. doi: 10.1016/j.mib.2024.102473, PMID: 38608623 PMC11162901

[ref109] Elonheimo H. M. Andersen H. R. Katsonouri A. Tolonen H. (2021). Environmental substances associated with Alzheimer’s disease—a scoping review. Int. J. Environ. Res. Public Health 18:11839. doi: 10.3390/ijerph182211839, PMID: 34831595 PMC8622417

[ref110] Engelberg Y. Landau M. (2020). The human LL-37(17-29) antimicrobial peptide reveals a functional supramolecular structure. Nat. Commun. 11:3894. doi: 10.1038/s41467-020-17736-x, PMID: 32753597 PMC7403366

[ref111] Espay A. J. Herrup K. Daly T. (2023). “Finding the falsification threshold of the toxic proteinopathy hypothesis in neurodegeneration” in Precision medicine in neurodegenerative disorders, part I. Ed. A. J. Espay (Oxford, UK: Elsevier), 143–154. 10.1016/B978-0-323-85538-9.00008-0 36796939

[ref112] Espinosa-Cantú A. Cruz-Bonilla E. Noda-Garcia L. DeLuna A. (2020). Multiple forms of multifunctional proteins in health and disease. Front. Cell Dev. Biol. 8:451. doi: 10.3389/fcell.2020.00451, PMID: 32587857 PMC7297953

[ref113] EU/3/13/1139. Orphan designation for treatment of amyotrophic lateral sclerosis. (2024) Available at: https://www.ema.europa.eu/en/medicines/human/orphan-designations/eu-3-13-1139

[ref114] Fan Y.-G. Guo C. Zhao L.-X. Ge R.-L. Pang Z.-Q. He D.-L.. (2024). Astrocyte-derived lactoferrin reduces β-amyloid burden by promoting the interaction between p38 kinase and PP2A phosphatase in male APP/PS1 transgenic mice. Br. J. Pharmacol. 181, 896–913. doi: 10.1111/bph.16161, PMID: 37309219

[ref115] Feng S. Liu Y. Zhou Y. Shu Z. Cheng Z. Brenner C.. (2023). Mechanistic insights into the role of herpes simplex virus 1 in Alzheimer’s disease. Front. Aging Neurosci. 15:1245904. doi: 10.3389/fnagi.2023.1245904, PMID: 37744399 PMC10512732

[ref116] Feng J. She Y. Li C. Shen L. (2023). Metal ion mediated aggregation of Alzheimer’s disease peptides and proteins in solutions and at surfaces. Adv. Colloid Interf. Sci. 320:103009. doi: 10.1016/j.cis.2023.103009, PMID: 37776735

[ref117] Feng W. Ye F. Xue W. Zhou Z. Kang Y. J. (2009). Copper regulation of hypoxia-inducible factor-1 activity. Mol. Pharmacol. 75, 174–182. doi: 10.1124/mol.108.051516, PMID: 18842833 PMC2685058

[ref118] Fernández-Calle R. Konings S. C. Frontiñán-Rubio J. García-Revilla J. Camprubí-Ferrer L. Svensson M.. (2022). APOE in the Bullseye of neurodegenerative diseases: impact of the APOE genotype in Alzheimer’s disease pathology and brain diseases. Mol. Neurodegener. 17:62. doi: 10.1186/s13024-022-00566-4, PMID: 36153580 PMC9509584

[ref119] Fernández-Calvet A. Matilla-Cuenca L. Izco M. Navarro S. Serrano M. Ventura S.. (2024). Gut microbiota produces biofilm-associated amyloids with potential for neurodegeneration. Nat. Commun. 15, 1–19. doi: 10.1038/s41467-024-48309-x 38755164 PMC11099085

[ref120] Ferreira D. Perestelo-Pérez L. Westman E. Wahlund L.-O. Sarría A. Serrano-Aguilar P. (2014). Meta-review of CSF core biomarkers in Alzheimer’s disease: the state-of-the-art after the new revised diagnostic criteria. Front. Aging Neurosci. 6:47. doi: 10.3389/fnagi.2014.00047 24715863 PMC3970033

[ref121] Ferrer-Luque C. M. Solana C. Aguado B. Baca P. Arias-Moliz M. T. Ruiz-Linares M. (2023). Efficacy of mixed diclofenac solutions against root canal biofilms. Aust. Endod. J. 49, 530–536. doi: 10.1111/aej.12776, PMID: 37464569

[ref122] Fillebeen C. Ruchoux M.-M. Mitchell V. Vincent S. Benaıssa M. Pierce A. (2001). Lactoferrin is synthesized by activated microglia in the human substantia nigra and its synthesis by the human microglial CHME cell line is upregulated by tumor necrosis factor α or 1-methyl-4-phenylpyridinium treatment. Brain Res. Mol. Brain Res. 96, 103–113. doi: 10.1016/S0169-328X(01)00216-9 11731015

[ref123] Flowers S. A. Rebeck G. W. (2020). APOE in the normal brain. Neurobiol. Dis. 136:104724. doi: 10.1016/j.nbd.2019.104724, PMID: 31911114 PMC7002287

[ref124] Focarelli F. Giachino A. Waldron K. J. (2022). Copper microenvironments in the human body define patterns of copper adaptation in pathogenic bacteria. PLoS Pathog. 18:e1010617. doi: 10.1371/journal.ppat.1010617, PMID: 35862345 PMC9302775

[ref125] Fortea J. Pegueroles J. Alcolea D. Belbin O. Dols-Icardo O. Vaqué-Alcázar L.. (2024). APOE4 homozygosity represents a distinct genetic form of Alzheimer’s disease. Nat. Med. 30, 1284–1291. doi: 10.1038/s41591-024-02931-w, PMID: 38710950 PMC13310155

[ref126] Franceschi F. Ojetti V. Candelli M. Covino M. Cardone S. Potenza A.. (2019). Microbes and Alzheimer’ disease: lessons from H. Pylori and GUT microbiota. Eur. Rev. Med. Pharmacol. Sci. 23, 426–430. doi: 10.26355/eurrev_201901_16791, PMID: 30657587

[ref127] Frieden C. Wang H. Ho C. M. W. (2017). A mechanism for lipid binding to apoE and the role of intrinsically disordered regions coupled to domain–domain interactions. Proc. Natl. Acad. Sci. USA 114, 6292–6297. doi: 10.1073/pnas.1705080114, PMID: 28559318 PMC5474821

[ref128] Friedland R. P. Chapman M. R. (2017). The role of microbial amyloid in neurodegeneration. PLoS Pathog. 13:e1006654. doi: 10.1371/journal.ppat.1006654, PMID: 29267402 PMC5739464

[ref129] Frisoni G. B. Altomare D. Thal D. R. Ribaldi F. van der Kant R. Ossenkoppele R.. (2022). The probabilistic model of Alzheimer disease: the amyloid hypothesis revised. Nat. Rev. Neurosci. 23, 53–66. doi: 10.1038/s41583-021-00533-w, PMID: 34815562 PMC8840505

[ref130] Fruhwürth S. Reinert L. S. Öberg C. Sakr M. Henricsson M. Zetterberg H.. (2023). The microglia receptor protein TREM2 is essential for protective innate immune responses against herpesvirus infection in the brain. bioRxiv. Available at: http://biorxiv.org/content/early/2023/05/23/2023.03.16.532882.abstract

[ref131] Fullerton S. M. Clark A. G. Weiss K. M. Nickerson D. A. Taylor S. L. Stengård J. H.. (2000). Apolipoprotein E variation at the sequence haplotype level: implications for the origin and maintenance of a major human polymorphism. Am. J. Hum. Genet. 67, 881–900. doi: 10.1086/303070, PMID: 10986041 PMC1287893

[ref132] Fülöp T. Itzhaki R. F. Balin B. J. Miklossy J. Barron A. E. (2018). Role of microbes in the development of Alzheimer’s disease: state of the art – an international symposium presented at the 2017 IAGG congress in San Francisco. Front. Genet. 9:362. doi: 10.3389/fgene.2018.00362 30250480 PMC6139345

[ref133] Gao S. S. Chu C. H. Young F. Y. F. (2020). Oral health and care for elderly people with Alzheimer’s disease. Int. J. Environ. Res. Public Health 17:5713. doi: 10.3390/ijerph17165713, PMID: 32784777 PMC7460333

[ref134] Garai K. Verghese P. B. Baban B. Holtzman D. M. Frieden C. (2014). The binding of apolipoprotein E to oligomers and fibrils of amyloid-β alters the kinetics of amyloid aggregation. Biochemistry 53, 6323–6331. doi: 10.1021/bi5008172, PMID: 25207746 PMC4196732

[ref135] Gérard H. C. Fomicheva E. Whittum-Hudson J. A. Hudson A. P. (2008). Apolipoprotein E4 enhances attachment of Chlamydophila (Chlamydia) pneumoniae elementary bodies to host cells. Microb. Pathog. 44, 279–285. doi: 10.1016/j.micpath.2007.10.002, PMID: 17997273

[ref136] Gérard H. C. Wildt K. L. Whittum-Hudson J. A. Lai Z. Ager J. Hudson A. P. (2005). The load of *Chlamydia pneumoniae* in the Alzheimer’s brain varies with APOE genotype. Microb. Pathog. 39, 19–26. doi: 10.1016/j.micpath.2005.05.002 15998578

[ref137] Giuffrida M. L. Caraci F. Pignataro B. Cataldo S. De Bona P. Bruno V.. (2009). Β-amyloid monomers are neuroprotective. J. Neurosci. 29, 10582–10587. doi: 10.1523/JNEUROSCI.1736-09.2009, PMID: 19710311 PMC6665714

[ref138] Goedert M. Spillantini M. G. Jakes R. Rutherford D. Crowther R. A. (1989). Multiple isoforms of human microtubule-associated protein tau: sequences and localization in neurofibrillary tangles of Alzheimer’s disease. Neuron 3, 519–526. doi: 10.1016/0896-6273(89)90210-9 2484340

[ref139] Goel P. Chakrabarti S. Goel K. Bhutani K. Chopra T. Bali S. (2022). Neuronal cell death mechanisms in Alzheimer’s disease: an insight. Front. Mol. Neurosci. 15:937133. doi: 10.3389/fnmol.2022.937133, PMID: 36090249 PMC9454331

[ref140] Goldman S. M. Quinlan P. J. Ross G. W. Marras C. Meng C. Bhudhikanok G. S.. (2012). Solvent exposures and parkinson disease risk in twins. Ann. Neurol. 71, 776–784. doi: 10.1002/ana.22629, PMID: 22083847 PMC3366287

[ref141] Gomez W. Morales R. Maracaja-Coutinho V. Parra V. Nassif M. (2020). Down syndrome and Alzheimer’s disease: common molecular traits beyond the amyloid precursor protein. Aging 12, 1011–1033. doi: 10.18632/aging.102677, PMID: 31918411 PMC6977673

[ref142] Gomez-Nicola D. Boche D. (2015). Post-mortem analysis of neuroinflammatory changes in human Alzheimer’s disease. Alzheimers Res. Ther. 7:42. doi: 10.1186/s13195-015-0126-1, PMID: 25904988 PMC4405851

[ref143] Gong C.-X. Iqbal K. (2008). Hyperphosphorylation of microtubule-associated protein tau: a promising therapeutic target for Alzheimer disease. Curr. Med. Chem. 15, 2321–2328. doi: 10.2174/092986708785909111, PMID: 18855662 PMC2656563

[ref144] Gong C.-X. Liu F. Grundke-Iqbal I. Iqbal K. (2005). Post-translational modifications of tau protein in Alzheimer’s disease. J. Neural Transm. 112, 813–838. doi: 10.1007/s00702-004-0221-0 15517432

[ref145] González-Sánchez M. Bartolome F. Antequera D. Puertas-Martín V. González P. Gómez-Grande A.. (2020). Decreased salivary lactoferrin levels are specific to Alzheimer’s disease. EBioMedicine 57:102834. doi: 10.1016/j.ebiom.2020.102834, PMID: 32586758 PMC7378957

[ref146] Gouras G. K. Willén K. Faideau M. (2014). The inside-out amyloid hypothesis and synapse pathology in Alzheimer’s disease. Neurodegener Dis 13, 142–146. doi: 10.1159/000354776, PMID: 24080821

[ref147] Griciuc A. Tanzi R. E. (2021). The role of innate immune genes in Alzheimer’s disease. Curr. Opin. Neurol. 34, 228–236. doi: 10.1097/WCO.0000000000000911, PMID: 33560670 PMC7954128

[ref148] Grubman A. Chew G. Ouyang J. F. Sun G. Choo X. Y. McLean C.. (2019). A single-cell atlas of entorhinal cortex from individuals with Alzheimer’s disease reveals cell-type-specific gene expression regulation. Nat. Neurosci. 22, 2087–2097. doi: 10.1038/s41593-019-0539-4, PMID: 31768052

[ref149] Gruden Š. Poklar U. N. (2021). Diverse mechanisms of antimicrobial activities of lactoferrins, lactoferricins, and other lactoferrin-derived peptides. Int. J. Mol. Sci. 22:11264. doi: 10.3390/ijms222011264, PMID: 34681923 PMC8541349

[ref150] Guerreiro R. Wojtas A. Bras J. Carrasquillo M. Rogaeva E. Majounie E.. (2013). *TREM2* variants in Alzheimer’s disease. N. Engl. J. Med. 368, 117–127. doi: 10.1056/NEJMoa1211851, PMID: 23150934 PMC3631573

[ref151] Guo T. Noble W. Hanger D. P. (2017). Roles of tau protein in health and disease. Acta Neuropathol. 133, 665–704. doi: 10.1007/s00401-017-1707-9, PMID: 28386764 PMC5390006

[ref152] Gutacker M. Valsangiacomo C. Balmelli T. Bernasconi M. V. Bouras C. Piffaretti J.-C. (1998). Arguments against the involvement of *Borrelia burgdorferi* sensu lato in Alzheimer’s disease. Res. Microbiol. 149, 31–37. doi: 10.1016/S0923-2508(97)83621-2, PMID: 9766207

[ref153] Haditsch U. Roth T. Rodriguez L. Hancock S. Cecere T. Nguyen M.. (2020). Alzheimer’s disease-like neurodegeneration in *Porphyromonas gingivalis* infected neurons with persistent expression of active gingipains. J. Alzheimers Dis. 75, 1361–1376. doi: 10.3233/JAD-200393, PMID: 32390638 PMC7369049

[ref154] Hajishengallis G. Darveau R. P. Curtis M. A. (2012). The keystone-pathogen hypothesis. Nat. Rev. Microbiol. 10, 717–725. doi: 10.1038/nrmicro2873, PMID: 22941505 PMC3498498

[ref155] Hampel H. Hardy J. Blennow K. Chen C. Perry G. Kim S. H.. (2021). The amyloid-β pathway in Alzheimer’s disease. Mol. Psychiatry 26, 5481–5503. doi: 10.1038/s41380-021-01249-0, PMID: 34456336 PMC8758495

[ref156] Haney M. S. Pálovics R. Munson C. N. Long C. Johansson P. K. Yip O.. (2024). APOE4/4 is linked to damaging lipid droplets in Alzheimer’s disease microglia. Nature 628, 154–161. doi: 10.1038/s41586-024-07185-7, PMID: 38480892 PMC10990924

[ref157] Harrington J. P. Stuart J. Jones A. (1987). Unfolding of iron and copper complexes of human lactoferrin and transferrin. Int. J. Biochem. 19, 1001–1008. doi: 10.1016/0020-711X(87)90184-4, PMID: 3666279

[ref158] Harrison M. A. A. Morris S. L. Rudman G. A. Rittenhouse D. J. Monk C. H. Sakamuri S. S. V. P.. (2024). Intermittent cytomegalovirus infection alters neurobiological metabolism and induces cognitive deficits in mice. Brain Behav. Immun. 117, 36–50. doi: 10.1016/j.bbi.2023.12.033, PMID: 38182037 PMC11914963

[ref159] Hathroubi S. Servetas S. L. Windham I. Merrell D. S. Ottemann K. M. (2018). *Helicobacter pylori* biofilm formation and its potential role in pathogenesis. Microb. Mol. Biol. Rev. 82:e00001-18. doi: 10.1128/MMBR.00001-18 PMC5968456 29743338

[ref160] Hernández-Ortega K. Garcia-Esparcia P. Gil L. Lucas J. J. Ferrer I. (2016). Altered machinery of protein synthesis in Alzheimer’s: from the nucleolus to the ribosome. Brain Pathol. 26, 593–605. doi: 10.1111/bpa.12335, PMID: 26512942 PMC8029302

[ref161] Herz J. (2009). Apolipoprotein E receptors in the nervous system. Curr. Opin. Lipidol. 20, 190–196. doi: 10.1097/MOL.0b013e32832d3a10, PMID: 19433918 PMC2848396

[ref162] Hilton J. B. W. Kysenius K. Liddell J. R. Mercer S. W. Paul B. Beckman J. S.. (2024). Evidence for disrupted copper availability in human spinal cord supports CuII(atsm) as a treatment option for sporadic cases of ALS. Sci. Rep. 14, 1–9. doi: 10.1038/s41598-024-55832-w 38467696 PMC10928073

[ref163] Høiby N. Bjarnsholt T. Givskov M. Molin S. Ciofu O. (2010). Antibiotic resistance of bacterial biofilms. Int. J. Antimicrob. Agents 35, 322–332. doi: 10.1016/j.ijantimicag.2009.12.011, PMID: 20149602

[ref164] Høilund-Carlsen P. F. Alavi A. (2021). Aducanumab (marketed as Aduhelm) approval is likely based on misinterpretation of PET imaging data. J. Alzheimers Dis. 84, 1457–1460. doi: 10.3233/JAD-215275, PMID: 34657891

[ref165] Høilund-Carlsen P. F. Revheim M.-E. Alavi A. Barrio J. R. (2023). FDG PET (and MRI) for monitoring immunotherapy in Alzheimer disease. Clin. Nucl. Med. 48, 689–691. doi: 10.1097/RLU.0000000000004710 37314733 PMC10317300

[ref166] Hong J.-M. Munna A. N. Moon J.-H. Kim J.-H. Seol J.-W. Eo S.-K.. (2023). Antiviral activity of prion protein against Japanese encephalitis virus infection in vitro and in vivo. Virus Res. 338:199249. doi: 10.1016/j.virusres.2023.199249, PMID: 37858731 PMC10598702

[ref167] How K. Y. Song K. P. Chan K. G. (2016). *Porphyromonas gingivalis*: an overview of periodontopathic pathogen below the gum line. Front. Microbiol. 7:53. doi: 10.3389/fmicb.2016.00053 26903954 PMC4746253

[ref168] Hsu H.-W. Rodriguez-Ortiz C. J. Lim S. L. Zumkehr J. Kilian J. G. Vidal J.. (2019). Copper-induced upregulation of MicroRNAs directs the suppression of endothelial LRP1 in Alzheimer’s disease model. Toxicol. Sci. 170, 144–156. doi: 10.1093/toxsci/kfz084, PMID: 30923833 PMC6592190

[ref169] Hu K. Babapoor-Farrokhran S. Rodrigues M. Deshpande M. Puchner B. Kashiwabuchi F.. (2016). Hypoxia-inducible factor 1 upregulation of both VEGF and ANGPTL4 is required to promote the angiogenic phenotype in uveal melanoma. Oncotarget 7, 7816–7828. doi: 10.18632/oncotarget.6868, PMID: 26761211 PMC4884956

[ref170] Huang Y. Happonen K. E. Burrola P. G. O’Connor C. Hah N. Huang L.. (2021). Microglia use TAM receptors to detect and engulf amyloid β plaques. Nat. Immunol. 22, 586–594. doi: 10.1038/s41590-021-00913-5, PMID: 33859405 PMC8102389

[ref171] Huang Y. Mahley R. W. (2014). Apolipoprotein E: structure and function in lipid metabolism, neurobiology, and Alzheimer’s diseases. Neurobiol. Dis. 72, 3–12. doi: 10.1016/j.nbd.2014.08.025 25173806 PMC4253862

[ref172] Huang D. Y. Weisgraber K. H. Goedert M. Saunders A. M. Roses A. D. Strittmatter W. J. (1995). ApoE3 binding to tau tandem repeat I is abolished by tau serine262 phosphorylation. Neurosci. Lett. 192, 209–212. doi: 10.1016/0304-3940(95)11649-H 7566652

[ref173] Hunter L. Wood D. Dargan P. I. (2011). The patterns of toxicity and management of acute nonsteroidal anti-inflammatory drug (NSAID) overdose. Open Access Emerg. Med. 3:39. doi: 10.2147/OAEM.S22795 27147851 PMC4753966

[ref174] Ilievski V. Zuchowska P. K. Green S. J. Toth P. T. Ragozzino M. E. Le K.. (2018). Chronic oral application of a periodontal pathogen results in brain inflammation, neurodegeneration and amyloid beta production in wild type mice. PLoS One 13:e0204941. doi: 10.1371/journal.pone.0204941, PMID: 30281647 PMC6169940

[ref175] Itzhaki R. F. (2018). Corroboration of a major role for herpes simplex virus type 1 in Alzheimer’s disease. Front. Aging Neurosci. 10:379388. doi: 10.3389/fnagi.2018.00324 PMC6202583 30405395

[ref176] Itzhaki R. F. (2021). Overwhelming evidence for a major role for herpes simplex virus type 1 (HSV1) in Alzheimer’s disease (AD); underwhelming evidence against. Vaccines 9:679. doi: 10.3390/vaccines9060679, PMID: 34205498 PMC8234998

[ref177] Itzhaki R. F. (2022). Does antiherpetic antiviral therapy reduce the risk of dementia? Nat. Rev. Neurol. 18, 63–64. doi: 10.1038/s41582-021-00596-4, PMID: 34873309

[ref178] Itzhaki R. F. Lathe R. Balin B. J. Ball M. J. Bearer E. L. Braak H.. (2016). Microbes and Alzheimer’s disease. J. Alzheimers Dis. 51, 979–984. doi: 10.3233/JAD-160152, PMID: 26967229 PMC5457904

[ref179] Iwai Y. Shibuya K. Misawa S. Sekiguchi Y. Watanabe K. Amino H.. (2016). Axonal dysfunction precedes motor neuronal death in amyotrophic lateral sclerosis. PLoS One 11:e0158596. doi: 10.1371/journal.pone.0158596 27383069 PMC4934877

[ref180] Jia L. Jiang Y. Wu L. Fu J. Du J. Luo Z.. (2024). *Porphyromonas gingivalis* aggravates colitis via a gut microbiota-linoleic acid metabolism-Th17/Treg cell balance axis. Nat. Commun. 15, 1–18. doi: 10.1038/s41467-024-45473-y 38388542 PMC10883948

[ref181] Jiang S. Li X. Li Y. Chang Z. Yuan M. Zhang Y.. (2024). APOE from patient-derived astrocytic extracellular vesicles alleviates neuromyelitis optica spectrum disorder in a mouse model. Sci. Transl. Med. 16:eadg5116. doi: 10.1126/scitranslmed.adg5116, PMID: 38416841

[ref182] Jiang D. Zhang L. Grant G. P. G. Dudzik C. G. Chen S. Patel S.. (2013). The elevated copper binding strength of amyloid-β aggregates allows the sequestration of copper from albumin: a pathway to accumulation of copper in senile plaques. Biochemistry 52, 547–556. doi: 10.1021/bi301053h, PMID: 23237523 PMC3552001

[ref183] Jin L. Wu W.-H. Li Q.-Y. Zhao Y.-F. Li Y.-M. (2011). Copper inducing Aβ42 rather than Aβ40 nanoscale oligomer formation is the key process for Aβ neurotoxicity. Nanoscale 3, 4746–4751. doi: 10.1039/c1nr11029b, PMID: 21952557

[ref184] Johnson G. V. W. Stoothoff W. H. (2004). Tau phosphorylation in neuronal cell function and dysfunction. J. Cell Sci. 117, 5721–5729. doi: 10.1242/jcs.01558 15537830

[ref185] Kahlenberg J. M. Kaplan M. J. (2013). Little peptide, big effects: the role of LL-37 in inflammation and autoimmune disease. J. Immunol. 191, 4895–4901. doi: 10.4049/jimmunol.1302005, PMID: 24185823 PMC3836506

[ref186] Kalghatgi S. Spina C. S. Costello J. C. Liesa M. Morones-Ramirez J. R. Slomovic S.. (2013). Bactericidal antibiotics induce mitochondrial dysfunction and oxidative damage in mammalian cells. Sci. Transl. Med. 5:192ra85. doi: 10.1126/scitranslmed.3006055 PMC3760005 23825301

[ref187] Kametani F. Hasegawa M. (2018). Reconsideration of amyloid hypothesis and tau hypothesis in Alzheimer’s disease. Front. Neurosci. 12:25. doi: 10.3389/fnins.2018.00025, PMID: 29440986 PMC5797629

[ref188] Kanagasingam S. von Ruhland C. Welbury R. Singhrao S. K. (2022). Antimicrobial, polarizing light, and paired helical filament properties of fragmented tau peptides of selected putative gingipains. J. Alzheimers Dis. 89, 1279–1291. doi: 10.3233/JAD-220486, PMID: 36031895

[ref189] Karahan H. Dabin L. C. Tate M. D. Kim J. (2021). MicroRNAs on the move: microRNAs in astrocyte-derived ApoE particles regulate neuronal function. Neuron 109, 907–909. doi: 10.1016/j.neuron.2021.02.021, PMID: 33735610

[ref190] Karran E. Mercken M. Strooper B. D. (2011). The amyloid cascade hypothesis for Alzheimer’s disease: an appraisal for the development of therapeutics. Nat. Rev. Drug Discov. 10, 698–712. doi: 10.1038/nrd3505, PMID: 21852788

[ref191] Kavanagh T. Halder A. Drummond E. (2022). Tau interactome and RNA binding proteins in neurodegenerative diseases. Mol. Neurodegener. 17:66. doi: 10.1186/s13024-022-00572-6 36253823 PMC9575286

[ref192] Kawahara M. Kato-Negishi M. (2011). Link between aluminum and the pathogenesis of Alzheimer′s disease: the integration of the aluminum and amyloid cascade hypotheses. Int. J. Alzheimers Dis. 2011:276393. doi: 10.4061/2011/276393 21423554 PMC3056430

[ref193] Kettunen P. Koistinaho J. Rolova T. (2024). Contribution of CNS and extra-CNS infections to neurodegeneration: a narrative review. J. Neuroinflammation. 21:152. doi: 10.1186/s12974-024-03139-y, PMID: 38845026 PMC11157808

[ref194] Khurshid Z. Najeeb S. Mali M. Moin S. F. Raza S. Q. Zohaib S.. (2017). Histatin peptides: pharmacological functions and their applications in dentistry. Saudi Pharm J. 25, 25–31. doi: 10.1016/j.jsps.2016.04.027, PMID: 28223859 PMC5310145

[ref195] Kieburtz K. Dorsey E. R. (2021). Parkinson disease risks: correctly identifying environmental factors for a chronic disease. J. Clin. Invest. 131:e150252. doi: 10.1172/JCI150252 34060482 PMC8159678

[ref196] Kim J. Basak J. M. Holtzman D. M. (2009). The role of apolipoprotein E in Alzheimer’s disease. Neuron 63, 287–303. doi: 10.1016/j.neuron.2009.06.026, PMID: 19679070 PMC3044446

[ref197] Kim M. Park S. J. Choi S. Chang J. Kim S. M. Jeong S.. (2022). Association between antibiotics and dementia risk: a retrospective cohort study. Front. Pharmacol. 13:888333. doi: 10.3389/fphar.2022.888333, PMID: 36225572 PMC9548656

[ref198] Kivimäki M. Walker K. A. (2024). Severe infections as a gateway to dementia. Nat. Aging. 4, 752–754. doi: 10.1038/s43587-024-00643-x, PMID: 38783151

[ref199] Kobayashi N. Masuda J. Kudoh J. BindingSites on tau proteins as components for antimicrobial peptides. (2008). Available at: https://www.jstage.jst.go.jp/article/bio1996/13/2/13_2_49/_pdf 10.4265/bio.13.49 18661680

[ref200] Kogon A. Kronmal R. Peterson D. R. (1968). The relationship between infectious hepatitis and Down’s syndrome. Am. J. Public Health Nations Health 58, 305–311. doi: 10.2105/AJPH.58.2.305, PMID: 4229823 PMC1228157

[ref201] Köhler C. Maes M. Slyepchenko A. Berk M. Solmi M. Lanctôt K.. (2016). The gut-brain axis, including the microbiome, leaky gut and bacterial translocation: mechanisms and pathophysiological role in Alzheimer’s disease. Curr. Pharm. Des. 22, 6152–6166. doi: 10.2174/1381612822666160907093807, PMID: 27604604

[ref202] Kong G. K.-W. Miles L. A. Crespi G. A. N. Morton C. J. Ng H. L. Barnham K. J.. (2008). Copper binding to the Alzheimer’s disease amyloid precursor protein. Eur. Biophys. J. 37, 269–279. doi: 10.1007/s00249-007-0234-3, PMID: 18030462 PMC2921068

[ref203] Konings S. C. Torres-Garcia L. Martinsson I. Gouras G. K. (2021). Astrocytic and neuronal apolipoprotein E isoforms differentially affect neuronal excitability. Front. Neurosci. 15:734001. doi: 10.3389/fnins.2021.734001, PMID: 34621153 PMC8490647

[ref204] Korvatska O. Kiianitsa K. Ratushny A. Matsushita M. Beeman N. Chien W.-M.. (2020). Triggering receptor expressed on myeloid cell 2 R47H exacerbates immune response in Alzheimer’s disease brain. Front. Immunol. 11:559342. doi: 10.3389/fimmu.2020.559342 33101276 PMC7546799

[ref205] Kountouras J. Boziki M. Gavalas E. Zavos C. Deretzi G. Grigoriadis N.. (2009). Increased cerebrospinal fluid *Helicobacter pylori* antibody in Alzheimer’s disease. Int. J. Neurosci. 119, 765–777. doi: 10.1080/00207450902782083, PMID: 19326283

[ref206] Kowalczyk P. Kaczyńska K. Kleczkowska P. Bukowska-Ośko I. Kramkowski K. Sulejczak D. (2022). The lactoferrin phenomenon—a miracle molecule. Molecules 27:2941. doi: 10.3390/molecules27092941, PMID: 35566292 PMC9104648

[ref207] Kross R. D. Scheer D. I. Composition and procedure for disinfecting blood and blood components. US Patent. 5019402 (1991). Available at: https://patents.google.com/patent/US5019402A/en

[ref208] Kumar D. K. V. Choi S. H. Washicosky K. J. Eimer W. A. Tucker S. Ghofrani J.. (2016). Amyloid-β peptide protects against microbial infection in mouse and worm models of Alzheimer’s disease. Sci. Transl. Med. 8:340ra72. doi: 10.1126/scitranslmed.aaf1059 PMC5505565 27225182

[ref209] Kumar A. Sidhu J. Goyal A. Tsao J. W. (2022). Alzheimer disease. Treasure Island, (FL): StatPearls Publishing.

[ref210] Kurokawa C. Lynn G. E. Pedra J. H. F. Pal U. Narasimhan S. Fikrig E. (2020). Interactions between Borrelia burgdorferi and ticks. Nat. Rev. Microbiol. 18, 587–600. doi: 10.1038/s41579-020-0400-5, PMID: 32651470 PMC7351536

[ref211] Lanfranco M. F. Sepulveda J. Kopetsky G. Rebeck G. W. (2021). Expression and secretion of apoE isoforms in astrocytes and microglia during inflammation. Glia 69, 1478–1493. doi: 10.1002/glia.23974, PMID: 33556209 PMC8717762

[ref212] Lee W. J. Brown J. A. Kim H. R. La Joie R. Cho H. Lyoo C. H.. (2022). Regional Aβ-tau interactions promote onset and acceleration of Alzheimer’s disease tau spreading. Neuron 110, 1932–1943.e5. doi: 10.1016/j.neuron.2022.03.034, PMID: 35443153 PMC9233123

[ref213] Lee M. Shi X. Barron A. E. McGeer E. McGeer P. L. (2015). Human antimicrobial peptide LL-37 induces glial-mediated neuroinflammation. Biochem. Pharmacol. 94, 130–141. doi: 10.1016/j.bcp.2015.02.003, PMID: 25686659

[ref214] Lee E. Y. Srinivasan Y. de Anda J. Nicastro L. K. Tükel Ç. Wong G. C. L. (2020). Functional reciprocity of amyloids and antimicrobial peptides: rethinking the role of supramolecular assembly in host defense, immune activation, and inflammation. Front. Immunol. 11:1629. doi: 10.3389/fimmu.2020.01629 32849553 PMC7412598

[ref215] Lei S. Li J. Yu J. Li F. Pan Y. Chen X.. (2023). *Porphyromonas gingivalis* bacteremia increases the permeability of the blood-brain barrier via the Mfsd2a/Caveolin-1 mediated transcytosis pathway. Int. J. Oral Sci. 15, 1–12. doi: 10.1038/s41368-022-00215-y 36631446 PMC9834243

[ref216] Lendel C. Bjerring M. Dubnovitsky A. Kelly R. T. Filippov A. Antzutkin O. N.. (2014). A hexameric peptide barrel as building block of amyloid-β protofibrils. Angew. Chem. Int. Ed. Engl. 53, 12756–12760. doi: 10.1002/anie.201406357, PMID: 25256598

[ref217] Leng S. Zhang W. Zheng Y. Liberman Z. Rhodes C. J. Eldar-Finkelman H.. (2010). Glycogen synthase kinase 3β mediates high glucose-induced ubiquitination and proteasome degradation of insulin receptor substrate 1. J. Endocrinol. 206, 171–181. doi: 10.1677/JOE-09-0456 20466847 PMC3072280

[ref218] Li Y.-Q. Guo C. (2021). A review on lactoferrin and central nervous system diseases. Cells 10:1810. doi: 10.3390/cells10071810, PMID: 34359979 PMC8307123

[ref219] Li L. Michel R. Cohen J. DeCarlo A. Kozarov E. (2008). Intracellular survival and vascular cell-to-cell transmission of *Porphyromonas gingivalis*. BMC Microbiol. 8:26. doi: 10.1186/1471-2180-8-26 18254977 PMC2259307

[ref220] Li D. Ren T. Li H. Liao G. Zhang X. (2022). *Porphyromonas gingivalis*: a key role in Parkinson’s disease with cognitive impairment? Front. Neurol. 13:945523. doi: 10.3389/fneur.2022.945523 35959396 PMC9363011

[ref221] Li Y. Yang C. Wang S. Yang D. Zhang Y. Xu L.. (2020). Copper and iron ions accelerate the prion-like propagation of α-synuclein: a vicious cycle in Parkinson’s disease. Int. J. Biol. Macromol. 163, 562–573. doi: 10.1016/j.ijbiomac.2020.06.274, PMID: 32629061

[ref222] Li X. Zhang J. Li D. He C. He K. Xue T.. (2021). Astrocytic ApoE reprograms neuronal cholesterol metabolism and histone-acetylation-mediated memory. Neuron 109, 957–970.e8. doi: 10.1016/j.neuron.2021.01.005, PMID: 33503410

[ref223] Lim Y. Y. Mormino E. C. Weiner M. Aisen P. Weiner M. Aisen P.. (2017). *APOE* genotype and early β-amyloid accumulation in older adults without dementia. Neurology 89, 1028–1034. doi: 10.1212/WNL.0000000000004336 28794245 PMC5589795

[ref224] Linard M. Letenneur L. Garrigue I. Doize A. Dartigues J.-F. Helmer C. (2020). Interaction between *APOE4* and herpes simplex virus type 1 in Alzheimer’s disease. Alzheimers Dement. 16, 200–208. doi: 10.1002/alz.12008 31914220

[ref225] Little C. S. Hammond C. J. MacIntyre A. Balin B. J. Appelt D. M. (2004). *Chlamydia pneumoniae* induces Alzheimer-like amyloid plaques in brains of BALB/c mice. Neurobiol. Aging 25, 419–429. doi: 10.1016/S0197-4580(03)00127-1, PMID: 15013562

[ref226] Litvinchuk A. Suh J. H. Guo J. L. Lin K. Davis S. S. Bien-Ly N.. (2024). Amelioration of tau and ApoE4-linked glial lipid accumulation and neurodegeneration with an LXR agonist. Neuron 112, 384–403.e8. doi: 10.1016/j.neuron.2023.10.023, PMID: 37995685 PMC10922706

[ref227] Liu M. Sui D. Dexheimer T. Hovde S. Deng X. Wang K.-W.. (2020). Hyperphosphorylation renders tau prone to aggregate and to cause cell death. Mol. Neurobiol. 57, 4704–4719. doi: 10.1007/s12035-020-02034-w, PMID: 32780352 PMC7530023

[ref228] Liu Z. Wang M. Zhang C. Zhou S. Ji G. (2022). Molecular functions of ceruloplasmin in metabolic disease pathology. Diab. Metab. Syndr. Obes. 15, 695–711. doi: 10.2147/DMSO.S346648, PMID: 35264864 PMC8901420

[ref229] Liu Y. Wu Z. Nakanishi Y. Ni J. Hayashi Y. Takayama F.. (2017). Infection of microglia with *Porphyromonas gingivalis* promotes cell migration and an inflammatory response through the gingipain-mediated activation of protease-activated receptor-2 in mice. Sci. Rep. 7, 1–13. doi: 10.1038/s41598-017-12173-1 28924232 PMC5603557

[ref230] Loch R. A. Wang H. Perálvarez-Marín A. Berger P. Nielsen H. Chroni A.. (2023). Cross interactions between apolipoprotein E and amyloid proteins in neurodegenerative diseases. Comput. Struct. Biotechnol. J. 21, 1189–1204. doi: 10.1016/j.csbj.2023.01.022, PMID: 36817952 PMC9932299

[ref231] Lopez M. J. Royer A. Shah N. J. (2023). Biochemistry, ceruloplasmin. Treasure Island, (FL): StatPearls Publishing. 32119309

[ref232] Lotz S. K. Blackhurst B. M. Reagin K. L. Funk K. E. (2021). Microbial infections are a risk factor for neurodegenerative diseases. Front. Cell. Neurosci. 15:691136. doi: 10.3389/fncel.2021.691136 34305533 PMC8292681

[ref233] Lubbers J. R. Chauan S. Bianchine J. R. (1982). Controlled clinical evaluations of chlorine dioxide, chlorite and chlorate in man. Environ. Health Perspect. 46, 57–62. doi: 10.1289/ehp.824657, PMID: 6961033 PMC1569027

[ref234] Lukács M. Szunyog G. Grenács Á. Lihi N. Kállay C. Di Natale G.. (2019). Copper(II) coordination abilities of the tau protein’s N-terminus peptide fragments: A combined potentiometric, spectroscopic and mass spectrometric study. ChemPlusChem 84, 1697–1708. doi: 10.1002/cplu.201900504, PMID: 31943878

[ref235] Lundmark K. Westermark G. T. Olsén A. Westermark P. (2005). Protein fibrils in nature can enhance amyloid protein A amyloidosis in mice: cross-seeding as a disease mechanism. Proc. Natl. Acad. Sci. USA 102, 6098–6102. doi: 10.1073/pnas.0501814102, PMID: 15829582 PMC1087940

[ref236] Luo Y. Song Y. (2021). Mechanism of antimicrobial peptides: antimicrobial, anti-inflammatory and antibiofilm activities. Int. J. Mol. Sci. 22:11401. doi: 10.3390/ijms222111401, PMID: 34768832 PMC8584040

[ref237] MacDonald A. B. (2006). Plaques of Alzheimer’s disease originate from cysts of *Borrelia burgdorferi*, the Lyme disease spirochete. Med. Hypotheses 67, 592–600. doi: 10.1016/j.mehy.2006.02.035 16675154

[ref238] Makowska J. Wyrzykowski D. Kamysz E. Tesmar A. Kamysz W. Chmurzyński L. (2019). Probing the binding selected metal ions and biologically active substances to the antimicrobial peptide LL-37 using DSC, ITC measurements and calculations. J. Therm. Anal. Calorim. 138, 4523–4529. doi: 10.1007/s10973-019-08310-9

[ref239] Malaguarnera M. Bella R. Alagona G. Ferri R. Carnemolla A. Pennisi G. (2004). Helicobacter pylori and Alzheimer’s disease: a possible link. Eur. J. Intern. Med. 15, 381–386. doi: 10.1016/j.ejim.2004.05.008, PMID: 15522573

[ref240] Mangalmurti A. Lukens J. R. (2022). How neurons die in Alzheimer’s disease: implications for neuroinflammation. Curr. Opin. Neurobiol. 75:102575. doi: 10.1016/j.conb.2022.102575, PMID: 35691251 PMC9380082

[ref241] Marcinkiewicz J. Kurnyta M. Biedroń R. Bobek M. Kontny E. Maśliński W. (2006). “Anti-inflammatory effects of taurine derivatives (taurine chloramine, taurine bromamine, and taurolidine) are mediated by different mechanisms” in Taurine 6. Eds. S. S. Oja, P. Saransaari (Boston, MA: Springer US), 481–492. 10.1007/978-0-387-33504-9_54 17153635

[ref242] Marineau A. Khan K. A. Servant M. J. (2020). Roles of GSK-3 and β-catenin in antiviral innate immune sensing of nucleic acids. Cells 9:897. doi: 10.3390/cells9040897, PMID: 32272583 PMC7226782

[ref243] Marschallinger J. Iram T. Zardeneta M. Lee S. E. Lehallier B. Haney M. S.. (2020). Lipid-droplet-accumulating microglia represent a dysfunctional and proinflammatory state in the aging brain. Nat. Neurosci. 23, 194–208. doi: 10.1038/s41593-019-0566-1, PMID: 31959936 PMC7595134

[ref244] Marshall B. Adams P. C. (2008). *Helicobacter pylori*: a Nobel pursuit? Can. J. Gastroenterol. 22, 895–896. doi: 10.1155/2008/459810 19018331 PMC2661189

[ref245] Martiskainen H. Haapasalo A. Kurkinen K. M. A. Pihlajamäki J. Soininen H. Hiltunen M. (2013). Targeting ApoE4/ApoE receptor LRP1 in Alzheimer’s disease. Expert Opin. Ther. Targets 17, 781–794. doi: 10.1517/14728222.2013.789862, PMID: 23573918

[ref246] Mauch D. H. Nägler K. Schumacher S. Göritz C. Müller E.-C. Otto A.. (2001). CNS synaptogenesis promoted by glia-derived cholesterol. Science 294, 1354–1357. doi: 10.1126/science.294.5545.1354, PMID: 11701931

[ref247] Mazaheritehrani E. Sala A. Orsi C. F. Neglia R. G. Morace G. Blasi E.. (2014). Human pathogenic viruses are retained in and released by *Candida albicans* biofilm in vitro. Virus Res. 179, 153–160. doi: 10.1016/j.virusres.2013.10.018, PMID: 24184317

[ref248] Mazza M. Marano G. Traversi G. Bria P. Mazza S. (2011). Primary cerebral blood flow deficiency and Alzheimer’s disease: shadows and lights. J. Alzheimers Dis. 23, 375–389. doi: 10.3233/JAD-2010-090700, PMID: 21098977

[ref249] Mendez M. F. (2019). Early-onset Alzheimer disease and its variants. Continuum 25, 34–51. doi: 10.1212/CON.0000000000000687, PMID: 30707186 PMC6538053

[ref250] Meneses A. Koga S. O’Leary J. Dickson D. W. Bu G. Zhao N. (2021). TDP-43 pathology in Alzheimer’s disease. Mol. Neurodegener. 16:84. doi: 10.1186/s13024-021-00503-x, PMID: 34930382 PMC8691026

[ref251] Meng J. X. Zhang Y. Saman D. Haider A. M. De S. Sang J. C.. (2022). Hyperphosphorylated tau self-assembles into amorphous aggregates eliciting TLR4-dependent responses. Nat. Commun. 13, 1–16. doi: 10.1038/s41467-022-30461-x 35577786 PMC9110413

[ref252] Michiels E. Roose K. Gallardo R. Khodaparast L. Khodaparast L. van der Kant R.. (2020). Reverse engineering synthetic antiviral amyloids. Nat. Commun. 11:2832. doi: 10.1038/s41467-020-16721-8, PMID: 32504029 PMC7275043

[ref253] Miklossy J. (2011). Alzheimer’s disease - a neurospirochetosis. Analysis of the evidence following Koch’s and Hill’s criteria. J. Neuroinflammation 8:90. doi: 10.1186/1742-2094-8-90 21816039 PMC3171359

[ref254] Miklossy J. (2015). Historic evidence to support a causal relationship between spirochetal infections and Alzheimer’s disease. Front. Aging Neurosci. 7:46. doi: 10.3389/fnagi.2015.00046 25932012 PMC4399390

[ref255] Miyata M. Smith J. D. (1996). Apolipoprotein E allele–specific antioxidant activity and effects on cytotoxicity by oxidative insults and β–amyloid peptides. Nat. Genet. 14, 55–61. doi: 10.1038/ng0996-55, PMID: 8782820

[ref256] Mohamed W. A. Salama R. M. Schaalan M. F. (2019). A pilot study on the effect of lactoferrin on Alzheimer’s disease pathological sequelae: impact of the p-Akt/PTEN pathway. Biomed. Pharmacother. 111, 714–723. doi: 10.1016/j.biopha.2018.12.118, PMID: 30611996

[ref257] Mohsen S. Dickinson J. A. Somayaji R. (2020). Update on the adverse effects of antimicrobial therapies in community practice. Can. Fam. Physician 66, 651–659, PMID: 32933978 PMC7491661

[ref258] Moir R. D. Atwood C. S. Romano D. M. Laurans M. H. Huang X. Bush A. I.. (1999). Differential effects of apolipoprotein E isoforms on metal-induced aggregation of Aβ using physiological concentrations. Biochemistry 38, 4595–4603. doi: 10.1021/bi982437d 10194381

[ref259] Moloney C. M. Lowe V. J. Murray M. E. (2021). Visualization of neurofibrillary tangle maturity in Alzheimer’s disease: a clinicopathologic perspective for biomarker research. Alzheimers Dement. 17, 1554–1574. doi: 10.1002/alz.12321, PMID: 33797838 PMC8478697

[ref260] Montagne A. Nikolakopoulou A. M. Huuskonen M. T. Sagare A. P. Lawson E. J. Lazic D.. (2021). APOE4 accelerates advanced-stage vascular and neurodegenerative disorder in old Alzheimer’s mice via cyclophilin A independently of amyloid-β. Nat. Aging. 1, 506–520. doi: 10.1038/s43587-021-00073-z, PMID: 35291561 PMC8920485

[ref261] Morgado I. Garvey M. (2015). “Lipids in amyloid-β processing, aggregation, and toxicity” in Advances in experimental medicine and biology. Ed. O. Gursky (Cham: Springer International Publishing), 67–94. 10.1007/978-3-319-17344-3_3 26149926

[ref262] Mossmann D. Vögtle F.-N. Taskin A. A. Teixeira P. F. Ring J. Burkhart J. M.. (2014). Amyloid-β peptide induces mitochondrial dysfunction by inhibition of preprotein maturation. Cell Metab. 20, 662–669. doi: 10.1016/j.cmet.2014.07.024 25176146

[ref263] Murphy D. B. Johnson K. A. Borisy G. G. (1977). Role of tubulin-associated proteins in microtubule nucleation and elongation. J. Mol. Biol. 117, 33–52. doi: 10.1016/0022-2836(77)90021-3, PMID: 599568

[ref264] Murray M. M. Bernstein S. L. Nyugen V. Condron M. M. Teplow D. B. Bowers M. T. (2009). Amyloid β protein: Aβ40 inhibits Aβ42 oligomerization. J. Am. Chem. Soc. 131, 6316–6317. doi: 10.1021/ja8092604, PMID: 19385598 PMC2697393

[ref265] Naidu S. A. G. Wallace T. C. Davies K. J. A. Naidu A. S. (2023). Lactoferrin for mental health: neuro-redox regulation and neuroprotective effects across the blood-brain barrier with special reference to neuro-COVID-19. J. Diet. Suppl. 20, 218–253. doi: 10.1080/19390211.2021.1922567, PMID: 33977807

[ref266] Narasimhan S. Holtzman D. M. Apostolova L. G. Cruchaga C. Masters C. L. Hardy J.. (2024). Apolipoprotein E in Alzheimer’s disease trajectories and the next-generation clinical care pathway. Nat. Neurosci. 27, 1236–1252. doi: 10.1038/s41593-024-01669-5 38898183

[ref267] Nascimento J. C. R. Matos G. A. Pereira L. C. Mourão A. E. C. C. B. Sampaio A. M. Oriá R. B.. (2020). Impact of apolipoprotein E genetic polymorphisms on liver disease: an essential review. Ann. Hepatol. 19, 24–30. doi: 10.1016/j.aohep.2019.07.011 31548169

[ref268] National Institute on Aging. How is Alzheimer’s disease treated?. (2024). Available at: https://www.nia.nih.gov/health/alzheimers-treatment/how-alzheimers-disease-treated

[ref269] National Institute on Aging. NIA-funded active Alzheimer’s and related dementias clinical trials and studies. (2024). Available at: https://www.nia.nih.gov/research/ongoing-AD-trials

[ref270] Naughton S. X. Raval U. Pasinetti G. M. (2020). The viral hypothesis in Alzheimer’s disease: novel insights and pathogen-based biomarkers. J. Pers. Med. 10:74. doi: 10.3390/jpm10030074 32751069 PMC7563893

[ref271] Navarro S. Ventura S. (2022). Computational methods to predict protein aggregation. Curr. Opin. Struct. Biol. 73:102343. doi: 10.1016/j.sbi.2022.102343 35240456

[ref272] Neddens J. Temmel M. Flunkert S. Kerschbaumer B. Hoeller C. Loeffler T.. (2018). Phosphorylation of different tau sites during progression of Alzheimer’s disease. Acta Neuropathol. Commun. 6:52. doi: 10.1186/s40478-018-0557-6, PMID: 29958544 PMC6027763

[ref273] Newell M. E. Adhikari S. Halden R. U. (2022). Systematic and state-of the science review of the role of environmental factors in amyotrophic lateral sclerosis (ALS) or Lou Gehrig’s disease. Sci. Total Environ. 817:152504. doi: 10.1016/j.scitotenv.2021.152504 34971691

[ref274] Nichols E. Steinmetz J. D. Vollset S. E. Fukutaki K. Chalek J. Abd-Allah F.. (2022). Estimation of the global prevalence of dementia in 2019 and forecasted prevalence in 2050: an analysis for the global burden of disease study 2019. Lancet Public Health 7, e105–e125. doi: 10.1016/S2468-2667(21)00249-8, PMID: 34998485 PMC8810394

[ref275] Norins L. C. (2022). Down syndrome and Alzheimer’s disease: same infectious cause, same preventive? Med. Hypotheses 158:110745. doi: 10.1016/j.mehy.2021.110745

[ref276] Norviel V. A. McGrath M. S. Treatment of neurodegenerative disease with sodium chlorite. US Patent. 11938147, (2024). Available at: https://patents.google.com/patent/US11938147B2/en

[ref277] Nunan J. Small D. H. (2000). Regulation of APP cleavage by α-, β- and γ-secretases. FEBS Lett. 483, 6–10. doi: 10.1016/S0014-5793(00)02076-7 11033346

[ref278] Oblak A. L. Lin P. B. Kotredes K. P. Pandey R. S. Garceau D. Williams H. M.. (2021). Comprehensive evaluation of the 5XFAD mouse model for preclinical testing applications: a MODEL-AD study. Front. Aging Neurosci. 13:713726. doi: 10.3389/fnagi.2021.713726 34366832 PMC8346252

[ref279] Oddo S. Caccamo A. Smith I. F. Green K. N. LaFerla F. M. (2006). A dynamic relationship between intracellular and extracellular pools of Aβ. Am. J. Pathol. 168, 184–194. doi: 10.2353/ajpath.2006.050593, PMID: 16400022 PMC1592652

[ref280] Ozaki K. Leonard W. J. (2002). Cytokine and cytokine receptor pleiotropy and redundancy. J. Biol. Chem. 277, 29355–29358. doi: 10.1074/jbc.R200003200, PMID: 12072446

[ref281] Pagani L. Eckert A. (2011). Amyloid-beta interaction with mitochondria. Int. J. Alzheimers Dis. 2011, 1–12. doi: 10.4061/2011/925050 PMC3065051 21461357

[ref282] Panza F. Lozupone M. Solfrizzi V. Watling M. Imbimbo B. P. (2019). Time to test antibacterial therapy in Alzheimer’s disease. Brain 142, 2905–2929. doi: 10.1093/brain/awz244, PMID: 31532495

[ref283] Parihar M. S. Brewer G. J. (2010). Amyloid-β as a modulator of synaptic plasticity. J. Alzheimers Dis. 22, 741–763. doi: 10.3233/JAD-2010-101020, PMID: 20847424 PMC3079354

[ref284] Park S.-C. Moon J. C. Shin S. Y. Son H. Jung Y. J. Kim N.-H.. (2016). Functional characterization of alpha-synuclein protein with antimicrobial activity. Biochem. Biophys. Res. Commun. 478, 924–928. doi: 10.1016/j.bbrc.2016.08.052 27520375

[ref285] Parker W. D. Jr. Ba J. P. Filley C. M. Kleinschmidt-DeMasters B. K. (1994). Electron transport chain defects in Alzheimer’s disease brain. Neurology 44:1090. doi: 10.1212/WNL.44.6.1090 8208407

[ref286] Pasupuleti M. Roupe M. Rydengård V. Surewicz K. Surewicz W. K. Chalupka A.. (2009). Antimicrobial activity of human prion protein is mediated by its N-terminal region. PLoS One 4:e7358. doi: 10.1371/journal.pone.0007358, PMID: 19809501 PMC2752989

[ref287] Perez M. Santa-Maria I. De Barreda E. G. Zhu X. Cuadros R. Cabrero J. R.. (2009). Tau – an inhibitor of deacetylase HDAC6 function. J. Neurochem. 109, 1756–1766. doi: 10.1111/j.1471-4159.2009.06102.x 19457097

[ref288] Petruk G. Elvén M. Hartman E. Davoudi M. Schmidtchen A. Puthia M.. (2021). The role of full-length apoE in clearance of gram-negative bacteria and their endotoxins. J. Lipid Res. 62:100086. doi: 10.1016/j.jlr.2021.100086, PMID: 34019903 PMC8225977

[ref289] Pickart L. Vasquez-Soltero J. Margolina A. (2017). The effect of the human peptide GHK on gene expression relevant to nervous system function and cognitive decline. Brain Sci. 7:20. doi: 10.3390/brainsci7020020 28212278 PMC5332963

[ref290] Piekut T. Hurła M. Banaszek N. Szejn P. Dorszewska J. Kozubski W.. (2022). Infectious agents and Alzheimer’s disease. J. Integr. Neurosci. 21:073. doi: 10.31083/j.jin2102073 35364661

[ref291] Pino A. Giunta G. Randazzo C. L. Caruso S. Caggia C. Cianci A. (2017). Bacterial biota of women with bacterial vaginosis treated with lactoferrin: an open prospective randomized trial. Microb. Ecol. Health Dis. 28:1357417. doi: 10.1080/16512235.2017.1357417, PMID: 28959181 PMC5614382

[ref292] Poole C. L. James S. H. (2018). Antiviral therapies for herpesviruses: current agents and new directions. Clin. Ther. 40, 1282–1298. doi: 10.1016/j.clinthera.2018.07.006, PMID: 30104016 PMC7728158

[ref293] Poole S. Singhrao S. K. Kesavalu L. Curtis M. A. Crean S. (2013). Determining the presence of periodontopathic virulence factors in short-term postmortem Alzheimer’s disease brain tissue. J. Alzheimers Dis. 36, 665–677. doi: 10.3233/JAD-121918, PMID: 23666172

[ref294] Posadas Y. Sánchez-López C. Quintanar L. (2023). Copper binding and protein aggregation: a journey from the brain to the human lens. RSC Chem. Biol. 4, 974–985. doi: 10.1039/D3CB00145H, PMID: 38033729 PMC10685798

[ref295] Pourvali K. Matak P. Latunde-Dada G. O. Solomou S. Mastrogiannaki M. Peyssonnaux C.. (2012). Basal expression of copper transporter 1 in intestinal epithelial cells is regulated by hypoxia-inducible factor 2α. FEBS Lett. 586, 2423–2427. doi: 10.1016/j.febslet.2012.05.058, PMID: 22684009

[ref296] Powell-Doherty R. D. Abbott A. R. N. Nelson L. A. Bertke A. S. (2020). Amyloid-β and p-tau anti-threat response to herpes simplex virus 1 infection in primary adult murine hippocampal neurons. J. Virol. 94:e01874-19. doi: 10.1128/JVI.01874-19 32075924 PMC7163132

[ref297] Prosswimmer T. Heng A. Daggett V. (2024). Mechanistic insights into the role of amyloid-β in innate immunity. Sci. Rep. 14, 1–11. doi: 10.1038/s41598-024-55423-9 38438446 PMC10912764

[ref298] Prudent M. Girault H. H. (2009). The role of copper in cysteine oxidation: study of intra- and inter-molecular reactions in mass spectrometry. Metallomics 1, 157–165. doi: 10.1039/B817061D 21305109

[ref299] PubChem. Use of a chemically-stabilized chlorite matrix for the parenteral treatment of HIV infections. (2024). Patent US-6086922-A - PubChem. Available at: https://pubchem.ncbi.nlm.nih.gov/patent/US6086922

[ref300] Puthia M. Marzinek J. K. Petruk G. Ertürk Bergdahl G. Bond P. J. Petrlova J. (2022). Antibacterial and anti-inflammatory effects of apolipoprotein E. Biomedicines 10:1430. doi: 10.3390/biomedicines10061430, PMID: 35740451 PMC9220183

[ref301] Puzzo D. Privitera L. Fa M. Staniszewski A. Hashimoto G. Aziz F.. (2011). Endogenous amyloid-β is necessary for hippocampal synaptic plasticity and memory. Ann. Neurol. 69, 819–830. doi: 10.1002/ana.22313, PMID: 21472769 PMC4071456

[ref302] Puzzo D. Privitera L. Leznik E. Fà M. Staniszewski A. Palmeri A.. (2008). Picomolar amyloid-β positively modulates synaptic plasticity and memory in hippocampus. J. Neurosci. 28, 14537–14545. doi: 10.1523/JNEUROSCI.2692-08.2008, PMID: 19118188 PMC2673049

[ref303] Qin Y. Liu Q. Tian S. Xie W. Cui J. Wang R.-F. (2016). TRIM9 short isoform preferentially promotes DNA and RNA virus-induced production of type I interferon by recruiting GSK3β to TBK1. Cell Res. 26, 613–628. doi: 10.1038/cr.2016.27, PMID: 26915459 PMC4856760

[ref304] Qiu T. Liu Q. Chen Y.-X. Zhao Y.-F. Li Y.-M. (2015). A*β*42 and A*β*40: similarities and differences. J. Pept. Sci. 21, 522–529. doi: 10.1002/psc.2789 26018760

[ref305] Rabinovici G. D. (2021). Controversy and progress in Alzheimer’s disease — FDA approval of aducanumab. N. Engl. J. Med. 385, 771–774. doi: 10.1056/NEJMp2111320, PMID: 34320284

[ref306] Rao R. V. Subramaniam K. G. Gregory J. Bredesen A. L. Coward C. Okada S.. (2023). Rationale for a multi-factorial approach for the reversal of cognitive decline in Alzheimer’s disease and MCI: a review. Int. J. Mol. Sci. 24:1659. doi: 10.3390/ijms24021659, PMID: 36675177 PMC9865291

[ref307] Rapaka D. Adiukwu P. C. Bitra V. R. (2022). Experimentally induced animal models for cognitive dysfunction and Alzheimer’s disease. Methods X 9:101933. doi: 10.1016/j.mex.2022.101933, PMID: 36479589 PMC9720010

[ref308] Raulin A.-C. Doss S. V. Trottier Z. A. Ikezu T. C. Bu G. Liu C.-C. (2022). ApoE in Alzheimer’s disease: pathophysiology and therapeutic strategies. Mol. Neurodegener. 17:72. doi: 10.1186/s13024-022-00574-4 36348357 PMC9644639

[ref309] Readhead B. Haure-Mirande J.-V. Funk C. C. Richards M. A. Shannon P. Haroutunian V.. (2018). Multiscale analysis of independent Alzheimer’s cohorts finds disruption of molecular, genetic, and clinical networks by human herpesvirus. Neuron 99, 64–82.e7. doi: 10.1016/j.neuron.2018.05.023, PMID: 29937276 PMC6551233

[ref310] Ridyard K. E. Overhage J. (2021). The potential of human peptide LL-37 as an antimicrobial and anti-biofilm agent. Antibiotics 10:650. doi: 10.3390/antibiotics10060650, PMID: 34072318 PMC8227053

[ref311] Rivers-Auty J. Mather A. E. Peters R. Lawrence C. B. Brough D. (2020). Anti-inflammatories in Alzheimer’s disease—potential therapy or spurious correlate? Brain Commun. 2:fcaa109. doi: 10.1093/braincomms/fcaa109 33134914 PMC7585697

[ref312] Riviere G. R. Riviere K. H. Smith K. S. (2002). Molecular and immunological evidence of oral *Treponema* in the human brain and their association with Alzheimer’s disease. Oral Microbiol. Immunol. 17, 113–118. doi: 10.1046/j.0902-0055.2001.00100.x 11929559

[ref313] Roger A. J. Muñoz-Gómez S. A. Kamikawa R. (2017). The origin and diversification of mitochondria. Curr. Biol. 27, R1177–R1192. doi: 10.1016/j.cub.2017.09.015 29112874

[ref314] Ruiz L. M. Libedinsky A. Elorza A. A. (2021). Role of copper on mitochondrial function and metabolism. Front. Mol. Biosci. 8:711227. doi: 10.3389/fmolb.2021.711227 34504870 PMC8421569

[ref315] Ryder M. I. (2020). *Porphyromonas gingivalis* and Alzheimer disease: recent findings and potential therapies. J. Periodontol. 91:S45. doi: 10.1002/JPER.20-0104 32533852 PMC7689719

[ref316] Summers K. L. Roseman G. Schilling K. M. Dolgova N. V. Pushie M. J. Sokaras D.. (2022). Alzheimer’s drug PBT2 interacts with the amyloid β 1–42 peptide differently than other 8-hydroxyquinoline chelating drugs. Inorg. Chem. 61, 14626–14640. doi: 10.1021/acs.inorgchem.2c01694, PMID: 36073854 PMC9957665

[ref317] Safieh M. Korczyn A. D. Michaelson D. M. (2019). ApoE4: an emerging therapeutic target for Alzheimer’s disease. BMC Med. 17:64. doi: 10.1186/s12916-019-1299-4, PMID: 30890171 PMC6425600

[ref318] Saleh D. Yarrarapu S. N. S. Sharma S. (2023). Herpes simplex type 1. Treasure Island, (FL): StatPearls Publishing. 29489260

[ref319] Salzano G. Giachin G. Legname G. (2019). Structural consequences of copper binding to the prion protein. Cells 8:770. doi: 10.3390/cells8080770, PMID: 31349611 PMC6721516

[ref320] Sancho-Vaello E. Gil-Carton D. François P. Bonetti E.-J. Kreir M. Pothula K. R.. (2020). The structure of the antimicrobial human cathelicidin LL-37 shows oligomerization and channel formation in the presence of membrane mimics. Sci. Rep. 10, 1–16. doi: 10.1038/s41598-020-74401-5 33060695 PMC7562864

[ref321] Sanford S. A. I. McEwan W. A. (2022). Type-I interferons in Alzheimer’s disease and other tauopathies. Front. Cell Neurosci. 16:949340. doi: 10.3389/fncel.2022.949340 35910253 PMC9334774

[ref322] Santonocito S. Giudice A. Polizzi A. Troiano G. Merlo E. M. Sclafani R.. (2022). A cross-talk between diet and the oral microbiome: balance of nutrition on inflammation and immune system’s response during periodontitis. Nutrients 14:2426. doi: 10.3390/nu14122426, PMID: 35745156 PMC9227938

[ref323] Sapi E. Bastian S. L. Mpoy C. M. Scott S. Rattelle A. Pabbati N.. (2012). Characterization of biofilm formation by *Borrelia burgdorferi in vitro*. PLoS One 7:e48277. doi: 10.1371/journal.pone.0048277, PMID: 23110225 PMC3480481

[ref324] Sapi E. Gupta K. Wawrzeniak K. Gaur G. Torres J. Filush K.. (2019). Borrelia and Chlamydia can form mixed biofilms in infected human skin tissues. Eur. J. Microbiol. Immunol. 9, 46–55. doi: 10.1556/1886.2019.00003, PMID: 31223496 PMC6563687

[ref325] Sasaguri H. Nilsson P. Hashimoto S. Nagata K. Saito T. De Strooper B.. (2017). APP mouse models for Alzheimer’s disease preclinical studies. EMBO J. 36, 2473–2487. doi: 10.15252/embj.201797397 28768718 PMC5579350

[ref326] Sasanian N. Bernson D. Horvath I. Wittung-Stafshede P. Esbjörner E. K. (2020). Redox-dependent copper ion modulation of amyloid-β (1-42) aggregation in vitro. Biomol. Ther. 10:924. doi: 10.3390/biom10060924, PMID: 32570820 PMC7355640

[ref327] Satala D. Gonzalez-Gonzalez M. Smolarz M. Surowiec M. Kulig K. Wronowska E.. (2022). The role of *Candida albicans* virulence factors in the formation of multispecies biofilms with bacterial periodontal pathogens. Front. Cell. Infect. Microbiol. 11:765942. doi: 10.3389/fcimb.2021.765942 35071033 PMC8766842

[ref328] Sayas C. L. Ávila J. (2021). GSK-3 and tau: a key duet in Alzheimer’s disease. Cells 10:721. doi: 10.3390/cells10040721, PMID: 33804962 PMC8063930

[ref329] Scheiber I. F. Dringen R. (2013). Astrocyte functions in the copper homeostasis of the brain. Neurochem. Int. 62, 556–565. doi: 10.1016/j.neuint.2012.08.017 22982300

[ref330] Schmukler E. Solomon S. Simonovitch S. Goldshmit Y. Wolfson E. Michaelson D. M.. (2020). Altered mitochondrial dynamics and function in APOE4-expressing astrocytes. Cell Death Dis. 11, 1–13. doi: 10.1038/s41419-020-02776-4 32709881 PMC7382473

[ref331] Schützmann M. P. Hasecke F. Bachmann S. Zielinski M. Hänsch S. Schröder G. F.. (2021). Endo-lysosomal Aβ concentration and pH trigger formation of Aβ oligomers that potently induce tau missorting. Nat. Commun. 12, 1–14. doi: 10.1038/s41467-021-24900-4 34330900 PMC8324842

[ref332] Seaks C. E. Wilcock D. M. (2020). Infectious hypothesis of Alzheimer disease. PLoS Pathog. 16:e1008596. doi: 10.1371/journal.ppat.1008596, PMID: 33180879 PMC7660461

[ref333] Sędzikowska A. Szablewski L. (2021). Insulin and insulin resistance in Alzheimer’s disease. Int. J. Mol. Sci. 22:9987. doi: 10.3390/ijms22189987, PMID: 34576151 PMC8472298

[ref334] Segre J. A. (2013). What does it take to satisfy Koch’s postulates two centuries later? Microbial genomics and propionibacteria acnes. J. Invest. Dermatol. 133, 2141–2142. doi: 10.1038/jid.2013.260, PMID: 23842116 PMC3775492

[ref335] Sen C. K. Khanna S. Venojarvi M. Trikha P. Ellison E. C. Hunt T. K.. (2002). Copper-induced vascular endothelial growth factor expression and wound healing. Am. J. Physiol. Heart Circ. Physiol. 282, H1821–H1827. doi: 10.1152/ajpheart.01015.2001, PMID: 11959648

[ref336] Senejani A. G. Maghsoudlou J. El-Zohiry D. Gaur G. Wawrzeniak K. Caravaglia C.. (2022). *Borrelia burgdorferi* co-localizing with amyloid markers in Alzheimer’s disease brain tissues. J. Alzheimers Dis. 85, 889–903. doi: 10.3233/JAD-215398, PMID: 34897095 PMC8842785

[ref337] Sharma G. Sharma S. Sharma P. Chandola D. Dang S. Gupta S.. (2016). *Escherichia coli* biofilm: development and therapeutic strategies. J. Appl. Microbiol. 121, 309–319. doi: 10.1111/jam.13078, PMID: 26811181

[ref338] Shimonovich M. Pearce A. Thomson H. Keyes K. Katikireddi S. V. (2021). Assessing causality in epidemiology: revisiting Bradford Hill to incorporate developments in causal thinking. Eur. J. Epidemiol. 36, 873–887. doi: 10.1007/s10654-020-00703-7, PMID: 33324996 PMC8206235

[ref339] Shinohara M. Tachibana M. Kanekiyo T. Bu G. (2017). Role of LRP1 in the pathogenesis of Alzheimer’s disease: evidence from clinical and preclinical studies. J. Lipid Res. 58, 1267–1281. doi: 10.1194/jlr.R075796, PMID: 28381441 PMC5496044

[ref340] Singh I. Sagare A. P. Coma M. Perlmutter D. Gelein R. Bell R. D.. (2013). Low levels of copper disrupt brain amyloid-β homeostasis by altering its production and clearance. Proc. Natl. Acad. Sci. USA 110, 14771–14776. doi: 10.1073/pnas.1302212110, PMID: 23959870 PMC3767519

[ref341] Sivieri K. Bassan J. Peixoto G. Monti R. (2017). Gut microbiota and antimicrobial peptides. Curr. Opin. Food Sci. 13, 56–62. doi: 10.1016/j.cofs.2017.02.010

[ref342] Snyder S. W. Ladror U. S. Wade W. S. Wang G. T. Barrett L. W. Matayoshi E. D.. (1994). Amyloid-beta aggregation: selective inhibition of aggregation in mixtures of amyloid with different chain lengths. Biophys. J. 67, 1216–1228. doi: 10.1016/S0006-3495(94)80591-0, PMID: 7811936 PMC1225478

[ref343] Solier S. Müller S. Cañeque T. Versini A. Mansart A. Sindikubwabo F.. (2023). A druggable copper-signalling pathway that drives inflammation. Nature 617, 386–394. doi: 10.1038/s41586-023-06017-4, PMID: 37100912 PMC10131557

[ref344] Song X. Chen J. Hou Z. Xie N. (2021). Antimicrobial therapy and the potential mechanisms in Alzheimer’s disease. Neurosci. Lett. 741:135464. doi: 10.1016/j.neulet.2020.135464 33166642

[ref345] Sorenson J. R. J. (2001). Prion diseases: copper deficiency states associated with impaired nitrogen monoxide or carbon monoxide transduction and translocation. J. Inorg. Biochem. 87, 125–127. doi: 10.1016/S0162-0134(01)00303-8 11730893

[ref346] Soscia S. J. Kirby J. E. Washicosky K. J. Tucker S. M. Ingelsson M. Hyman B.. (2010). The Alzheimer’s disease-associated amyloid β-protein is an antimicrobial peptide. PLoS One 5:e9505. doi: 10.1371/journal.pone.0009505, PMID: 20209079 PMC2831066

[ref347] Sperling R. A. Donohue M. C. Raman R. Rafii M. S. Johnson K. Masters C. L.. (2023). Trial of solanezumab in preclinical Alzheimer’s disease. N. Engl. J. Med. 389, 1096–1107. doi: 10.1056/NEJMoa2305032, PMID: 37458272 PMC10559996

[ref348] Squitti R. Ghidoni R. Simonelli I. Ivanova I. D. Colabufo N. A. Zuin M.. (2018). Copper dyshomeostasis in Wilson disease and Alzheimer’s disease as shown by serum and urine copper indicators. J. Trace Elem. Med. Biol. 45, 181–188. doi: 10.1016/j.jtemb.2017.11.005, PMID: 29173477

[ref349] Stefaniak E. Atrian-Blasco E. Goch W. Sabater L. Hureau C. Bal W. (2021). The aggregation pattern of Aβ_1–40_ is altered by the presence of *N*-truncated Aβ_4–40_ and/or cu^II^ in a similar way through ionic interactions. Chemistry 27, 2798–2809. doi: 10.1002/chem.202004484 33207022

[ref350] Stefaniak E. Bal W. (2019). Cu^II^ binding properties of N-truncated Aβ peptides: in search of biological function. Inorg. Chem. 58, 13561–13577. doi: 10.1021/acs.inorgchem.9b01399, PMID: 31304745

[ref351] Stuart B. A. R. Franitza A. L. Lezi E. (2022). Regulatory roles of antimicrobial peptides in the nervous system: implications for neuronal aging. Front. Cell Neurosci. 16:843790. doi: 10.3389/fncel.2022.843790, PMID: 35321204 PMC8936185

[ref352] Sun Y.-Y. Wang Z. Huang H.-C. (2023). Roles of ApoE4 on the pathogenesis in Alzheimer’s disease and the potential therapeutic approaches. Cell. Mol. Neurobiol. 43, 3115–3136. doi: 10.1007/s10571-023-01365-1, PMID: 37227619 PMC10211310

[ref353] Sun X.-Y. Wei Y.-P. Xiong Y. Wang X.-C. Xie A.-J. Wang X.-L.. (2012). Synaptic released zinc promotes tau hyperphosphorylation by inhibition of protein phosphatase 2A (PP2A). J. Biol. Chem. 287, 11174–11182. doi: 10.1074/jbc.M111.309070, PMID: 22334661 PMC3322889

[ref354] Takahashi R. H. Nagao T. Gouras G. K. (2017). Plaque formation and the intraneuronal accumulation of β-amyloid in Alzheimer’s disease. Pathol. Int. 67, 185–193. doi: 10.1111/pin.12520 28261941

[ref355] Tan R. H. Kril J. J. Yang Y. Tom N. Hodges J. R. Villemagne V. L.. (2017). Assessment of amyloid β in pathologically confirmed frontotemporal dementia syndromes. Alzheimers Dement 9, 10–20. doi: 10.1016/j.dadm.2017.05.005, PMID: 28653036 PMC5473545

[ref356] Taquet M. Dercon Q. Todd J. A. Harrison P. J. (2024). The recombinant shingles vaccine is associated with lower risk of dementia. Nat. Med., 1–5. doi: 10.1038/s41591-024-03201-5 39053634 PMC11485228

[ref357] Teixeira F. B. Saito M. T. Matheus F. C. Prediger R. D. Yamada E. S. Maia C. S. F.. (2017). Periodontitis and Alzheimer’s disease: a possible comorbidity between oral chronic inflammatory condition and neuroinflammation. Front. Aging Neurosci. 9:327. doi: 10.3389/fnagi.2017.00327, PMID: 29085294 PMC5649154

[ref358] Tellechea P. Pujol N. Esteve-Belloch P. Echeveste B. García-Eulate M. R. Arbizu J.. (2018). Early- and late-onset Alzheimer disease: are they the same entity? Neurol 33, 244–253. doi: 10.1016/j.nrleng.2015.08.009 26546285

[ref359] Tetz G. Pinho M. Pritzkow S. Mendez N. Soto C. Tetz V. (2020). Bacterial DNA promotes tau aggregation. Sci. Rep. 10, 1–11. doi: 10.1038/s41598-020-59364-x 32047247 PMC7012890

[ref360] Therriault J. Benedet A. L. Pascoal T. A. Mathotaarachchi S. Savard M. Chamoun M.. (2021). APOEε4 potentiates the relationship between amyloid-β and tau pathologies. Mol. Psychiatry 26, 5977–5988. doi: 10.1038/s41380-020-0688-6, PMID: 32161362 PMC8758492

[ref361] Tian M. Han Y.-B. Yang G.-Y. Li J.-L. Shi C.-S. Tian D. (2023). The role of lactoferrin in bone remodeling: evaluation of its potential in targeted delivery and treatment of metabolic bone diseases and orthopedic conditions. Front. Endocrinol. 14:1218148. doi: 10.3389/fendo.2023.1218148, PMID: 37680888 PMC10482240

[ref362] Traini E. Carotenuto A. Fasanaro A. M. Amenta F. (2020). Volume analysis of brain cognitive areas in Alzheimer’s disease: interim 3-year results from the ASCOMALVA trial. J. Alzheimers Dis. 76, 317–329. doi: 10.3233/JAD-190623, PMID: 32508323 PMC7369051

[ref363] Trinczek B. Biernat J. Baumann K. Mandelkow E. M. Mandelkow E. (1995). Domains of tau protein, differential phosphorylation, and dynamic instability of microtubules. Mol. Biol. Cell 6, 1887–1902. doi: 10.1091/mbc.6.12.1887, PMID: 8590813 PMC366657

[ref364] Tripathi T. Khan H. (2020). Direct interaction between the β-amyloid core and tau facilitates cross-seeding: a novel target for therapeutic intervention. Biochemistry 59, 341–342. doi: 10.1021/acs.biochem.9b01087, PMID: 31944100

[ref365] Tsatsanis A. McCorkindale A. N. Wong B. X. Patrick E. Ryan T. M. Evans R. W.. (2021). The acute phase protein lactoferrin is a key feature of Alzheimer’s disease and predictor of Aβ burden through induction of APP amyloidogenic processing. Mol. Psychiatry 26, 5516–5531. doi: 10.1038/s41380-021-01248-1, PMID: 34400772 PMC8758478

[ref366] Tümer Z. Møller L. B. (2010). Menkes disease. Eur. J. Hum. Genet. 18, 511–518. doi: 10.1038/ejhg.2009.187, PMID: 19888294 PMC2987322

[ref367] Ulrich G. Salvadè A. Boersema P. Calì T. Foglieni C. Sola M.. (2018). Phosphorylation of nuclear tau is modulated by distinct cellular pathways. Sci. Rep. 8, 1–14. doi: 10.1038/s41598-018-36374-4 30531974 PMC6286375

[ref368] van Exel E. Koopman J. J. E. van Bodegom D. Meij J. J. De K. P. Ziem J. B.. (2017). Effect of APOE ε4 allele on survival and fertility in an adverse environment. PLoS One 12:e0179497. doi: 10.1371/journal.pone.0179497 28683096 PMC5500260

[ref369] Van Giau V. Bagyinszky E. Yang Y. S. Youn Y. C. An S. S. A. Kim S. Y. (2019). Genetic analyses of early-onset Alzheimer’s disease using next generation sequencing. Sci. Rep. 9:8368. doi: 10.1038/s41598-019-44848-2 31182772 PMC6557896

[ref370] Van Gool B. Storck S. E. Reekmans S. M. Lechat B. Gordts P. L. S. M. Pradier L.. (2019). LRP1 has a predominant role in production over clearance of Aβ in a mouse model of Alzheimer’s disease. Mol. Neurobiol. 56, 7234–7245. doi: 10.1007/s12035-019-1594-2, PMID: 31004319 PMC6728278

[ref371] Vanderweyde T. Apicco D. J. Youmans-Kidder K. Ash P. E. A. Cook C. Lummertz da Rocha E.. (2016). Interaction of tau with the RNA-binding protein TIA1 regulates tau pathophysiology and toxicity. Cell Rep. 15, 1455–1466. doi: 10.1016/j.celrep.2016.04.045, PMID: 27160897 PMC5325702

[ref372] Vasefi M. Ghaboolian-Zare E. Abedelwahab H. Osu A. (2020). Environmental toxins and Alzheimer’s disease progression. Neurochem. Int. 141:104852. doi: 10.1016/j.neuint.2020.104852 33010393

[ref373] Vega C. (2021). From Hume to Wuhan: an epistemological journey on the problem of induction in COVID-19 machine learning models and its impact upon medical research. IEEE Access. 9, 97243–97250. doi: 10.1109/ACCESS.2021.3095222 34812399 PMC8545192

[ref374] Vendelboe T. V. Harris P. Zhao Y. Walter T. S. Harlos K. El Omari K.. (2016). The crystal structure of human dopamine β-hydroxylase at 2.9 Å resolution. Sci. Adv. 2:e1500980. doi: 10.1126/sciadv.1500980 27152332 PMC4846438

[ref375] Vera-Aviles M. Vantana E. Kardinasari E. Koh N. Latunde-Dada G. (2018). Protective role of histidine supplementation against oxidative stress damage in the management of anemia of chronic kidney disease. Pharmaceuticals 11:111. doi: 10.3390/ph11040111, PMID: 30347874 PMC6315830

[ref376] Vigasova D. Nemergut M. Liskova B. Damborsky J. (2021). Multi-pathogen infections and Alzheimer’s disease. Microb. Cell Factories 20:25. doi: 10.1186/s12934-021-01520-7, PMID: 33509204 PMC7844946

[ref377] von Bernhardi R. Cornejo F. Parada G. E. Eugenín J. (2015). Role of TGFβ signaling in the pathogenesis of Alzheimer’s disease. Front. Cell. Neurosci. 9:426. doi: 10.3389/fncel.2015.00426, PMID: 26578886 PMC4623426

[ref378] Vonk A. G. Bont N. D. Netea M. G. Demacker P. N. M. Van Der Meer J. W. M. Stalenhoef A. F. H.. (2004). Apolipoprotein-E-deficient mice exhibit an increased susceptibility to disseminated candidiasis. Med. Mycol. 42, 341–348. doi: 10.1080/13693780410001657135, PMID: 15473359

[ref379] Voss K. Harris C. Ralle M. Duffy M. Murchison C. Quinn J. F. (2014). Modulation of tau phosphorylation by environmental copper. Transl. Neurodegener. 3:24. doi: 10.1186/2047-9158-3-24 25671100 PMC4322670

[ref380] Wally J. Buchanan S. K. (2007). A structural comparison of human serum transferrin and human lactoferrin. Biometals 20, 249–262. doi: 10.1007/s10534-006-9062-7, PMID: 17216400 PMC2547852

[ref381] Walsh D. M. Klyubin I. Fadeeva J. V. Cullen W. K. Anwyl R. Wolfe M. S.. (2002). Naturally secreted oligomers of amyloid β protein potently inhibit hippocampal long-term potentiation *in vivo*. Nature 416, 535–539. doi: 10.1038/416535a, PMID: 11932745

[ref382] Wang D. Chen F. Han Z. Yin Z. Ge X. Lei P. (2021). Relationship between amyloid-β deposition and blood–brain barrier dysfunction in Alzheimer’s disease. Front. Cell Neurosci. 15:695479. doi: 10.3389/fncel.2021.695479 34349624 PMC8326917

[ref383] Wang F. Li J. Fan S. Jin Z. Huang C. (2020). Targeting stress granules: a novel therapeutic strategy for human diseases. Pharmacol. Res. 161:105143. doi: 10.1016/j.phrs.2020.105143, PMID: 32814168 PMC7428673

[ref384] Wang X. Ryu D. Houtkooper R. H. Auwerx J. (2015). Antibiotic use and abuse: a threat to mitochondria and chloroplasts with impact on research, health, and environment. BioEssays 37, 1045–1053. doi: 10.1002/bies.201500071, PMID: 26347282 PMC4698130

[ref385] Wang X.-L. Zeng J. Yang Y. Xiong Y. Zhang Z.-H. Qiu M.. (2014). *Helicobacter pylori* filtrate induces Alzheimer-like tau hyperphosphorylation by activating glycogen synthase kinase-3β. J. Alzheimers Dis. 43, 153–165. doi: 10.3233/JAD-140198, PMID: 25079798

[ref386] Ward R. J. Zucca F. A. Duyn J. H. Crichton R. R. Zecca L. (2014). The role of iron in brain ageing and neurodegenerative disorders. Lancet Neurol. 13, 1045–1060. doi: 10.1016/S1474-4422(14)70117-6, PMID: 25231526 PMC5672917

[ref387] Warren J. D. Rohrer J. D. Rossor M. N. (2013). Frontotemporal dementia. BMJ 347:f4827. doi: 10.1136/bmj.f4827 23920254 PMC3735339

[ref388] Weidung B. Hemmingsson E.-S. Olsson J. Sundström T. Blennow K. Zetterberg H.. (2022). VALZ-pilot: high-dose valacyclovir treatment in patients with early-stage Alzheimer’s disease. Alzheimers Dement. 8:e12264. doi: 10.1002/trc2.12264 PMC8919248 35310522

[ref389] Weisgraber K. H. (1990). Apolipoprotein E distribution among human plasma lipoproteins: role of the cysteine-arginine interchange at residue 112. J. Lipid Res. 31, 1503–1511. doi: 10.1016/S0022-2275(20)42621-5, PMID: 2280190

[ref390] Welikovitch L. A. Do Carmo S. Maglóczky Z. Szocsics P. Lőke J. Freund T.. (2018). Evidence of intraneuronal Aβ accumulation preceding tau pathology in the entorhinal cortex. Acta Neuropathol. 136, 901–917. doi: 10.1007/s00401-018-1922-z, PMID: 30362029

[ref391] West T. Kirmess K. M. Meyer M. R. Holubasch M. S. Knapik S. S. Hu Y.. (2021). A blood-based diagnostic test incorporating plasma Aβ42/40 ratio, ApoE proteotype, and age accurately identifies brain amyloid status: findings from a multi cohort validity analysis. Mol. Neurodegener. 16, 1–12. doi: 10.1186/s13024-021-00451-6 33933117 PMC8088704

[ref392] White C. Kambe T. Fulcher Y. G. Sachdev S. W. Bush A. I. Fritsche K.. (2009). Copper transport into the secretory pathway is regulated by oxygen in macrophages. J. Cell Sci. 122, 1315–1321. doi: 10.1242/jcs.043216, PMID: 19351718 PMC2671928

[ref393] White M. R. Kandel R. Tripathi S. Condon D. Qi L. Taubenberger J.. (2014). Alzheimer’s associated β-amyloid protein inhibits influenza A virus and modulates viral interactions with phagocytes. PLoS One 9:e101364. doi: 10.1371/journal.pone.0101364, PMID: 24988208 PMC4079246

[ref394] White C. Lee J. Kambe T. Fritsche K. Petris M. J. (2009). A role for the ATP7A copper-transporting ATPase in macrophage bactericidal activity. J. Biol. Chem. 284, 33949–33956. doi: 10.1074/jbc.M109.070201, PMID: 19808669 PMC2797165

[ref395] Wisniewski T. Drummond E. (2020). APOE-amyloid interaction: therapeutic targets. Neurobiol. Dis. 138:104784. doi: 10.1016/j.nbd.2020.104784, PMID: 32027932 PMC7118587

[ref396] Wormser G. P. Marques A. Pavia C. S. Schwartz I. Feder H. M. Jr. Pachner A. R. (2022). Lack of convincing evidence that *Borrelia burgdorferi* infection causes either Alzheimer disease or Lewy body dementia. Clin. Infect. Dis. 75, 342–346. doi: 10.1093/cid/ciab993, PMID: 34849631 PMC9410724

[ref397] Wozniak M. A. Frost A. L. Itzhaki R. F. (2009). Alzheimer’s disease-specific tau phosphorylation is induced by herpes simplex virus type 1. J. Alzheimers Dis. 16, 341–350. doi: 10.3233/JAD-2009-0963, PMID: 19221424

[ref398] Wozniak M. A. Mee A. P. Itzhaki R. F. (2009). Herpes simplex virus type 1 DNA is located within Alzheimer’s disease amyloid plaques. J. Pathol. 217, 131–138. doi: 10.1002/path.2449, PMID: 18973185

[ref399] Wu H.-Y. Kuo P.-C. Wang Y.-T. Lin H.-T. Roe A. D. Wang B. Y.. (2018). Β-amyloid induces pathology-related patterns of tau hyperphosphorylation at synaptic terminals. J. Neuropathol. Exp. Neurol. 77, 814–826. doi: 10.1093/jnen/nly059, PMID: 30016458

[ref400] Wu L. Su X. Tang Z. Jian L. Zhu H. Cheng X.. (2022). *Treponema denticola* induces neuronal apoptosis by promoting amyloid-β accumulation in mice. Pathogens 11:1150. doi: 10.3390/pathogens11101150, PMID: 36297207 PMC9610539

[ref401] Xia Z. Prescott E. E. Urbanek A. Wareing H. E. King M. C. Olerinyova A.. (2024). Co-aggregation with apolipoprotein E modulates the function of amyloid-β in Alzheimer’s disease. Nat. Commun. 15, 1–18. doi: 10.1038/s41467-024-49028-z 38824138 PMC11144216

[ref402] Xu J. Church S. J. Patassini S. Begley P. Waldvogel H. J. Curtis M. A.. (2017). Evidence for widespread, severe brain copper deficiency in Alzheimer’s dementia. Metallomics 9, 1106–1119. doi: 10.1039/C7MT00074J, PMID: 28654115

[ref403] Xu H. Finkelstein D. I. Adlard P. A. (2014). Interactions of metals and apolipoprotein E in Alzheimerâ’s disease. Front. Aging Neurosci. 6:121. doi: 10.3389/fnagi.2014.00121 24971061 PMC4054654

[ref404] Xu S.-F. Pang Z.-Q. Fan Y.-G. Zhang Y.-H. Meng Y.-H. Bai C.-Y.. (2022). Astrocyte-specific loss of lactoferrin influences neuronal structure and function by interfering with cholesterol synthesis. Glia 70, 2392–2408. doi: 10.1002/glia.24259, PMID: 35946355

[ref405] Xue Y. C. Feuer R. Cashman N. Luo H. (2018). Enteroviral infection: the forgotten link to amyotrophic lateral sclerosis? Front. Mol. Neurosci. 11:63. doi: 10.3389/fnmol.2018.00063 29593492 PMC5857577

[ref406] Yamada M. Ikegami A. Kuramitsu H. K. (2005). Synergistic biofilm formation by*Treponema Denticola*and*porphyromonas gingivalis*. FEMS Microbiol. Lett. 250, 271–277. doi: 10.1016/j.femsle.2005.07.019 16085371

[ref407] Yamazaki Y. Painter M. M. Bu G. Kanekiyo T. (2016). Apolipoprotein E as a therapeutic target in Alzheimer’s disease: a review of basic research and clinical evidence. CNS Drugs 30, 773–789. doi: 10.1007/s40263-016-0361-4, PMID: 27328687 PMC5526196

[ref408] Yamazaki Y. Zhao N. Caulfield T. R. Liu C.-C. Bu G. (2019). Apolipoprotein E and Alzheimer disease: pathobiology and targeting strategies. Nat. Rev. Neurol. 15, 501–518. doi: 10.1038/s41582-019-0228-7, PMID: 31367008 PMC7055192

[ref409] Yang Y.-H. Situmeang R. F. V. Ong P. A. (2021). Can blood amyloid levels be used as a biomarker for Alzheimer’s disease? Brain Sci. Adv. 7, 17–25. doi: 10.26599/BSA.2021.9050004

[ref410] Yankner B. A. Duffy L. K. Kirschner D. A. (1990). Neurotrophic and neurotoxic effects of amyloid β protein: reversal by tachykinin neuropeptides. Science 250, 279–282. doi: 10.1126/science.2218531 2218531

[ref411] Yong S. J. Veerakumarasivam A. Lim W. L. Chew J. (2023). Neuroprotective effects of lactoferrin in Alzheimer’s and Parkinson’s diseases: a narrative review. ACS Chem Neurosci [Internet]. doi: 10.1021/acschemneuro.2c00679, PMID: 36995304

[ref412] Yoon E. J. Park H.-J. Kim G.-Y. Cho H. Choi J.-H. Park H.-Y.. (2009). Intracellular amyloid beta interacts with SOD1 and impairs the enzymatic activity of SOD1: implications for the pathogenesis of amyotrophic lateral sclerosis. Exp. Mol. Med. 41, 611–617. doi: 10.3858/emm.2009.41.9.067, PMID: 19478559 PMC2753654

[ref413] Yuan H. Ma Q. Ye L. Piao G. (2016). The traditional medicine and modern medicine from natural products. Molecules 21:559. doi: 10.3390/molecules21050559, PMID: 27136524 PMC6273146

[ref414] Yugay D. Goronzy D. P. Kawakami L. M. Claridge S. A. Song T.-B. Yan Z.. (2016). Copper ion binding site in β-amyloid peptide. Nano Lett. 16, 6282–6289. doi: 10.1021/acs.nanolett.6b02590 27616333

[ref415] Zeineh M. M. Chen Y. Kitzler H. H. Hammond R. Vogel H. Rutt B. K. (2015). Activated iron-containing microglia in the human hippocampus identified by magnetic resonance imaging in Alzheimer disease. Neurobiol. Aging 36, 2483–2500. doi: 10.1016/j.neurobiolaging.2015.05.022, PMID: 26190634 PMC4839298

[ref416] Zhan X. Stamova B. Jin L.-W. DeCarli C. Phinney B. Sharp F. R. (2016). Gram-negative bacterial molecules associate with Alzheimer disease pathology. Neurology 87, 2324–2332. doi: 10.1212/WNL.0000000000003391, PMID: 27784770 PMC5135029

[ref417] Zhang P. (2022). Influence of foods and nutrition on the gut microbiome and implications for intestinal health. Int. J. Mol. Sci. 23:9588. doi: 10.3390/ijms23179588, PMID: 36076980 PMC9455721

[ref418] Zhang R. Bracci P. M. Azhir A. Forrest B. D. McGrath M. S. (2022). Macrophage-targeted sodium chlorite (NP001) slows progression of amyotrophic lateral sclerosis (ALS) through regulation of microbial translocation. Biomedicines 10:2907. doi: 10.3390/biomedicines10112907, PMID: 36428474 PMC9687998

[ref419] Zhang Y. Gao H. Zheng W. Xu H. (2022). Current understanding of the interactions between metal ions and apolipoprotein E in Alzheimer’s disease. Neurobiol. Dis. 172:105824. doi: 10.1016/j.nbd.2022.105824, PMID: 35878744

[ref420] Zhang X. Song W. (2013). The role of APP and BACE1 trafficking in APP processing and amyloid-β generation. Alzheimers Res. Ther. 5:46. doi: 10.1186/alzrt211, PMID: 24103387 PMC3978418

[ref421] Zhang H. Wei W. Zhao M. Ma L. Jiang X. Pei H.. (2021). Interaction between Aβ and tau in the pathogenesis of Alzheimer’s disease. Int. J. Biol. Sci. 17, 2181–2192. doi: 10.7150/ijbs.57078, PMID: 34239348 PMC8241728

[ref422] Zhang Q.-Y. Yan Z.-B. Meng Y.-M. Hong X.-Y. Shao G. Ma J.-J.. (2021). Antimicrobial peptides: mechanism of action, activity and clinical potential. Mil. Med. Res. 8:48. doi: 10.1186/s40779-021-00343-2 34496967 PMC8425997

[ref423] Zhao M. Ma G. Yan X. Li X. Wang E. Xu X.-X.. (2024). Microbial infection promotes amyloid pathology in a mouse model of Alzheimer’s disease via modulating γ-secretase. Mol. Psychiatry 29, 1491–1500. doi: 10.1038/s41380-024-02428-5 38273109

[ref424] Zhao Y. Wu X. Li X. Jiang L.-L. Gui X. Liu Y.. (2018). TREM2 is a receptor for β-amyloid that mediates microglial function. Neuron 97, 1023–1031.e7. doi: 10.1016/j.neuron.2018.01.031, PMID: 29518356 PMC5889092

[ref425] Zhou Y. Blanco L. P. Smith D. R. Chapman M. R. (2012). “Bacterial amyloids” in Methods in molecular biology. Eds. E. M. Sigurdsson, M. Calero, and M. Gasset (Totowa, NJ: Humana Press), 303–320. 10.1007/978-1-61779-551-0_21 PMC5324733 22528099

[ref426] Zimnicka A. M. Tang H. Guo Q. Kuhr F. K. Oh M.-J. Wan J.. (2014). Upregulated copper transporters in hypoxia-induced pulmonary hypertension. PLoS One 9:e90544. doi: 10.1371/journal.pone.0090544, PMID: 24614111 PMC3948681

[ref427] Zubčić K. Hof P. R. Šimić G. Jazvinšćak J. M. (2020). The role of copper in tau-related pathology in Alzheimer’s disease. Front. Mol. Neurosci. 13:572308. doi: 10.3389/fnmol.2020.572308 33071757 PMC7533614

[ref428] Zuily L. Lahrach N. Fassler R. Genest O. Faller P. Sénèque O.. (2022). Copper induces protein aggregation, a toxic process compensated by molecular chaperones. MBio 13:e0325121. doi: 10.1128/mbio.03251-21, PMID: 35289645 PMC9040851

